# Intake of food rich in saturated fat in relation to subclinical atherosclerosis and potential modulating effects from single genetic variants

**DOI:** 10.1038/s41598-021-86324-w

**Published:** 2021-04-12

**Authors:** Federica Laguzzi, Buamina Maitusong, Rona J. Strawbridge, Damiano Baldassarre, Fabrizio Veglia, Steve E. Humphries, Rainer Rauramaa, Sudhir Kurl, Andries J. Smit, Philippe Giral, Angela Silveira, Elena Tremoli, Anders Hamsten, Ulf de Faire, Bruna Gigante, Karin Leander, IMPROVE Study group, C. R. Sirtori, L. Calabresi, M. Amato, B. Frigerio, A. Ravani, D. Sansaro, C. Tedesco, D. Coggi, N. Capra, A. Bonomi, P. Eriksson, J. Cooper, J. Acharya, K. Savonen, K. Huttunen, E. Rauramaa, I. M. Penttila, J. Törrönen, A. I. van Gessel, A. M. van Roon, A. Nicolai, D. J. Mulder, A. Kontush, A. Carrié, A. Gallo, J. Karppi, T. Nurmi, K. Nyyssönen, T. P. Tuomainen, J. Tuomainen, J. Kauhanen, B. Sennblad, M. Pirro, G. Vaudo, D. Siepi, G. Lupattelli, M. R. Mannarino, V. Bianconi

**Affiliations:** 1 Unit of Cardiovascular and Nutritional Epidemiology, Institute of Environmental Medicine, Karolinska Institutet, Nobels väg 13, Box 210, 17177 Stockholm, Sweden; 2 Department of Cardiology, First Affiliated Hospital of Xinjiang Medical University, Urumqi, People’s Republic of China; 3 Institute of Mental Health and Wellbeing, Mental Health and Wellbeing, University of Glasgow, Glasgow, UK; 4 Cardiovascular Medicine Unit, Department of Medicine Solna, Karolinska Institutet, Stockholm, Sweden; 5 Health Data Research United Kingdom, London, UK; 6 Department of Medical Biotechnology and Translational Medicine, Università degli Studi di Milano, Milan, Italy; 7 Centro Cardiologico Monzino, IRCCS, Milan, Italy; 8 Centre for Cardiovascular Genetics, Institute Cardiovascular Science, University College London, London, UK; 9 Foundation for Research in Health Exercise and Nutrition, Kuopio Research Institute of Exercise Medicine, Kuopio, Finland; 10 Department of Clinical Physiology and Nuclear Medicine, Kuopio University Hospital, Kuopio, Finland; 11 Institute of Public Health and Clinical Nutrition, University of Eastern Finland, Kuopio, Finland; 12 Department of Medicine, University Medical Center Groningen, Groningen, The Netherlands; 13 Assistance Publique-Hôpitaux de Paris, Service Endocrinologie-Métabolisme, Groupe Hospitalier Pitié-Salpétrière, Unités de Prévention Cardiovasculaire, Paris, France; 14 Cardiovascular Medicine Unit, Department of Medicine Solna, Karolinska Institutet and Karolinska Hospital, Stockholm, Sweden; 15 Centro Dislipidemie E. Grossi Paoletti, Ospedale Ca’ Granda di Niguarda, Milan, Italy; 16 National Bioinformatics Infrastructure Sweden, Science for Life Laboratory, Uppsala University, Uppsala, Sweden; 17 Internal Medicine, Angiology and Arteriosclerosis Diseases, Department of Clinical and Experimental Medicine, University of Perugia, Perugia, Italy

**Keywords:** Genetics, Biomarkers, Cardiology, Risk factors

## Abstract

The relationship between intake of saturated fats and subclinical atherosclerosis, as well as the possible influence of genetic variants, is poorly understood and investigated. We aimed to investigate this relationship, with a hypothesis that it would be positive, and to explore whether genetics may modulate it, using data from a European cohort including 3,407 participants aged 54–79 at high risk of cardiovascular disease. Subclinical atherosclerosis was assessed by carotid intima-media thickness (C-IMT), measured at baseline and after 30 months. Logistic regression (OR; 95% CI) was employed to assess the association between high intake of food rich in saturated fat (vs. low) and: (1) the mean and the maximum values of C-IMT in the whole carotid artery (C-IMT_mean_, C-IMT_max_), in the bifurcation (Bif-), the common (CC-) and internal (ICA-) carotid arteries at baseline (binary, cut-point ≥ 75th), and (2) C-IMT progression (binary, cut-point > zero). For the genetic-diet interaction analyses, we considered 100,350 genetic variants. We defined interaction as departure from additivity of effects. After age- and sex-adjustment, high intake of saturated fat was associated with increased C-IMT_mean_ (OR:1.27;1.06–1.47), CC-IMT_mean_ (OR:1.22;1.04–1.44) and ICA-IMT_mean_ (OR:1.26;1.07–1.48). However, in multivariate analysis results were no longer significant. No clear associations were observed between high intake of saturated fat and risk of atherosclerotic progression. There was no evidence of interactions between high intake of saturated fat and any of the genetic variants considered, after multiple testing corrections. High intake of saturated fats was not independently associated with subclinical atherosclerosis. Moreover, we did not identify any significant genetic-dietary fat interactions in relation to risk of subclinical atherosclerosis.

## Introduction

Atherosclerosis, a disease characterized by low-grade, chronic inflammation and accumulation of lipids in the arteries, is the main underlying cause of cardiovascular disease (CVD)^1–3^. The aetiology of atherosclerosis is still not completely understood but seems to involve a complex interplay of traditional CVD modifiable- and non-modifiable risk factors.

Among these traditional risk factors, diet and its content of fats may affect the development of atherosclerosis^4^. A diet high in saturated fat including high intake of meat, dairy products and eggs, has been suggested to increase circulating atherogenic lipoproteins and inflammatory markers^5,6^ and to trigger atherosclerotic development through endothelium dysfunction and plaque rupture vulnerability^5^. Current European and American cardiovascular guidelines recommend to reduce saturated fats and replace them with unsaturated fats^7,8^, based on strong evidence from experimental studies^5^. However, recent epidemiological studies have failed to show an increased risk of CVD in relation to a diet rich in saturated fat^9–14^. Studies investigating the relation between saturated fat intake and surrogate measures of atherosclerosis, mainly carotid intima-media thickness (C-IMT), have shown inconsistent results indicating either increased C-IMT^15–22^ or no associations^23–27^.

Several single nucleotide polymorphism (SNPs) related to subclinical or clinical atherosclerosis have been identified with genome-wide association studies (GWAS)^28^. These studies did not consider interaction effects. The findings have been confirmed by replication only recently^29^. Estimates of heritability of carotid atherosclerosis vary between 20 and 75%^28,30,31^. As suggested from twin studies, the remaining heritability may be explained by interactions between the genetic susceptibility and lifestyle/environmental factors^28,32^.

Studies investigating interactions between genes and dietary fats in relation to atherosclerosis are scarce^33–36^, and focused exclusively on interactions between candidate genes and dietary n-6 and n-3 polyunsaturated fatty acids (PUFA) in relation to carotid atherosclerosis^33,35,36^ or progression of coronary atherosclerosis^34^. Interactions were found for most of the candidate genes under evaluation: arachidonate 5-lipoxygenase (*ALOX5*)^33^, leukotriene A4 hydrolase (*LTA4H*)^35,36^ and sterol regulatory element binding protein-1 (*SREB1*)^34^ but not for insulin-induced gene-1 (*INSIG1*) and SREB cleavage-activating protein * (SCAP*)^34^. To our knowledge, no previous study carried out a genome-wide-screening for genetic variants-dietary fat interactions in relation to atherosclerosis.

In a European multicenter study of men and women, we aimed to investigate the relationship between self-reported intake of food rich in saturated fat and C-IMT. Based on the evidence from experimental studies^5^, we hypothesized a positive relationship. Our aim was also to explore whether genetic predisposition may modulate this association.

## Material and methods

The data that support the findings of this study are available from the corresponding author upon reasonable request.

### Study population

The present study is based on data from the IMPROVE material, a multicenter longitudinal study including 3,703 subjects (48% women), aged 54–79 with at least three CVD risk factors (male sex or at least 5 years after menopause for women, hypercholesterolemia, hypertriglyceridemia, hypoalphalipoproteinemia, hypertension, diabetes or impaired fasting glucose, smoking habits, or family history of cardiovascular diseases) but free of cardiovascular events at enrollment. Details of the study are reported elsewhere^37,38^. In brief, between 2004 and 2005, study participants were recruited in seven centers across five European countries (two centers in Italy and Finland, one center in France, The Netherlands and Sweden). The main objective of the IMPROVE study was to investigate the associations between subclinical atherosclerosis, measured by C-IMT, and future CVD events. Subjects underwent a physical examination, which encompassed sonographic measurements of C-IMT, at baseline and after 30 months. At baseline, they also completed a questionnaire concerning medical history, lifestyle and socioeconomic factors. In addition, blood samples were collected for biochemical testing and genotyping. The study was approved by the Institutional review board (IRB) at each recruitment centre (Karolinska Institutet, Stockholm, Sweden; University of Milan, Milan, Italy; University of Kuopio and Kuopio Research Institute of Exercise Medicine, Kuopio, Finland; University Hospital Groningen, Groningen, The Netherlands; University of Perugia, Perugia, Italy; Groupe Hôpital Pitie-Salpetriere, Paris, France). Written informed consents for general participation and for the genotyping were provided by all participants. The study was conducted in accordance with the Helsinki Declaration.

### Assessment of dietary saturated fat

Participants were asked to recall their consumption, over the last 12 months, of specific items including food rich in fats as follows: milk (ml/day and whether skimmed, semi-skimmed or whole), meat, fish and eggs (times/week) and type of fat and oil usually consumed (lard, butter, olive oil, seed oil, margarine)^37^. From the dietary information collected, we created a binary variable to distinguish between high and low intake of food rich in saturated fat. Such binary exposure variable was required for our chosen method to assess interactions. Detailed description of how the binary dietary variable was formed is given in the Supplementary Materials, Section Materials and Methods. Briefly, it was formed based on a dietary score we created from a selection of the questions on diet in the questionnaire. We chose this approach over other approaches, such as using grams of saturated fat intake per day, because of the advantage of this approach in that it better takes into account the overall diet and therefore to some extent should take into account the complex interplay between nutrients. The binary dietary variable was roughly validated in a subsample of study participants (n = 523) for whom data on circulating serum fatty acids are available. Although in vivo fatty acids are inherently an imperfect reflection of dietary fat intake, in particular of saturated fat intake^39^, such validation can give an idea of whether our principal exposure variable captures real intake. Details about the assessment of fatty acids in these individuals, who are simultaneously participants in the Cohort of 60-year-olds in Sweden, have been described earlier^40^. Thirty-nine subjects had missing information on the dietary variable.

### Genotyping and quality control in IMPROVE

Illumina Cardiometabo 200 K^41^ and Immunochip 200 K^42^ bead array platforms were used to genotype individual DNA extracted from blood samples. Quality control was performed on the individual chips followed by a further round on the combined chips. During quality control we excluded single nucleotide polymorphisms (SNPs) with low call rate, (< 95%), low minor frequency allele (< 0.05) and deviation from Hardy–Weinberg equilibrium (p < 1 × 10^–6^). We also excluded participants with low call rates (< 95%), relatedness, discrepancy between sex chromosome and registered sex. Multi-dimensional scaling was performed (using default settings in PLINK^43^ to explore population stratification. Outliers were visually identified and excluded. Hence, 100,350 SNPs and 3,407 individuals were available for the gene-diet interaction analysis.

### Ultrasonographic measurements

B-mode ultrasound was performed to measure C-IMT at baseline and after 30 months. The protocol and the precision of the measurements have been described elsewhere^37^. The mean and maximum of the average of the values measured in the left and right wall of the whole carotid tree (C-IMT_mean_ and C-IMT_max_) and in the specific segments including common carotid artert (CC_mean_ and CC_max_), bifurcations (Bif_mean_ and Bif_max_) and internal carotid artery (ICA_mean_ and ICA_max_) were available. Based on recommendation by The American Society of Echography (ASE) Task force, a binary variable with a cut-point ≥ 75th percentile was created for each C-IMT baseline measurement to identify those subjects with abnormal C-IMT^44^. To assess atherosclerotic progression, the measures at 30 months after the baseline were compared to baseline results and change in C-IMT over time (mm/year) was calculated^38^. A binary variable with a cut-point ≥ 75th percentile was created for each C-IMT progression measurement. Any positive change (above zero) was considered a cut-point to identify those who had progressed over the 30-month follow-up period. The reference category included those with no change or who instead had atherosclerotic regression. We excluded participants with missing information on C-IMT progression (n = 307) and those who had a CVD event over the follow-up (n = 193).

### Statistical methods

For descriptive purpose, continuous variables were summarized as median and IQ range and categorical variables were presented as percentages.

For the validation of the dietary binary variable “high intake of food rich in saturated fat”, Spearman partial correlation analyses were employed to assess its correlations with circulating saturated fatty acids (C14:0, C15:0, C16:0 and C18:0), monounsaturated fatty acids (C16:1 and C18:1) and polyunsaturated fatty acids (n-6: C18:2, C18:3, and C20:4; n-3: C18:3, C20:4 and C20:5). Correlation coefficients (r) were considered significant for p values < 0.05.

Logistic regression models and 95% confidence intervals (CI) were employed to evaluate the associations between high dietary saturated fat intake and abnormal C-IMT at baseline and progression over 30-month follow-up. Models were adjusted for sex and age (Model 1) and levels of physical activity, education, alcohol consumption, smoking and MDS 1–3 (Model 2). In addition, sex-stratified analyses were performed.

Gene-diet interaction analysis in relation to abnormal C-IMT were computed using the GEIRA program designed to screen for genome-wide gene-environment interactions^45^. Analyses were performed under the assumption of a dominant genetic model (one or two copies of the minor allele were combined to form the risk variant). Odds ratios (OR) with 95% CI of abnormal CIMT_max_ at baseline were estimated for the following groups: 1) double exposed (high intake of food rich in saturated fat while having risk variant), 2) exposed to high intake of food rich in saturated fat while not having the risk variant and 3) exposed to having the risk variant but not to high intake of food rich in saturated fat. The reference category was the group with neither of the two exposures. Adjustments were made for sex, age, physical activity, education, alcohol consumption, smoking and MDS 1–3. Among the C-IMT measures available, C-IMT_max_ was chosen, for reproducibility reason, since it is commonly used in other studies. Moreover, C-IMT_max_ has been shown to predict CVD events in this population^46^. Interactions were tested on the additive scale and thus defined as departure from additivity of effects^45,47^. The aforementioned ORs were used to derive the Relative Excess Risk due to interaction (RERI)^45^. The 95% CI for this measure was computed using Hosmer and Lemeshow^48^. P-values of < 5 × 10^–7^ for RERI were considered significant after Bonferroni correction (0.05 alpha test/100,350 SNPs). We also tested for multiplicative interactions using the cross-product of each SNP and the dietary variable (p < 5 × 10^–7^ after Bonferroni correction).

Analyses were performed using PLINK 1.7 (quality control analysis), SAS 9.4 (SAS institute, Cary NC, USA; gene-diet interaction analysis) and STATA 12.1 (Corp, College Station, TX, USA; remaining analysis) statistical software.

## Results

Baseline characteristics among the 3,407 participants of the IMPROVE by their intake of food rich in saturated fats are presented in Table 1. High intake of food rich in saturated fats was more common among the participants recruited from the northern regions including Groningen, Stockholm and Kuopio. Compared to subjects with low intake of food rich in saturated fats, those with high intake were more likely be smokers, with low education, physically inactive, hypertensive and diabetic. They also had higher mean values of BMI and CRP but were less likely to have hypercholesterolemia (Table 1). Baseline characteristics differed between men and women: men were more likely to have high intake of food rich in saturated fat and to have diabetes whereas women were more likely to have hypercholesterolemia and hypertension. Further, men consumed more alcohol than women, whereas women were less physically active and had a lower level of education than men. (Suppl Material, Table 1).

**Table 1:** Baseline characteristics of the participants included in the IMPROVE study by intake of food rich in saturated fats.

Variables	Low intake of food rich in saturated fats	High intake of food rich in saturated fats	p value**
All (n)	1,712	1,695	
Women^(%)^	55	48	0.000
Age (y)*	64.3 (59.7;67.2)	64.8 (59.7;67.2)	0.69
**Recruitment center**^**(%; n)**^			0.000
Southern regions			
Perugia	15; 254	15; 246	
Milan	23; 391	8; 136	
Paris^m11^	18; 308	9; 145	
Northern regions			
Groningen^m27^	4; 64	23; 386	
Stockholm	13; 229	18; 299	
Kuopio^m1^	27; 466	29; 483	
**Physical activity**^**(%) m9**^			0.051
Low	19	20	
Moderate	44	46	
High	37	33	
Ever smoker^(%)^	12	17	0.000
**Education**^**(%) m43**^			0.000
≤ 9 y	44	48	
9–12 y	23	28	
> 12 y	33	24	
**Alcohol consumption**^**(%) m19**^			0.163
0 g/day	47	44	
> 0– ≤ 10 g/day	16	17	
> 10–30 g/day	20	23	
> 30 g/day	17	17	
Hypertension^(%)^	69	75	0.000
Hypercholesterolemia^(%) m4^	80	60	0.000
S-Total cholesterol (mmol/L)^m16^*	5.5 (4.8;6.3)	5.4 (4.6;6.2)	0.005
S-High-Density Lipoprotein (mmol/L)^m16^*	1.23 (1.04;1.50)	1.18 (0.98;1.44)	0.000
S-Low-Density Lipoprotein (mmol/L)^m80a^	3.54 (2.90;4.29)	3.46 (2.76;4.16)	0.003
S-Triglycerides (mmol/L)^m16^*	1.28 (0.92;1.83)	1.35 (0.95;1.97)	0.001
Diabetes^(%)m63^	20	30	0.000
S-Glucose (mmol/L)^m12^*	5.4 (4.9;6.1)	5.6 (5.0;6.6)	0.000
S-C-Reactive Protein (mmol/L)^m11^*	1.64 (0.67;3.36)	2.09 (0.92;3.89)	0.000
Body Mass Index (Kg/m^2^)^m3^*	26.4 (23.8;28.8)	27.3 (24.8;30.0)	0.000

*median and IQ range.

**p values were calculated with Pearson's chi-squared test for discrete variables and Wilcoxon rank-sum test for continuous variables.

S: serum.

m: missing.

Results from the validation analyses showed significant positive correlations between self-reported high intake of food rich in saturated fat and serum concentration of the saturated stearic acid, C18:0 (r 0.13) and the n-6 polyunsaturated fatty acid linoleic acid (r 0.09), whereas significant inverse correlations were observed with total n-3 polyunsaturated fatty acids (r -0.23) including C20:5 (r -0.20) and C22:6 (r -0.25). No other significant correlation was observed.

Associations between intake of food rich in saturated fat and abnormal baseline C-IMT are shown in Table 2. Sex- and age- adjusted odds of abnormal C-IMT_mean_, CC-IMT _mean_ and ICA-IMT_mean_ were higher in the group with high intake of food rich in saturated fat as compared to those with low intake. However, after adjustment for lifestyle factors, education and MDS 1–3, results were no longer significant.

**Table 2:** Odd Ratios (OR) and 95% confidence interval (CI) of abnormal C-IMT for high consumption of food rich in saturated fat (vs. low) in the participants of the IMPROVE study.

	OR (95% CI)	OR (95% CI)
	Model 1 (n = 3,407)	Model 2 (n = 3,364)
**IMT**_**mean**_^**m2**^		
_<75th percentile_	Ref	Ref
≥ _75th percentile_	1.25 (1.06;1.47)	1.15 (0.97;1.37)
**IMT**_**max**_^**m2**^		
_<75th percentile_	Ref	Ref
≥ _75th percentile_	1.17 (1.00;1.37)	1.12 (0.95;1.32)
**CC–IMT**_**mean**_^**m3**^		
_<75th percentile_	Ref	Ref
≥ _75th percentile_	1.22 (1.04;1.44)	1.17 (0.98;1.38)
**Bif–IMT**_**mean**_^**m21**^		
_<75th percentile_	Ref	Ref
≥ _75th percentile_	1.04 (0.89;1.22)	1.03 (0.88;1.22)
**ICA IMT**_**mean**_^**m32**^		
_<75th percentile_	Ref	Ref
≥ _75th percentile_	1.26 (1.07;1.48)	1.18 (1.00;1.40)
**CC–IMT**_**max**_^**m3**^		
_<75th percentile_	Ref	Ref
≥ _75th percentile_	0.97 (0.83;1.14)	0.94 (0.80;1.11)
**Bif–IMT**_**max**_^**m21**^		
_<75th percentile_	Ref	Ref
≥ _75th percentile_	1.10 (0.95;1.29)	1.12 (0.95;1.31)
**ICA–IMT**_**max**_^**m32**^		
_<75th percentile_	Ref	Ref
≥ _75th percentile_	1.10 (0.94;1.29)	1.02 (0.86;1.20)

m: missing.

Model 1: Sex- and age-adjusted.

Model 2: Model 1 plus level of education (categorical), physical activity (categorical), smoking, alcohol consumption (categorical) and population structure (MDS1-3, continuous).

No associations between intake of food rich in saturated fat and C-IMT progression were observed either in Model 1 or 2 (Table 3).

**Table 3:** Odd Ratios (OR) and 95% confidence interval (CI) for high consumption of food rich in saturated fats in relation to IMT progression (vs. no change or regression) in the participants of the IMPROVE study.

	OR (95% CI)	OR (95% CI)
	Model 1 (n = 2,907)	Model 2 (n = 2,864)
**IMT**_**mean**_		
No change or regression	Ref	Ref
Progression	1.10 (0.93;1.30)	1.14 (0.95;1.35)
**IMT**_**max**_		
No change or regression	Ref	Ref
Progression	1.03 (0.88;1.20)	1.06 (0.90;1.24)
**CC–IMT**_**mean**_		
No change or regression	Ref	Ref
progression	0.86 (0.74;1.00)	0.87 (0.75;1.02)
**Bif–IMT**_**mean**_		
No change or regression	Ref	Ref
Progression	1.11 (0.95;1.30)	1.13 (0.96;1.34)
**ICA IMT**_**mean**_		
No change or regression	Ref	Ref
Progression	1.06 (0.90;1.23)	1.06 (0.90;1.24)
**CC–IMT**_**max**_		
No change or regression	Ref	Ref
Progression	0.93 (0.80;1.08)	0.95 (0.82;1.11)
**Bif–IMT**_**max**_		
No change or regression	Ref	Ref
Progression	1.01 (0.86;1.17)	1.02 (0.87;1.20)
**ICA–IMT**_**max**_		
No change or regression	Ref	Ref
Progression	1.08 (0.93;1.25)	1.05 (0.90;1.22)

Participants with missing information on progression (n = 307) and those who suffered a cardiovascular event during the follow-up (n = 193) are excluded.

Model 1: Sex- and age-adjusted.

Model 2: Model 1 plus level of education (categorical), physical activity (categorical), smoking, alcohol consumption (categorical) and population structure (MDS1-3 continuous).

Results from the sex-stratified analyses of the association between intake of food rich in saturated fat and C-IMT, baseline and progression, also did not show any clear associations (data not shown).

Results from the gene-dietary fat interaction analyses are shown in Suppl Material Table 2. Before Bonferroni adjustment, a statistically significant (P-value < 0.05) RERI value was observed for 1,450 of the SNPs tested for interaction with high intake of saturated fat (Suppl Material Table 2). Of those results, 1,449 indicated antagonism and one indicated synergism. After Bonferroni correction, none of these results remained statistically significant (P value for RERI < 0.05 × 10^–5^).

## Discussion

In this European population at high risk of CVD but free of clinical manifestations of CVD, no significant associations were found between a diet rich in saturated fat and abnormal C-IMT, nor progression of C-IMT, after adjustments for sex, age, level of education and lifestyle factors. Moreover, we found no significant evidence of interactions between high intake of saturated fat and genetic variants in relation to carotid atherosclerosis.

### Dietary saturated fat in relation to baseline C-IMT and C-IMT progression

Our result of no association between a diet rich in saturated fat and abnormal baseline C-IMT is in line with results of some previous epidemiological studies^23–25,27^. These studies were relatively small (n < 1100) and performed in Australian women^23^, Finnish men^24,25^ or Spanish men and women^27^. However, our result does not support findings of other previous studies, performed in Canada^16^, the U.S.^17,19,22^ and Korea^15,20^ showing associations with increased atherosclerosis. In general, in most of the previous studies, the exposure assessment was limited to the intake of specific food items rich in saturated fat—dairy products, meat or eggs^15,19,20,22–25,27^—which may not capture the overall intake of saturated fats. However, in the Canadian^16^ and U.S.^17^ studies, the total intake of saturated fat (g/day) in relation to C-IMT was considered. The U.S. study was performed between 1987 and 1989 and included 7,100 women and 5,900 men from the general population, with a mean age of 54; the association with increased C-IMT was found in women only^17^. In the Canadian study, participants (n = 620), aged 55 on average, were without CVD risk factors and the association with increased C-IMT was modest^16^. Interestingly, the studies which agree with our results were mainly performed in Europe, whereas those with opposite results were performed in non-European countries. The discrepancy between our results and those reported from non-European populations may perhaps be explained by different dietary patterns, methods of cooking, source of dietary fat or total amount consumed daily. Micha et al.^49^ showed that the amount and sources of saturated fats may vary by geographical regions with Western European countries consuming less processed meat but more vegetables and whole grain than Northern American countries^49^. In light of our results, it is also noteworthy to consider that our study population has been shown to have a good compliance to a Mediterranean diet in general, although with a north–south gradient^50^, in which the fundamental components such as whole grain and alcohol have been shown to modify the increased risk of CVD associated with high intake of dietary saturated fat^51^.

Our finding of no association between dietary saturated fat and C-IMT progression confirms the results reported from two interventional studies (n < 300), performed in Dutch men and women^18^ and Norwegian elderly men^26^. Similar to our study, these two investigations were carried out in participants with risk factors for CVD over an average of 30 months follow-up for atherosclerosis^18,26^. Considering the limited knowledge concerning the relationship between intake of saturated fat and C-IMT progression, further studies are needed to confirm this null association.

Our findings of no association between intake of saturated fats and carotid atherosclerosis do not agree with the previously suggested detrimental effects from saturated fats on cardio-metabolic risk markers and pathophysiologic pathways involved in the atherosclerotic process^5^. However, recent results from the Prospective Urban Rural Epidemiology (PURE) study showed that replacement of saturated fatty acids with carbohydrate or polyunsaturated fat may have a detrimental effect on blood lipids such as triglycerides and High-Density-lipoprotein (HDL)^52^. In addition, in the PURE study a steep reduction in the intake of saturated fat resulted in only a moderate change of Low-Density-lipoprotein (LDL)^52^. Moreover, in the Dietary Intervention and Vascular Study, the replacement of saturated fatty acids with PUFA had no effect on vascular function^53^.

### Absence of findings of gene-dietary saturated fat interactions

To the best of our knowledge, no previous studies have investigated interactions between genetic variation and dietary fats in relation to abnormal C-IMT using a non-hypothesis driven approach based on genetic data from genome-wide screening. Opposite to what perhaps may be expected, our results suggest that single genetic variants do not influence the impact of dietary saturated fats on atherosclerosis. A potential explanation of our null findings might however be that effects are not large enough to be detectable in our sample. Thus, the possibility of false negative results in our study should be noted.

### Limitations

The main limitation of the present study is the basis for assessment of dietary fat intake. Considering that a simple short self-reported questionnaire was used, misclassification of the saturated fat intake may have occurred. However, the results from our validity analyses lend some support to the assumption that the self-reported intake of saturated fat reflects the actual intake. Of course, it is still possible that such misclassification has occurred to some extent, but it is likely to be non-differential and may have biased the results towards the null. Beyond the potential misreporting, misclassification may also be induced by our analytic method, in which we created a binary variable reflecting intake of saturated fats, possibly oversimplifying the underlying dietary intake of fat. Our assessment of dietary fat intake encompassed no characterization of the saturated fat which is another limitation. Not all saturated fatty acids have been shown to have the same effect on lipoproteins involved in the atherosclerotic process^54^. Hence, in further studies, it would be important to consider dietary saturated fat with a specific focus on the different type of saturated fats in relation to subclinical atherosclerosis.

The presence of unmeasured confounding in this study cannot be ruled out. For instance, adjustment for carbohydrate intake was not possible due to the lack of this information in the dietary questionnaire which mainly captures fat intake, especially saturated fat.

This study was performed in participants with at least 3 traditional CVD risk factors which limits the generalizability of results to the general population. Further, the generalizability may be hampered by heterogeneity among participants in terms of intake of food rich in saturated fats and adherence to a Mediterranean diet. Moreover, study participants may have changed their diet upon being diagnosed with a metabolic condition such as hypertension, hyperlipidemia or diabetes, causing a possible decrease in their intake of saturated fats and thus a saturated fats intake classification in our study that does not reflect a long-term dietary habit. The heterogeneity of our study population may also have hampered the possibility to detect significant interactions; hence our absence of findings should be interpreted with caution.

Finally, it should be noted that C-IMT may be questioned regarding its role as a surrogate marker for subclinical atherosclerosis: it may reflect a combination of vascular aging and the atherosclerotic process^55,56^. However, among the available surrogate markers of subclinical atherosclerosis, C-IMT is one of the most widely used and recognized^57–61^. Further, the uncertainty surrounding the C-IMT measure should be weighed against the benefit obtained, from a pathophysiological research perspective, of studying subclinical atherosclerosis instead of manifest CVD, as the latter outcome may have a larger width of underlying pathophysiological causal mechanisms.

## Conclusions

In this European population with at least three risk factors for CVD but free of CVD at baseline, a diet rich in in saturated fat was not associated with atherosclerosis nor its progression measured after 30 months. Our results suggest that the relationship between a diet rich in saturated fat and subclinical atherosclerosis is not modulated by specific genetic variants. These findings need to be confirmed in further studies.

## Supplementary Information

Supplementary Information
