# Supplementary material for: Intake of food rich in saturated fat in relation to subclinical atherosclerosis and potential modulating effects from single genetic variants
Source: Sci Rep. 2021 Apr 12;11:7866. doi: 10.1038/s41598-021-86324-w (PMC8042105; doi:10.1038/s41598-021-86324-w)
Supplement: Supplementary file 1 — Supplementary Information [file 41598_2021_86324_MOESM1_ESM.docx]

Title: Intake of food rich in saturated fat in relation to subclinical atherosclerosis and potential modulating effects from single genetic variants

Federica Laguzzi^1^, Buamina Maitusong^1;2^, Rona J Strawbridge^3;4;5^, Damiano Baldassarre^6^, Fabrizio Veglia^7^, Steve E. Humphries^8^, Rainer Rauramaa^9;10^, Sudhir Kurl^11^, Andries J. Smit^12^, Philippe Giral^13^, Angela Silveira^14^, Elena Tremoli^7^, Anders Hamsten^14^, Ulf de Faire^1^, Bruna Gigante^4^, Karin Leander^1^ on behalf of the IMPROVE study group.

^1^Unit of Cardiovascular and Nutritional Epidemiology, Institute of Environmental Medicine, Karolinska Institutet, Nobels väg 13, Box 210, 17177 Stockholm, Sweden.

^2^Department of Cardiology, First Affiliated Hospital of Xinjiang Medical University, Urumqi, PR China.

^3^Mental Health and Wellbeing, Institute of Mental Health and Wellbeing, University of Glasgow, Glasgow, UK.

^4^Cardiovascular Medicine Unit, Department of Medicine Solna, Karolinska Institutet, Stockholm, Sweden.

^5^Health Data Research United Kingdom

^6^Department of Medical Biotechnology and Translational Medicine, Università degli Studi di Milano, Italy.

^7^Centro Cardiologico Monzino, IRCCS, Milan, Italy.

^8^Centre for Cardiovascular Genetics, Institute Cardiovascular Science, University College London, UK.

^9^Foundation for Research in Health Exercise and Nutrition, Kuopio Research Institute of Exercise Medicine, Kuopio, Finland.

^10^Department of Clinical Physiology and Nuclear Medicine, Kuopio University Hospital, Kuopio, Finland.

^11^ Institute of Public Health and Clinical Nutrition, University of Eastern Finland, Kuopio, Finland

^12^Department of Medicine, University Medical Center Groningen, Groningen, The Netherlands;

^13^Assistance Publique-Hôpitaux de Paris, Service Endocrinologie-Métabolisme, Groupe Hospitalier Pitié-Salpétrière, Unités de Prévention Cardiovasculaire, Paris, France.

^14^Cardiovascular Medicine Unit, Department of Medicine Solna, Karolinska Institutet and Karolinska Hospital, Stockholm, Sweden.

Members of the IMPROVE study group

[Sirtori CR](https://www-ncbi-nlm-nih-gov.proxy.kib.ki.se/pubmed/?term=Sirtori%20CR)^15^, [Calabresi L](https://www-ncbi-nlm-nih-gov.proxy.kib.ki.se/pubmed/?term=Calabresi%20L)^15^, [Amato M](https://www-ncbi-nlm-nih-gov.proxy.kib.ki.se/pubmed/?term=Amato%20M)^7^, [Frigerio B](https://www-ncbi-nlm-nih-gov.proxy.kib.ki.se/pubmed/?term=Frigerio%20B)^7^, [Ravani A](https://www-ncbi-nlm-nih-gov.proxy.kib.ki.se/pubmed/?term=Ravani%20A)^7^, [Sansaro D](https://www-ncbi-nlm-nih-gov.proxy.kib.ki.se/pubmed/?term=Sansaro%20D)^7^, [Tedesco C](https://www-ncbi-nlm-nih-gov.proxy.kib.ki.se/pubmed/?term=Tedesco%20C)^7^, [Coggi D](https://www-ncbi-nlm-nih-gov.proxy.kib.ki.se/pubmed/?term=Coggi%20D)^7^, Capra N^7^, [Bonomi A](https://www-ncbi-nlm-nih-gov.proxy.kib.ki.se/pubmed/?term=Bonomi%20A)^7^, [Eriksson](https://www-ncbi-nlm-nih-gov.proxy.kib.ki.se/pubmed/?term=Eriksson%20MJ) P^4^, [Cooper J](https://www-ncbi-nlm-nih-gov.proxy.kib.ki.se/pubmed/?term=Cooper%20J)^8^, [Acharya J](https://www-ncbi-nlm-nih-gov.proxy.kib.ki.se/pubmed/?term=Acharya%20J)^8^, Savonen K^9^, [Huttunen K](https://www-ncbi-nlm-nih-gov.proxy.kib.ki.se/pubmed/?term=Huttunen%20K)^9^, [Rauramaa E](https://www-ncbi-nlm-nih-gov.proxy.kib.ki.se/pubmed/?term=Rauramaa%20E)^9^, [Penttila IM](https://www-ncbi-nlm-nih-gov.proxy.kib.ki.se/pubmed/?term=Penttila%20IM)^9^, [Törrönen J](https://www-ncbi-nlm-nih-gov.proxy.kib.ki.se/pubmed/?term=T%C3%B6rr%C3%B6nen%20J)^9^, [van Gessel AI](https://www-ncbi-nlm-nih-gov.proxy.kib.ki.se/pubmed/?term=van%20Gessel%20AI)^12^, [van Roon AM](https://www-ncbi-nlm-nih-gov.proxy.kib.ki.se/pubmed/?term=van%20Roon%20AM)^12^, [Nicolai A](https://www-ncbi-nlm-nih-gov.proxy.kib.ki.se/pubmed/?term=Nicolai%20A)^12^, [Mulder DJ](https://www-ncbi-nlm-nih-gov.proxy.kib.ki.se/pubmed/?term=Mulder%20DJ)^12^, G.H. Smeets^12^, [Kontush A](https://www-ncbi-nlm-nih-gov.proxy.kib.ki.se/pubmed/?term=Kontush%20A)^13^, Carrié A^13^, Gallo A^13^, [Karppi J](https://www-ncbi-nlm-nih-gov.proxy.kib.ki.se/pubmed/?term=Karppi%20J)^11^, [Nurmi T](https://www-ncbi-nlm-nih-gov.proxy.kib.ki.se/pubmed/?term=Nurmi%20T)^11^, [Nyyssönen K](https://www-ncbi-nlm-nih-gov.proxy.kib.ki.se/pubmed/?term=Nyyss%C3%B6nen%20K)^11^, [Tuomainen TP](https://www-ncbi-nlm-nih-gov.proxy.kib.ki.se/pubmed/?term=Tuomainen%20TP)^11^, [Tuomainen J](https://www-ncbi-nlm-nih-gov.proxy.kib.ki.se/pubmed/?term=Tuomainen%20J)^11^, [Kauhanen J](https://www-ncbi-nlm-nih-gov.proxy.kib.ki.se/pubmed/?term=Kauhanen%20J)^11^, [Sennblad B](https://www-ncbi-nlm-nih-gov.proxy.kib.ki.se/pubmed/?term=Sennblad%20B%5BAuthor%5D&cauthor=true&cauthor_uid=31932740)^16^, Pirro M^17^, [Vaudo G](https://www-ncbi-nlm-nih-gov.proxy.kib.ki.se/pubmed/?term=Vaudo%20G)^17^, [Siepi D](https://www-ncbi-nlm-nih-gov.proxy.kib.ki.se/pubmed/?term=Siepi%20D)^17^, [Lupattelli G](https://www-ncbi-nlm-nih-gov.proxy.kib.ki.se/pubmed/?term=Lupattelli%20G)^17^, Mannarino M.R^17^, Bianconi V^17^.

^15^Centro Dislipidemie E. Grossi Paoletti, Ospedale Ca' Granda di Niguarda, Milan, Italy.

^16^National Bioinformatics Infrastructure Sweden, Science for Life Laboratory, Uppsala University, Uppsala, Sweden.

^17^Internal Medicine, Angiology and Arteriosclerosis Diseases, Department of Clinical and Experimental Medicine, University of Perugia, Perugia, Italy.

**Material and methods**

Creation of the binary variable for high (vs. low) intake of saturated fat

We created a “Fatscore” based on self-reported dietary habits. We considered the following items as food rich in saturated fats: eggs, meat, semi-skimmed and whole milk, lard, butter and margarine. One point was assigned for those individuals who reported high consumption (>= the median intake reported by study participants) of food with high content of saturated fat: eggs (>=1 time per week), meat (>=4 times per week) and semi-skimmed or whole milk (>=200 ml per day); otherwise zero. One point was also assigned for lard, butter or margarine reported as the main type of fat consumed (as opposed to olive oil or “other” oils that gave zero points). The consumption of fish for its high content of n-3 polyunsaturated fatty acids generates zero point when the consumption was higher than the median (>=2 times per week); otherwise zero. The individual points were summed up to give each participant a final score. A binary variable “high intake of food rich in saturated fats” vs “low” was formed setting a cut-point at the median (>=3) of the Fatscore.

Definition of covariates

Physical activity was defined as: 1) low (brisk walk, less than ten minutes, less than once a week), 2) medium (brisk walk two-three times a week) or 3) high (brisk walk more than three times a week). Individuals who reported previous diagnosis of hypertension and/or use of antihypertensive drugs were considered to have hypertension. Individuals who reported previous diagnosis of diabetes and/or use of insulin or hypoglycemic drugs were considered to have diabetes. Individuals with LDL-C levels >160 mg/dL or who reported use of lipid lowering drugs were considered to have hypercholesterolemia.

Table 1: Baseline characteristics by intake of food rich in saturated fats. Results are presented for men (n=1,643) and women (n=1,764) included in the IMPROVE study.

| **Variable** | **Low intake of food rich in saturated fats** | **High intake of food rich in saturated fats** | **p value**** |
| --- | --- | --- | --- |
| N |  |  |  |
| Men | 763 | 880 |  |
| Women | 949 | 815 |  |
| Age (y)^a^ |  |  | 0. 0934 |
| Men | 64.3 (59.6;67.1) | 64.6 (59.3;67.2) |  |
| Women | 64.3 (59.7;67.5) | 65.1 (60.1;67.5) |  |
| Recruitment center ^(%, count)^ |  |  | 0.0000 |
| Men |  |  |  |
| southern regions |  |  |  |
| Perugia | 6; 45 | 8; 74 |  |
| Milan | 25; 194 | 7; 62 |  |
| Paris^m8^ | 19; 149 | 9; 79 |  |
| northern regions |  |  |  |
| Groningen ^m13^ | 3; 22 | 23; 199 |  |
| Stockholm | 14; 110 | 19; 164 |  |
| Kuopio ^m1^ | 32; 243 | 34; 302 |  |
| Women |  |  |  |
| southern regions |  |  |  |
| Perugia | 22; 209 | 21; 172 |  |
| Milan | 21; 197 | 9; 74 |  |
| Paris^m3^ | 17; 159 | 8; 66 |  |
| northern regions |  |  |  |
| Groningen^m14^ | 4; 42 | 23; 187 |  |
| Stockholm | 12; 119 | 17, 135 |  |
| Kuopio | 23, 223 | 22; 181 |  |
|  |  |  |  |
| Physical activity ^(%) m9^ |  |  | 0.0000 |
| Men |  |  |  |
| Low | 37 | 44 |  |
| Medium | 23 | 27 |  |
| High | 49 | 29 |  |
| Women |  |  |  |
| Low | 22 | 25 |  |
| Medium | 44 | 48 |  |
| High | 34 | 27 |  |
| Ever smoker ^(%)^ |  |  | 0.0000 |
| Men | 14 | 19 |  |
| Women | 11 | 16 |  |
| Education (study years) ^(%) m43^ |  |  | 0.0000 |
| Men |  |  |  |
| ≤9years | 37 | 44 |  |
| 9−12 years | 23 | 27 |  |
| >12 years | 40 | 29 |  |
| Women |  |  |  |
| ≤9years | 49 | 51 |  |
| 9−12 years | 24 | 29 |  |
| >12 years | 27 | 19 |  |
| Alcohol consumption ^(%) m19^ |  |  | 0.0003 |
| Men |  |  |  |
| 0 g/day | 29 | 30 |  |
| >0-10g/day | 15 | 18 |  |
| >10-30g/day | 26 | 28 |  |
| >30g/day | 30 | 24 |  |
| Women |  |  |  |
| 0 g/day | 61 | 59 |  |
| >0-10g/day | 16 | 16 |  |
| >10-20g/day | 16 | 18 |  |
| >20g/day | 7 | 8 |  |
| Hypertension ^(%)^ |  |  | 0.0000 |
| Men | 66 | 73.5 |  |
| Women | 71 | 77 |  |
| Hypercholesterolemia ^(%) m4^ |  |  | 0.0000 |
| Men | 74 | 54 |  |
| Women | 84 | 67 |  |
| S-Total cholesterol (mmol/L) ^m16^ ^a^ |  |  | 0.0001 |
| Men | 5.3 (4.6;6.0) | 5.2 (4.5;5.9) |  |
| Women | 5.7 (4.9;6.5) | 5.7 (4.8;6.4) |  |
| S-HDL (mmol/L) ^m16^ ^a^ |  |  | 0.0001 |
| Men | 1.12 (0.95;1.33) | 1.08 (0.91;1.28) |  |
| Women | 1.34 (1.12;1.60) | 1.31 (1.10;1.60) |  |
| S-LDL (mmol/L) ^m80^ ^a^ |  |  | 0.0001 |
| Men | 3.45 (2.82;4.12) | 3.33 (2.67;3.96) |  |
| Women | 2.97 (3.64;4.46) | 3.67 (2.89;4.34) |  |
| S-Triglycerides (mmol/L) ^m16 a^ |  |  | 0. 0092 |
| Men | 1.34 (0.97;1.98) | 1.41 (0.97;2.07) |  |
| Women | 1.21 (0.89;1.72) | 1.30 (0.93;1.87) |  |
| Diabetes ^(%)^ ^m63^ |  |  | 0.0000 |
| Men | 23 | 36 |  |
| Women | 17 | 24 |  |
| S/P-Glucose (mmol/L) ^m12 a^ |  |  | 0.0001 |
| Men | 5.6 (5.1;6.3) | 5.9 (5.2;7.0) |  |
| Women | 5.2 (4.8;5.9) | 5.4 (4.9;6.2) |  |
| S-C-Reactive Protein (mmol/L) ^m11^ ^a^ |  |  | 0.0001 |
| Men | 1.45 (0.58;3.05) | 1.88 (0.73;3.39) |  |
| Women | 1.87 (0.75;3.61) | 2.36 (1.10;4.30) |  |
| Body Mass Index (Kg/m^2^) ^m3 a^ |  |  | 0. 0777 |
| Men | 26.7 (24.6;28.6) | 27.4 (25.3:29.7) |  |
| Women | 26.0 (23.3;29.2) | 27.1 (24.2;30.3) |  |

^a^median and IQ range

* p values calculated by ANOVA for discrete variables and Kruskal Wallis test for continuous variables

S: serum

P: plasma

m: missing

Table 2: Significant results from gene-diet (high intake of saturated fat) interaction analyses in relation to carotid atherosclerosis (assessed through C-IMT_max_^*^) before Bonferroni correction. Results are adjusted for sex, age, physical inactivity, smoking, level of education, alcohol consumption, and population structure (MDS^†^ 1-3).

|  | N Observations | | | | | | | |  |  | OR (95% CI) | | | |  |
| --- | --- | --- | --- | --- | --- | --- | --- | --- | --- | --- | --- | --- | --- | --- | --- |
|  | Low saturated fat intake without the risk variant | | Low saturated fat intake with the risk variant | | High saturated fat intake without the risk variant | | High saturated fat intake with the risk variant | |  |  | Reference group: Low saturated fat intake without the risk variant | | | |  |
| SNPs | Controls | Cases | Controls | Cases | Controls | Cases | Controls | Cases | Risk variant | MAF | Low saturated fat intake with the risk variant |  | High saturated fat intake without the risk variant | High saturated fat intake with the risk variant | P RERI |
| chr 1 |  |  |  |  |  |  |  |  |  |  |  |  |  |  |  |
| rs10494366 | 459 | 138 | 620 | 252 | 451 | 164 | 718 | 219 | G | 37 | 1.35(1.05;1.73) |  | 1.22(0.93;1.6) | 1.04(0.81;1.34) | 0.02 |
| rs10745330 | 286 | 85 | 793 | 305 | 266 | 96 | 903 | 287 | T | 50 | 1.38(1.04;1.84) |  | 1.34(0.95;1.9) | 1.14(0.85;1.51) | 0.04 |
| rs10798973 | 450 | 131 | 627 | 259 | 435 | 159 | 732 | 224 | A | 37 | 1.43(1.12;1.84) |  | 1.28(0.97;1.69) | 1.09(0.84;1.4) | 0.01 |
| rs10798975 | 451 | 131 | 628 | 259 | 434 | 159 | 735 | 224 | T | 38 | 1.43(1.12;1.84) |  | 1.29(0.98;1.7) | 1.08(0.84;1.4) | 0.01 |
| rs10864790 | 331 | 87 | 836 | 296 | 287 | 113 | 790 | 277 | A | 49 | 1.41(1.06;1.87) |  | 1.55(1.11;2.17) | 1.34(1.01;1.77) | 0.04 |
| rs10875032 | 317 | 74 | 850 | 309 | 257 | 109 | 822 | 281 | G | 50 | 1.48(1.1;1.98) |  | 1.72(1.21;2.44) | 1.38(1.02;1.85) | 0.02 |
| rs10910077 | 589 | 161 | 580 | 222 | 515 | 193 | 564 | 197 | A | 31 | 1.34(1.05;1.71) |  | 1.34(1.04;1.71) | 1.2(0.94;1.54) | 0.04 |
| rs10914754 | 451 | 131 | 626 | 259 | 435 | 159 | 732 | 223 | C | 37 | 1.44(1.12;1.85) |  | 1.28(0.97;1.69) | 1.08(0.84;1.4) | 0.01 |
| rs10920115 | 288 | 92 | 791 | 298 | 294 | 115 | 875 | 268 | G | 49 | 1.25(0.94;1.65) |  | 1.36(0.98;1.9) | 1.01(0.76;1.34) | 0.03 |
| rs10927013 | 238 | 57 | 931 | 326 | 191 | 78 | 888 | 312 | T | 43 | 1.53(1.1;2.11) |  | 1.76(1.17;2.64) | 1.48(1.07;2.05) | 0.04 |
| rs11102356 | 496 | 151 | 572 | 232 | 498 | 172 | 635 | 202 | G | 33 | 1.3(1.01;1.65) |  | 1.18(0.91;1.53) | 1.02(0.79;1.31) | 0.04 |
| rs11118475 | 269 | 80 | 810 | 310 | 288 | 105 | 881 | 278 | G | 50 | 1.41(1.05;1.89) |  | 1.37(0.96;1.95) | 1.16(0.86;1.56) | 0.04 |
| rs1146359 | 956 | 276 | 213 | 107 | 873 | 317 | 206 | 73 | T | 11 | 1.75(1.33;2.32) |  | 1.24(1.03;1.51) | 1.15(0.84;1.57) | 0.01 |
| rs11584093 | 760 | 222 | 409 | 161 | 678 | 253 | 401 | 137 | C | 21 | 1.32(1.04;1.69) |  | 1.28(1.03;1.58) | 1.09(0.85;1.41) | 0.03 |
| rs11590246 | 162 | 37 | 1006 | 346 | 105 | 56 | 974 | 334 | T | 6 | 1.45(0.98;2.14) |  | 2.18(1.32;3.6) | 1.43(0.97;2.11) | 0.04 |
| rs11590511 | 946 | 282 | 210 | 96 | 847 | 302 | 220 | 76 | G | 11 | 1.55(1.16;2.06) |  | 1.18(0.97;1.43) | 1.13(0.83;1.53) | 0.04 |
| rs11590812 | 423 | 130 | 656 | 260 | 398 | 149 | 771 | 234 | C | 41 | 1.44(1.12;1.86) |  | 1.33(1;1.76) | 1.09(0.84;1.4) | 0.01 |
| rs11806197 | 406 | 112 | 673 | 278 | 405 | 155 | 764 | 228 | G | 20 | 1.52(1.17;1.97) |  | 1.38(1.03;1.84) | 1.14(0.87;1.49) | 0.01 |
| rs11809524 | 799 | 258 | 280 | 132 | 835 | 271 | 334 | 112 | C | 15 | 1.53(1.18;1.98) |  | 1.04(0.85;1.28) | 1.07(0.82;1.39) | 0.04 |
| rs11811702 | 558 | 174 | 611 | 209 | 497 | 203 | 582 | 187 | G | 28 | 1.15(0.9;1.46) |  | 1.33(1.04;1.7) | 1.02(0.8;1.31) | 0.04 |
| rs12036340 | 534 | 156 | 635 | 227 | 467 | 199 | 612 | 191 | G | 26 | 1.19(0.93;1.52) |  | 1.41(1.09;1.81) | 1.03(0.8;1.32) | 0.01 |
| rs12038038 | 586 | 182 | 493 | 208 | 584 | 201 | 585 | 182 | C | 29 | 1.35(1.07;1.72) |  | 1.14(0.9;1.45) | 1.01(0.79;1.29) | 0.03 |
| rs12038793 | 362 | 102 | 807 | 281 | 325 | 136 | 754 | 254 | G | 17 | 1.25(0.96;1.64) |  | 1.48(1.09;2.02) | 1.17(0.9;1.54) | 0.04 |
| rs12041757 | 426 | 130 | 648 | 257 | 431 | 150 | 734 | 233 | A | 39 | 1.3(1.01;1.67) |  | 1.21(0.91;1.6) | 1.04(0.8;1.34) | 0.04 |
| rs12042962 | 343 | 100 | 736 | 290 | 340 | 126 | 829 | 257 | T | 45 | 1.32(1.01;1.72) |  | 1.32(0.97;1.81) | 1.05(0.8;1.38) | 0.03 |
| rs12067413 | 345 | 107 | 823 | 276 | 296 | 136 | 783 | 254 | A | 16 | 1.1(0.84;1.43) |  | 1.49(1.1;2.03) | 1.03(0.79;1.35) | 0.03 |
| rs1208932 | 346 | 82 | 823 | 301 | 341 | 134 | 738 | 256 | T | 45 | 1.52(1.14;2.01) |  | 1.6(1.16;2.21) | 1.41(1.06;1.88) | 0.02 |
| rs12143842 | 619 | 191 | 460 | 199 | 644 | 226 | 525 | 157 | T | 26 | 1.41(1.11;1.79) |  | 1.15(0.91;1.45) | 1.01(0.79;1.29) | 0.01 |
| rs12725602 | 300 | 72 | 869 | 311 | 254 | 99 | 825 | 291 | A | 49 | 1.59(1.18;2.15) |  | 1.75(1.22;2.51) | 1.49(1.1;2.02) | 0.02 |
| rs12729914 | 404 | 111 | 675 | 279 | 404 | 154 | 765 | 229 | C | 20 | 1.52(1.17;1.97) |  | 1.38(1.03;1.85) | 1.15(0.88;1.5) | 0.01 |
| rs12731889 | 545 | 163 | 534 | 227 | 518 | 172 | 651 | 211 | A | 33 | 1.45(1.14;1.84) |  | 1.16(0.9;1.5) | 1.11(0.87;1.41) | 0.03 |
| rs12747565 | 624 | 175 | 545 | 208 | 551 | 205 | 528 | 185 | T | 28 | 1.37(1.08;1.74) |  | 1.33(1.05;1.69) | 1.19(0.93;1.53) | 0.03 |
| rs1338064 | 840 | 270 | 239 | 120 | 863 | 289 | 306 | 94 | C | 14 | 1.66(1.27;2.16) |  | 1.07(0.88;1.3) | 1.03(0.78;1.36) | 0.01 |
| rs1355138 | 398 | 126 | 771 | 257 | 334 | 157 | 745 | 233 | A | 18 | 1.1(0.85;1.42) |  | 1.48(1.11;1.98) | 1(0.77;1.3) | 0.02 |
| rs1359552 | 534 | 157 | 635 | 226 | 437 | 173 | 642 | 217 | T | 34 | 1.24(0.97;1.58) |  | 1.36(1.05;1.76) | 1.13(0.88;1.44) | 0.04 |
| rs1361754 | 309 | 68 | 860 | 315 | 216 | 101 | 863 | 289 | G | 48 | 1.6(1.18;2.16) |  | 1.97(1.37;2.84) | 1.46(1.07;1.97) | 0.01 |
| rs1387815 | 991 | 304 | 178 | 79 | 855 | 317 | 224 | 73 | T | 42 | 1.58(1.17;2.15) |  | 1.19(0.98;1.44) | 1.1(0.82;1.5) | 0.03 |
| rs1415259 | 459 | 138 | 620 | 252 | 451 | 164 | 718 | 219 | C | 37 | 1.35(1.05;1.73) |  | 1.22(0.93;1.6) | 1.04(0.81;1.34) | 0.02 |
| rs1498243 | 347 | 107 | 822 | 276 | 295 | 136 | 784 | 254 | A | 16 | 1.11(0.85;1.45) |  | 1.51(1.11;2.05) | 1.04(0.79;1.35) | 0.03 |
| rs1507262 | 463 | 131 | 706 | 252 | 371 | 161 | 708 | 229 | T | 39 | 1.24(0.97;1.6) |  | 1.52(1.15;2.01) | 1.09(0.85;1.41) | 0.01 |
| rs1507263 | 490 | 140 | 679 | 243 | 408 | 172 | 671 | 218 | C | 37 | 1.25(0.98;1.6) |  | 1.48(1.13;1.94) | 1.09(0.85;1.4) | 0.01 |
| rs1507270 | 772 | 233 | 396 | 150 | 673 | 257 | 404 | 133 | G | 20 | 1.28(1;1.64) |  | 1.25(1.01;1.55) | 1.06(0.82;1.37) | 0.03 |
| rs1507277 | 461 | 130 | 708 | 253 | 371 | 162 | 708 | 228 | C | 39 | 1.25(0.97;1.6) |  | 1.54(1.17;2.04) | 1.09(0.85;1.4) | 0.01 |
| rs1507286 | 446 | 129 | 723 | 254 | 358 | 153 | 721 | 237 | G | 40 | 1.21(0.94;1.56) |  | 1.48(1.11;1.96) | 1.09(0.85;1.41) | 0.02 |
| rs1577504 | 292 | 68 | 877 | 315 | 240 | 95 | 838 | 295 | A | 48 | 1.59(1.17;2.15) |  | 1.66(1.15;2.4) | 1.52(1.12;2.07) | 0.04 |
| rs17035363 | 1022 | 299 | 147 | 84 | 923 | 328 | 156 | 62 | A | 8 | 1.93(1.42;2.64) |  | 1.19(0.99;1.44) | 1.29(0.92;1.8) | 0.02 |
| rs17127247 | 955 | 279 | 214 | 104 | 831 | 299 | 248 | 91 | C | 12 | 1.63(1.23;2.16) |  | 1.2(0.98;1.46) | 1.23(0.93;1.64) | 0.04 |
| rs17504775 | 883 | 251 | 286 | 132 | 795 | 278 | 284 | 112 | A | 15 | 1.68(1.29;2.17) |  | 1.24(1.01;1.52) | 1.32(1;1.72) | 0.03 |
| rs1773349 | 301 | 83 | 868 | 300 | 263 | 112 | 816 | 278 | C | 50 | 1.32(0.99;1.76) |  | 1.57(1.12;2.22) | 1.26(0.94;1.69) | 0.04 |
| rs1880670 | 646 | 184 | 523 | 199 | 543 | 218 | 536 | 172 | C | 27 | 1.4(1.1;1.78) |  | 1.44(1.14;1.83) | 1.1(0.86;1.41) | 0.00 |
| rs1932351 | 289 | 64 | 877 | 314 | 239 | 95 | 839 | 295 | C | 48 | 1.67(1.22;2.28) |  | 1.76(1.21;2.56) | 1.6(1.17;2.19) | 0.03 |
| rs1932933 | 447 | 139 | 632 | 251 | 422 | 153 | 747 | 230 | T | 38 | 1.29(1;1.65) |  | 1.19(0.9;1.57) | 1.02(0.79;1.31) | 0.04 |
| rs1999999 | 353 | 92 | 816 | 291 | 279 | 124 | 800 | 266 | T | 15 | 1.35(1.03;1.78) |  | 1.64(1.18;2.27) | 1.25(0.94;1.65) | 0.02 |
| rs2039839 | 236 | 56 | 933 | 327 | 188 | 77 | 891 | 313 | G | 42 | 1.54(1.11;2.14) |  | 1.79(1.19;2.68) | 1.49(1.07;2.08) | 0.04 |
| rs2056387 | 130 | 26 | 949 | 364 | 139 | 55 | 1030 | 328 | C | 33 | 2.02(1.29;3.18) |  | 2.15(1.25;3.69) | 1.71(1.09;2.69) | 0.03 |
| rs2246175 | 530 | 167 | 549 | 223 | 507 | 177 | 661 | 206 | T | 33 | 1.36(1.07;1.73) |  | 1.16(0.9;1.5) | 1.04(0.81;1.33) | 0.03 |
| rs228654 | 234 | 63 | 935 | 320 | 207 | 95 | 872 | 295 | A | 11 | 1.24(0.9;1.7) |  | 1.72(1.17;2.52) | 1.18(0.86;1.63) | 0.03 |
| rs2479408 | 801 | 236 | 368 | 147 | 689 | 253 | 390 | 137 | G | 19 | 1.41(1.1;1.81) |  | 1.24(1;1.54) | 1.17(0.91;1.5) | 0.04 |
| rs2488433 | 1037 | 314 | 132 | 69 | 946 | 341 | 133 | 49 | A | 7 | 1.72(1.23;2.39) |  | 1.17(0.97;1.4) | 1.15(0.8;1.66) | 0.04 |
| rs2576221 | 488 | 132 | 681 | 251 | 417 | 159 | 662 | 231 | C | 37 | 1.43(1.11;1.83) |  | 1.43(1.09;1.88) | 1.29(1;1.66) | 0.03 |
| rs2576222 | 489 | 132 | 680 | 251 | 417 | 159 | 662 | 231 | T | 37 | 1.43(1.12;1.84) |  | 1.43(1.09;1.89) | 1.29(1;1.67) | 0.03 |
| rs267681 | 459 | 133 | 620 | 257 | 446 | 163 | 723 | 220 | T | 37 | 1.44(1.12;1.85) |  | 1.28(0.98;1.69) | 1.08(0.84;1.4) | 0.01 |
| rs273885 | 313 | 74 | 855 | 309 | 257 | 108 | 821 | 282 | C | 50 | 1.45(1.08;1.95) |  | 1.68(1.18;2.38) | 1.37(1.01;1.84) | 0.02 |
| rs2744741 | 504 | 130 | 665 | 252 | 418 | 153 | 661 | 237 | T | 37 | 1.5(1.17;1.92) |  | 1.42(1.07;1.87) | 1.36(1.06;1.75) | 0.03 |
| rs2765267 | 534 | 144 | 635 | 239 | 450 | 175 | 629 | 215 | A | 35 | 1.44(1.12;1.84) |  | 1.5(1.15;1.95) | 1.22(0.95;1.57) | 0.01 |
| rs2807339 | 555 | 164 | 614 | 219 | 490 | 200 | 589 | 190 | T | 28 | 1.2(0.95;1.53) |  | 1.37(1.07;1.75) | 1.05(0.82;1.35) | 0.02 |
| rs2880605 | 689 | 212 | 480 | 171 | 615 | 244 | 464 | 145 | T | 36 | 1.22(0.95;1.55) |  | 1.27(1.02;1.59) | 1.03(0.8;1.33) | 0.04 |
| rs3754353 | 257 | 70 | 912 | 313 | 200 | 95 | 879 | 295 | G | 11 | 1.18(0.88;1.6) |  | 1.67(1.15;2.42) | 1.14(0.84;1.54) | 0.03 |
| rs3762409 | 316 | 74 | 853 | 309 | 256 | 108 | 823 | 282 | G | 50 | 1.47(1.09;1.97) |  | 1.7(1.2;2.42) | 1.37(1.02;1.85) | 0.02 |
| rs3820667 | 356 | 85 | 812 | 298 | 333 | 131 | 746 | 259 | A | 45 | 1.53(1.16;2.02) |  | 1.65(1.19;2.27) | 1.4(1.05;1.86) | 0.01 |
| rs3849268 | 267 | 80 | 812 | 310 | 287 | 104 | 882 | 279 | A | 50 | 1.4(1.04;1.87) |  | 1.35(0.95;1.92) | 1.15(0.86;1.55) | 0.04 |
| rs409226 | 479 | 145 | 600 | 245 | 472 | 168 | 697 | 215 | A | 35 | 1.35(1.05;1.72) |  | 1.19(0.91;1.55) | 1.05(0.82;1.34) | 0.03 |
| rs464218 | 365 | 86 | 803 | 297 | 339 | 134 | 740 | 256 | G | 44 | 1.56(1.18;2.07) |  | 1.68(1.22;2.31) | 1.41(1.06;1.87) | 0.01 |
| rs4645983 | 665 | 202 | 446 | 163 | 579 | 221 | 456 | 146 | A | 24 | 1.23(0.96;1.58) |  | 1.25(0.99;1.57) | 1.02(0.79;1.32) | 0.04 |
| rs4654821 | 510 | 148 | 569 | 242 | 472 | 160 | 697 | 223 | T | 35 | 1.52(1.19;1.94) |  | 1.23(0.94;1.61) | 1.14(0.89;1.46) | 0.01 |
| rs4847326 | 574 | 185 | 505 | 205 | 594 | 206 | 575 | 177 | C | 28 | 1.35(1.06;1.71) |  | 1.14(0.89;1.44) | 1.01(0.79;1.29) | 0.03 |
| rs4908165 | 199 | 55 | 880 | 335 | 180 | 70 | 989 | 313 | C | 41 | 1.47(1.05;2.06) |  | 1.54(1;2.35) | 1.23(0.88;1.73) | 0.04 |
| rs4915593 | 603 | 179 | 566 | 204 | 511 | 208 | 568 | 182 | C | 29 | 1.2(0.94;1.53) |  | 1.34(1.06;1.71) | 1.04(0.82;1.33) | 0.02 |
| rs4949927 | 623 | 197 | 456 | 193 | 664 | 231 | 505 | 152 | C | 25 | 1.39(1.09;1.77) |  | 1.14(0.9;1.43) | 1(0.78;1.29) | 0.02 |
| rs516084 | 239 | 53 | 930 | 330 | 199 | 84 | 880 | 306 | G | 44 | 1.57(1.13;2.19) |  | 1.88(1.25;2.81) | 1.5(1.07;2.1) | 0.02 |
| rs536912 | 725 | 197 | 444 | 186 | 635 | 234 | 443 | 156 | C | 23 | 1.47(1.16;1.88) |  | 1.31(1.05;1.64) | 1.23(0.96;1.58) | 0.02 |
| rs550743 | 713 | 192 | 456 | 191 | 627 | 231 | 452 | 159 | T | 23 | 1.48(1.16;1.88) |  | 1.32(1.05;1.66) | 1.24(0.96;1.59) | 0.02 |
| rs642773 | 780 | 225 | 389 | 158 | 675 | 256 | 404 | 134 | T | 20 | 1.37(1.07;1.76) |  | 1.27(1.03;1.58) | 1.12(0.87;1.44) | 0.02 |
| rs6537671 | 655 | 192 | 514 | 191 | 582 | 223 | 497 | 167 | C | 26 | 1.29(1.01;1.64) |  | 1.31(1.04;1.66) | 1.11(0.86;1.42) | 0.03 |
| rs6537741 | 434 | 130 | 735 | 253 | 368 | 167 | 711 | 223 | T | 20 | 1.15(0.9;1.48) |  | 1.48(1.12;1.96) | 1.03(0.8;1.33) | 0.02 |
| rs6593669 | 745 | 232 | 321 | 148 | 770 | 257 | 376 | 119 | C | 18 | 1.47(1.14;1.89) |  | 1.11(0.9;1.37) | 1.01(0.78;1.32) | 0.02 |
| rs6665139 | 427 | 129 | 742 | 254 | 354 | 154 | 725 | 236 | A | 19 | 1.15(0.9;1.48) |  | 1.46(1.1;1.94) | 1.05(0.81;1.36) | 0.02 |
| rs6665426 | 668 | 194 | 501 | 189 | 576 | 223 | 503 | 167 | A | 26 | 1.39(1.09;1.77) |  | 1.34(1.07;1.69) | 1.15(0.9;1.48) | 0.01 |
| rs6665621 | 460 | 128 | 709 | 255 | 383 | 157 | 696 | 233 | G | 39 | 1.28(0.99;1.64) |  | 1.42(1.07;1.88) | 1.18(0.91;1.52) | 0.04 |
| rs6670064 | 266 | 79 | 813 | 311 | 259 | 99 | 910 | 284 | C | 48 | 1.42(1.06;1.9) |  | 1.39(0.97;1.98) | 1.16(0.86;1.56) | 0.03 |
| rs6670339 | 469 | 139 | 610 | 251 | 455 | 162 | 714 | 221 | C | 36 | 1.37(1.07;1.76) |  | 1.22(0.93;1.59) | 1.06(0.83;1.37) | 0.02 |
| rs6673531 | 321 | 74 | 847 | 309 | 250 | 107 | 829 | 283 | G | 50 | 1.51(1.13;2.03) |  | 1.77(1.24;2.51) | 1.4(1.04;1.88) | 0.01 |
| rs6677347 | 1033 | 314 | 136 | 69 | 942 | 343 | 137 | 47 | T | 7 | 1.7(1.22;2.36) |  | 1.17(0.98;1.41) | 1.11(0.77;1.6) | 0.03 |
| rs6686605 | 690 | 206 | 478 | 177 | 613 | 235 | 466 | 155 | C | 24 | 1.23(0.96;1.56) |  | 1.27(1.02;1.6) | 1.06(0.82;1.35) | 0.04 |
| rs6694545 | 479 | 143 | 690 | 240 | 422 | 180 | 657 | 210 | A | 23 | 1.11(0.87;1.42) |  | 1.36(1.05;1.78) | 1.02(0.79;1.31) | 0.04 |
| rs6701127 | 288 | 77 | 790 | 313 | 283 | 113 | 883 | 270 | G | 49 | 1.46(1.09;1.95) |  | 1.52(1.08;2.15) | 1.15(0.85;1.54) | 0.01 |
| rs6703335 | 239 | 56 | 930 | 327 | 188 | 78 | 891 | 312 | G | 43 | 1.57(1.13;2.17) |  | 1.83(1.22;2.75) | 1.51(1.08;2.09) | 0.03 |
| rs699755 | 1035 | 306 | 134 | 77 | 941 | 337 | 138 | 53 | T | 7 | 1.94(1.41;2.67) |  | 1.19(0.99;1.43) | 1.22(0.86;1.75) | 0.02 |
| rs699766 | 1036 | 309 | 133 | 74 | 943 | 337 | 136 | 53 | A | 7 | 1.85(1.33;2.56) |  | 1.18(0.98;1.42) | 1.23(0.86;1.75) | 0.03 |
| rs716288 | 677 | 200 | 492 | 183 | 612 | 240 | 467 | 150 | G | 35 | 1.28(1.01;1.63) |  | 1.33(1.06;1.67) | 1.04(0.81;1.34) | 0.01 |
| rs7413698 | 277 | 75 | 802 | 315 | 276 | 100 | 892 | 283 | A | 49 | 1.45(1.08;1.94) |  | 1.38(0.97;1.97) | 1.19(0.89;1.6) | 0.04 |
| rs7513120 | 264 | 80 | 815 | 310 | 238 | 99 | 931 | 284 | G | 47 | 1.33(1;1.78) |  | 1.43(1;2.05) | 1.08(0.81;1.45) | 0.03 |
| rs7519954 | 700 | 209 | 469 | 174 | 586 | 223 | 493 | 167 | A | 25 | 1.34(1.05;1.71) |  | 1.31(1.04;1.64) | 1.13(0.88;1.44) | 0.02 |
| rs7540019 | 307 | 90 | 772 | 300 | 278 | 104 | 889 | 279 | A | 50 | 1.31(1;1.74) |  | 1.35(0.96;1.89) | 1.07(0.81;1.42) | 0.04 |
| rs7541691 | 451 | 131 | 628 | 259 | 434 | 158 | 735 | 225 | C | 38 | 1.43(1.12;1.84) |  | 1.28(0.97;1.69) | 1.09(0.84;1.4) | 0.01 |
| rs7550692 | 498 | 150 | 580 | 240 | 489 | 173 | 679 | 209 | T | 34 | 1.37(1.07;1.75) |  | 1.19(0.91;1.54) | 1.05(0.82;1.35) | 0.03 |
| rs7552867 | 810 | 243 | 359 | 140 | 738 | 277 | 341 | 113 | C | 17 | 1.36(1.06;1.76) |  | 1.25(1.01;1.53) | 1.09(0.84;1.43) | 0.03 |
| rs7554097 | 317 | 98 | 762 | 292 | 331 | 124 | 838 | 259 | C | 47 | 1.27(0.97;1.67) |  | 1.3(0.95;1.79) | 1.03(0.78;1.35) | 0.04 |
| rs7556416 | 462 | 128 | 707 | 255 | 384 | 156 | 695 | 234 | A | 39 | 1.29(1;1.66) |  | 1.41(1.07;1.87) | 1.19(0.92;1.54) | 0.04 |
| rs823082 | 309 | 68 | 860 | 315 | 216 | 101 | 863 | 289 | C | 48 | 1.6(1.18;2.16) |  | 1.97(1.37;2.84) | 1.46(1.07;1.97) | 0.01 |
| rs875294 | 382 | 110 | 697 | 280 | 382 | 138 | 786 | 245 | G | 42 | 1.44(1.11;1.86) |  | 1.3(0.97;1.76) | 1.13(0.87;1.47) | 0.02 |
| rs877041 | 440 | 118 | 729 | 265 | 402 | 152 | 677 | 237 | A | 38 | 1.46(1.13;1.89) |  | 1.44(1.08;1.92) | 1.34(1.03;1.74) | 0.04 |
| rs9324162 | 399 | 110 | 680 | 280 | 403 | 152 | 766 | 231 | G | 20 | 1.51(1.16;1.96) |  | 1.36(1.01;1.82) | 1.15(0.88;1.5) | 0.01 |
| rs9332579 | 252 | 80 | 827 | 310 | 249 | 100 | 920 | 283 | T | 12 | 1.23(0.92;1.65) |  | 1.37(0.96;1.96) | 1.02(0.76;1.36) | 0.04 |
| rs9428100 | 691 | 206 | 478 | 177 | 629 | 242 | 450 | 148 | C | 24 | 1.31(1.03;1.66) |  | 1.31(1.05;1.64) | 1.07(0.83;1.38) | 0.02 |
| rs947420 | 175 | 36 | 994 | 347 | 127 | 51 | 952 | 339 | C | 35 | 1.88(1.26;2.81) |  | 2.17(1.31;3.6) | 1.85(1.24;2.76) | 0.04 |
| rs950455 | 555 | 153 | 614 | 230 | 473 | 179 | 605 | 210 | A | 33 | 1.36(1.07;1.74) |  | 1.38(1.06;1.78) | 1.21(0.94;1.55) | 0.03 |
| Chr2 |  |  |  |  |  |  |  |  |  |  |  |  |  |  |  |
| rs10182508 | 990 | 307 | 179 | 76 | 865 | 319 | 214 | 71 | G | 9 | 1.45(1.07;1.97) |  | 1.19(0.98;1.43) | 1.01(0.74;1.38) | 0.03 |
| rs10193976 | 712 | 223 | 367 | 167 | 752 | 249 | 417 | 134 | C | 20 | 1.47(1.15;1.87) |  | 1.09(0.88;1.35) | 1.04(0.81;1.35) | 0.03 |
| rs10200472 | 201 | 62 | 878 | 328 | 170 | 78 | 999 | 305 | T | 9 | 1.22(0.88;1.68) |  | 1.55(1.04;2.33) | 1.01(0.73;1.4) | 0.03 |
| rs1046974 | 175 | 48 | 904 | 342 | 176 | 76 | 993 | 307 | G | 40 | 1.31(0.92;1.86) |  | 1.68(1.09;2.58) | 1.09(0.76;1.55) | 0.03 |
| rs10495809 | 180 | 47 | 989 | 336 | 129 | 62 | 950 | 328 | G | 36 | 1.35(0.95;1.93) |  | 2(1.26;3.15) | 1.32(0.92;1.88) | 0.03 |
| rs10496171 | 669 | 192 | 500 | 191 | 612 | 235 | 467 | 155 | T | 25 | 1.31(1.03;1.66) |  | 1.34(1.07;1.68) | 1.07(0.83;1.38) | 0.01 |
| rs10496882 | 321 | 96 | 758 | 294 | 297 | 111 | 872 | 272 | T | 48 | 1.33(1.02;1.75) |  | 1.33(0.96;1.84) | 1.08(0.82;1.42) | 0.03 |
| rs10779894 | 411 | 105 | 758 | 278 | 365 | 143 | 714 | 246 | C | 42 | 1.44(1.1;1.87) |  | 1.52(1.13;2.05) | 1.31(1;1.71) | 0.02 |
| rs11126666 | 555 | 177 | 524 | 213 | 600 | 209 | 569 | 174 | A | 30 | 1.31(1.03;1.67) |  | 1.13(0.88;1.43) | 1(0.78;1.29) | 0.04 |
| rs1167437 | 145 | 28 | 1024 | 355 | 87 | 49 | 992 | 341 | C | 31 | 1.78(1.15;2.74) |  | 3(1.73;5.22) | 1.72(1.11;2.65) | 0.02 |
| rs11677146 | 489 | 127 | 680 | 256 | 418 | 163 | 660 | 227 | C | 38 | 1.46(1.13;1.87) |  | 1.49(1.13;1.96) | 1.29(1;1.67) | 0.01 |
| rs11682412 | 545 | 170 | 534 | 220 | 551 | 190 | 618 | 193 | C | 31 | 1.33(1.05;1.7) |  | 1.17(0.91;1.5) | 1(0.79;1.28) | 0.02 |
| rs11688831 | 459 | 146 | 620 | 244 | 468 | 162 | 701 | 221 | A | 37 | 1.29(1.01;1.65) |  | 1.18(0.91;1.54) | 1.01(0.79;1.29) | 0.04 |
| rs11690137 | 443 | 134 | 722 | 249 | 338 | 151 | 739 | 237 | G | 20 | 1.16(0.9;1.49) |  | 1.48(1.12;1.97) | 1.04(0.81;1.34) | 0.02 |
| rs1183799 | 190 | 39 | 979 | 344 | 139 | 72 | 940 | 318 | G | 37 | 1.85(1.26;2.7) |  | 2.73(1.71;4.34) | 1.72(1.17;2.51) | 0.01 |
| rs11888231 | 746 | 222 | 423 | 161 | 686 | 264 | 393 | 126 | T | 21 | 1.37(1.07;1.75) |  | 1.3(1.05;1.62) | 1.08(0.83;1.4) | 0.01 |
| rs11892719 | 328 | 82 | 840 | 301 | 274 | 113 | 805 | 277 | T | 48 | 1.48(1.12;1.97) |  | 1.63(1.16;2.29) | 1.39(1.04;1.85) | 0.02 |
| rs1191686 | 791 | 239 | 362 | 142 | 698 | 265 | 375 | 118 | T | 18 | 1.41(1.09;1.81) |  | 1.28(1.04;1.58) | 1.03(0.79;1.34) | 0.01 |
| rs12466789 | 187 | 42 | 982 | 341 | 145 | 60 | 934 | 330 | G | 37 | 1.62(1.11;2.34) |  | 2.03(1.27;3.24) | 1.58(1.09;2.29) | 0.04 |
| rs12469063 | 527 | 153 | 642 | 230 | 445 | 183 | 634 | 207 | G | 25 | 1.26(0.99;1.6) |  | 1.4(1.08;1.81) | 1.11(0.87;1.43) | 0.02 |
| rs12612780 | 114 | 18 | 964 | 371 | 102 | 38 | 1067 | 345 | A | 30 | 2.47(1.46;4.16) |  | 2.66(1.41;5.04) | 2.1(1.24;3.54) | 0.04 |
| rs12614410 | 211 | 58 | 868 | 332 | 217 | 87 | 952 | 296 | T | 10 | 1.4(1.01;1.93) |  | 1.48(1;2.2) | 1.16(0.83;1.61) | 0.04 |
| rs12710675 | 764 | 231 | 405 | 152 | 648 | 258 | 431 | 132 | A | 39 | 1.29(1.01;1.65) |  | 1.28(1.03;1.58) | 1.04(0.81;1.34) | 0.02 |
| rs13021001 | 336 | 98 | 833 | 285 | 259 | 117 | 820 | 273 | G | 14 | 1.11(0.85;1.45) |  | 1.56(1.13;2.15) | 1.04(0.8;1.37) | 0.03 |
| rs13025020 | 352 | 91 | 817 | 292 | 285 | 117 | 794 | 273 | G | 47 | 1.42(1.07;1.87) |  | 1.57(1.13;2.18) | 1.33(1;1.76) | 0.03 |
| rs13034681 | 697 | 218 | 382 | 172 | 699 | 232 | 469 | 151 | C | 22 | 1.39(1.09;1.78) |  | 1.09(0.88;1.36) | 1(0.78;1.29) | 0.03 |
| rs1309 | 303 | 77 | 866 | 306 | 233 | 101 | 846 | 289 | G | 48 | 1.4(1.04;1.88) |  | 1.69(1.19;2.42) | 1.32(0.98;1.77) | 0.02 |
| rs13396575 | 928 | 281 | 241 | 102 | 831 | 307 | 248 | 83 | C | 12 | 1.45(1.1;1.91) |  | 1.2(0.99;1.46) | 1.09(0.81;1.45) | 0.03 |
| rs13415496 | 157 | 43 | 922 | 347 | 146 | 69 | 1023 | 314 | G | 7 | 1.56(1.07;2.28) |  | 2.06(1.3;3.27) | 1.27(0.87;1.85) | 0.01 |
| rs13415618 | 210 | 62 | 869 | 328 | 203 | 87 | 966 | 296 | A | 10 | 1.29(0.94;1.78) |  | 1.48(1;2.19) | 1.08(0.78;1.48) | 0.04 |
| rs1396836 | 628 | 187 | 541 | 196 | 586 | 232 | 493 | 158 | T | 27 | 1.31(1.03;1.67) |  | 1.36(1.08;1.71) | 1.08(0.84;1.39) | 0.01 |
| rs1427539 | 508 | 143 | 658 | 238 | 411 | 162 | 668 | 228 | C | 37 | 1.38(1.08;1.77) |  | 1.46(1.11;1.92) | 1.23(0.95;1.57) | 0.02 |
| rs1486236 | 478 | 127 | 691 | 256 | 410 | 163 | 668 | 227 | A | 38 | 1.39(1.08;1.79) |  | 1.5(1.14;1.98) | 1.22(0.95;1.58) | 0.01 |
| rs1518739 | 362 | 98 | 807 | 285 | 318 | 132 | 761 | 258 | C | 17 | 1.32(1.01;1.73) |  | 1.52(1.11;2.08) | 1.23(0.94;1.61) | 0.03 |
| rs1550109 | 245 | 73 | 834 | 317 | 250 | 101 | 918 | 282 | G | 12 | 1.25(0.92;1.68) |  | 1.39(0.97;1.99) | 1.03(0.76;1.39) | 0.04 |
| rs1562450 | 321 | 95 | 758 | 295 | 322 | 122 | 846 | 261 | G | 47 | 1.35(1.03;1.77) |  | 1.36(0.99;1.88) | 1.07(0.81;1.42) | 0.02 |
| rs1667313 | 268 | 75 | 811 | 315 | 253 | 94 | 916 | 289 | C | 48 | 1.51(1.12;2.03) |  | 1.38(0.96;1.99) | 1.25(0.92;1.68) | 0.04 |
| rs16835263 | 286 | 86 | 883 | 297 | 241 | 113 | 838 | 277 | T | 13 | 1.18(0.89;1.56) |  | 1.6(1.14;2.26) | 1.11(0.84;1.48) | 0.03 |
| rs16852064 | 290 | 86 | 789 | 304 | 283 | 115 | 886 | 268 | T | 14 | 1.35(1.01;1.79) |  | 1.51(1.08;2.11) | 1.05(0.79;1.41) | 0.01 |
| rs17020687 | 1018 | 310 | 151 | 73 | 921 | 339 | 158 | 51 | G | 7 | 1.57(1.14;2.17) |  | 1.18(0.98;1.42) | 1.03(0.73;1.46) | 0.02 |
| rs17190797 | 589 | 184 | 580 | 199 | 489 | 203 | 590 | 187 | T | 28 | 1.19(0.93;1.51) |  | 1.34(1.05;1.71) | 1.04(0.82;1.33) | 0.03 |
| rs17221346 | 702 | 225 | 377 | 165 | 713 | 235 | 456 | 148 | C | 22 | 1.38(1.08;1.77) |  | 1.08(0.86;1.34) | 1.01(0.79;1.3) | 0.04 |
| rs17229285 | 318 | 93 | 851 | 290 | 283 | 125 | 796 | 265 | T | 48 | 1.22(0.93;1.61) |  | 1.52(1.1;2.11) | 1.15(0.87;1.53) | 0.04 |
| rs17405731 | 202 | 49 | 967 | 334 | 144 | 70 | 935 | 320 | G | 8 | 1.45(1.03;2.06) |  | 1.88(1.21;2.92) | 1.42(1;2.01) | 0.04 |
| rs17809667 | 153 | 42 | 924 | 348 | 135 | 60 | 1033 | 322 | C | 7 | 1.42(0.98;2.06) |  | 1.73(1.08;2.78) | 1.19(0.82;1.73) | 0.04 |
| rs1839669 | 239 | 52 | 930 | 331 | 202 | 81 | 877 | 309 | C | 42 | 1.63(1.17;2.29) |  | 1.99(1.32;2.99) | 1.55(1.1;2.18) | 0.02 |
| rs1876860 | 553 | 162 | 616 | 221 | 470 | 186 | 609 | 204 | G | 33 | 1.22(0.96;1.56) |  | 1.35(1.05;1.74) | 1.1(0.86;1.4) | 0.04 |
| rs1922463 | 497 | 142 | 672 | 241 | 402 | 166 | 677 | 224 | G | 36 | 1.29(1.01;1.65) |  | 1.43(1.09;1.88) | 1.16(0.9;1.48) | 0.02 |
| rs2028889 | 720 | 218 | 447 | 164 | 631 | 248 | 448 | 142 | C | 37 | 1.22(0.95;1.55) |  | 1.27(1.02;1.58) | 1.03(0.8;1.33) | 0.04 |
| rs2041837 | 646 | 194 | 523 | 189 | 611 | 235 | 468 | 155 | G | 34 | 1.18(0.93;1.5) |  | 1.28(1.02;1.61) | 1.03(0.8;1.32) | 0.04 |
| rs2042474 | 343 | 107 | 736 | 283 | 372 | 137 | 797 | 246 | C | 18 | 1.32(1.01;1.72) |  | 1.27(0.94;1.72) | 1.06(0.81;1.39) | 0.04 |
| rs2053163 | 135 | 27 | 1034 | 356 | 99 | 49 | 980 | 341 | G | 31 | 1.77(1.14;2.76) |  | 2.47(1.42;4.32) | 1.75(1.12;2.73) | 0.04 |
| rs2063303 | 432 | 122 | 737 | 261 | 361 | 153 | 718 | 237 | G | 41 | 1.22(0.95;1.58) |  | 1.5(1.13;2) | 1.1(0.85;1.42) | 0.02 |
| rs2111470 | 529 | 167 | 550 | 222 | 549 | 186 | 620 | 197 | G | 31 | 1.36(1.07;1.73) |  | 1.16(0.9;1.49) | 1.04(0.81;1.33) | 0.03 |
| rs2113417 | 510 | 150 | 659 | 233 | 398 | 176 | 680 | 214 | G | 23 | 1.19(0.93;1.52) |  | 1.47(1.13;1.92) | 1.04(0.81;1.33) | 0.01 |
| rs2193449 | 752 | 221 | 417 | 162 | 637 | 247 | 442 | 143 | C | 22 | 1.25(0.98;1.6) |  | 1.27(1.02;1.58) | 1.05(0.82;1.34) | 0.03 |
| rs2216329 | 515 | 160 | 564 | 230 | 538 | 180 | 631 | 203 | G | 32 | 1.38(1.08;1.76) |  | 1.16(0.9;1.49) | 1.07(0.83;1.36) | 0.03 |
| rs2222179 | 671 | 195 | 498 | 188 | 574 | 230 | 505 | 160 | C | 26 | 1.27(1;1.61) |  | 1.34(1.06;1.68) | 1.05(0.82;1.35) | 0.01 |
| rs2222180 | 672 | 196 | 497 | 187 | 574 | 230 | 505 | 160 | A | 26 | 1.26(0.99;1.6) |  | 1.33(1.06;1.67) | 1.05(0.82;1.34) | 0.02 |
| rs2222182 | 300 | 89 | 779 | 301 | 295 | 121 | 874 | 262 | T | 14 | 1.4(1.05;1.85) |  | 1.56(1.12;2.17) | 1.07(0.8;1.42) | 0.01 |
| rs2276643 | 445 | 124 | 724 | 259 | 369 | 166 | 710 | 224 | G | 20 | 1.21(0.94;1.55) |  | 1.52(1.15;2.01) | 1.06(0.82;1.37) | 0.01 |
| rs2286321 | 355 | 111 | 724 | 279 | 380 | 142 | 789 | 241 | G | 18 | 1.25(0.96;1.63) |  | 1.25(0.93;1.68) | 1(0.77;1.31) | 0.04 |
| rs2300438 | 917 | 278 | 252 | 105 | 815 | 304 | 264 | 86 | T | 12 | 1.41(1.07;1.85) |  | 1.21(1;1.48) | 1.04(0.78;1.39) | 0.03 |
| rs2365300 | 398 | 108 | 771 | 275 | 333 | 142 | 746 | 248 | G | 18 | 1.29(1;1.68) |  | 1.53(1.13;2.07) | 1.19(0.91;1.55) | 0.02 |
| rs2367797 | 446 | 117 | 723 | 266 | 364 | 149 | 715 | 241 | C | 41 | 1.4(1.08;1.81) |  | 1.56(1.17;2.08) | 1.24(0.95;1.6) | 0.01 |
| rs360259 | 719 | 211 | 449 | 172 | 649 | 255 | 430 | 135 | A | 22 | 1.32(1.04;1.68) |  | 1.28(1.03;1.59) | 1.1(0.85;1.42) | 0.03 |
| rs3845826 | 413 | 123 | 666 | 267 | 426 | 150 | 743 | 233 | T | 40 | 1.36(1.06;1.75) |  | 1.23(0.92;1.63) | 1.08(0.83;1.4) | 0.03 |
| rs4233874 | 375 | 93 | 794 | 290 | 348 | 133 | 730 | 257 | T | 44 | 1.53(1.16;2.01) |  | 1.56(1.14;2.13) | 1.42(1.08;1.88) | 0.03 |
| rs4305317 | 841 | 260 | 328 | 123 | 715 | 278 | 364 | 112 | G | 45 | 1.33(1.02;1.72) |  | 1.26(1.02;1.54) | 1.01(0.78;1.32) | 0.02 |
| rs4461299 | 504 | 135 | 665 | 248 | 428 | 171 | 651 | 219 | C | 36 | 1.32(1.03;1.69) |  | 1.38(1.06;1.81) | 1.21(0.94;1.56) | 0.04 |
| rs4517932 | 249 | 67 | 830 | 323 | 240 | 92 | 929 | 291 | T | 46 | 1.43(1.05;1.94) |  | 1.5(1.04;2.18) | 1.16(0.85;1.58) | 0.02 |
| rs4521036 | 891 | 276 | 278 | 107 | 807 | 303 | 272 | 87 | A | 13 | 1.31(1;1.72) |  | 1.21(0.99;1.47) | 1.02(0.76;1.35) | 0.04 |
| rs4555374 | 489 | 146 | 679 | 236 | 378 | 170 | 701 | 220 | G | 22 | 1.17(0.92;1.5) |  | 1.49(1.14;1.96) | 1.03(0.8;1.32) | 0.01 |
| rs4596023 | 624 | 177 | 544 | 206 | 579 | 221 | 500 | 169 | A | 27 | 1.3(1.02;1.66) |  | 1.31(1.04;1.66) | 1.14(0.89;1.46) | 0.04 |
| rs4629138 | 1014 | 314 | 155 | 69 | 920 | 338 | 159 | 52 | C | 8 | 1.47(1.07;2.02) |  | 1.17(0.97;1.41) | 1.02(0.72;1.44) | 0.04 |
| rs4637147 | 1007 | 305 | 161 | 78 | 935 | 345 | 143 | 45 | C | 7 | 1.62(1.19;2.22) |  | 1.2(0.99;1.44) | 1.01(0.7;1.46) | 0.01 |
| rs4666343 | 681 | 196 | 488 | 187 | 620 | 235 | 459 | 155 | T | 24 | 1.39(1.1;1.77) |  | 1.34(1.06;1.68) | 1.15(0.89;1.47) | 0.01 |
| rs4668076 | 956 | 283 | 213 | 100 | 873 | 316 | 206 | 74 | A | 10 | 1.7(1.28;2.26) |  | 1.23(1.01;1.49) | 1.14(0.84;1.56) | 0.01 |
| rs4669413 | 409 | 106 | 759 | 277 | 369 | 151 | 710 | 239 | T | 42 | 1.39(1.07;1.81) |  | 1.56(1.16;2.09) | 1.24(0.95;1.63) | 0.01 |
| rs4854249 | 508 | 144 | 660 | 239 | 390 | 177 | 689 | 213 | A | 23 | 1.27(1;1.63) |  | 1.58(1.21;2.07) | 1.06(0.83;1.36) | 0.00 |
| rs4854550 | 420 | 127 | 659 | 263 | 423 | 150 | 746 | 233 | T | 39 | 1.37(1.06;1.76) |  | 1.24(0.93;1.65) | 1.07(0.83;1.39) | 0.03 |
| rs4893907 | 465 | 134 | 612 | 251 | 523 | 181 | 641 | 201 | C | 33 | 1.4(1.09;1.8) |  | 1.22(0.93;1.59) | 1.1(0.85;1.42) | 0.03 |
| rs4973417 | 186 | 44 | 893 | 346 | 174 | 70 | 995 | 313 | T | 41 | 1.79(1.25;2.57) |  | 1.85(1.18;2.88) | 1.47(1.02;2.11) | 0.02 |
| rs6430676 | 983 | 303 | 186 | 80 | 856 | 317 | 223 | 73 | C | 10 | 1.46(1.08;1.98) |  | 1.2(0.99;1.45) | 1.01(0.74;1.36) | 0.02 |
| rs6431210 | 936 | 285 | 225 | 97 | 837 | 311 | 236 | 77 | A | 11 | 1.51(1.14;2.01) |  | 1.21(1;1.47) | 1.06(0.78;1.43) | 0.02 |
| rs6546637 | 298 | 91 | 780 | 299 | 331 | 124 | 838 | 259 | C | 48 | 1.29(0.97;1.7) |  | 1.3(0.94;1.81) | 1.05(0.79;1.39) | 0.04 |
| rs6717854 | 216 | 49 | 953 | 334 | 184 | 75 | 895 | 315 | A | 41 | 1.58(1.12;2.23) |  | 1.82(1.19;2.79) | 1.54(1.09;2.18) | 0.04 |
| rs6722368 | 294 | 84 | 785 | 305 | 273 | 110 | 894 | 273 | T | 14 | 1.33(1;1.76) |  | 1.49(1.06;2.1) | 1.05(0.79;1.4) | 0.01 |
| rs6723757 | 1013 | 307 | 153 | 76 | 940 | 346 | 139 | 44 | A | 7 | 1.68(1.22;2.31) |  | 1.2(0.99;1.44) | 1.02(0.7;1.47) | 0.01 |
| rs6725139 | 769 | 229 | 400 | 154 | 673 | 259 | 406 | 131 | C | 40 | 1.29(1.01;1.65) |  | 1.26(1.01;1.56) | 1.07(0.83;1.39) | 0.03 |
| rs6731818 | 779 | 242 | 385 | 141 | 711 | 268 | 367 | 122 | G | 43 | 1.32(1.03;1.7) |  | 1.24(1;1.53) | 1.08(0.84;1.41) | 0.04 |
| rs6741066 | 652 | 191 | 516 | 192 | 551 | 218 | 528 | 172 | C | 27 | 1.23(0.97;1.57) |  | 1.3(1.03;1.64) | 1.08(0.85;1.38) | 0.04 |
| rs6745182 | 919 | 278 | 250 | 105 | 839 | 310 | 240 | 80 | C | 12 | 1.39(1.05;1.82) |  | 1.2(0.99;1.46) | 1.07(0.79;1.44) | 0.04 |
| rs6748302 | 995 | 306 | 174 | 77 | 886 | 325 | 193 | 65 | T | 9 | 1.48(1.09;2.01) |  | 1.18(0.98;1.42) | 1.04(0.76;1.44) | 0.04 |
| rs7425848 | 294 | 87 | 784 | 303 | 286 | 118 | 883 | 265 | T | 14 | 1.37(1.03;1.81) |  | 1.54(1.11;2.15) | 1.05(0.79;1.4) | 0.01 |
| rs7425888 | 890 | 276 | 277 | 106 | 806 | 303 | 272 | 86 | A | 13 | 1.3(0.99;1.7) |  | 1.21(0.99;1.47) | 1(0.75;1.33) | 0.04 |
| rs753439 | 460 | 139 | 709 | 244 | 422 | 177 | 657 | 213 | C | 22 | 1.1(0.86;1.41) |  | 1.38(1.05;1.8) | 1(0.78;1.29) | 0.04 |
| rs7563350 | 779 | 226 | 390 | 157 | 695 | 255 | 384 | 135 | T | 20 | 1.34(1.05;1.72) |  | 1.25(1.01;1.55) | 1.12(0.87;1.45) | 0.04 |
| rs7568847 | 751 | 220 | 418 | 163 | 634 | 243 | 445 | 147 | A | 22 | 1.27(0.99;1.62) |  | 1.26(1.02;1.57) | 1.07(0.84;1.37) | 0.04 |
| rs7569110 | 291 | 86 | 788 | 304 | 285 | 116 | 884 | 267 | C | 14 | 1.37(1.03;1.82) |  | 1.52(1.09;2.13) | 1.06(0.8;1.42) | 0.01 |
| rs7582092 | 509 | 142 | 660 | 241 | 423 | 170 | 656 | 220 | A | 36 | 1.32(1.03;1.69) |  | 1.4(1.07;1.84) | 1.19(0.93;1.53) | 0.03 |
| rs7585067 | 188 | 42 | 981 | 341 | 146 | 59 | 933 | 331 | C | 38 | 1.6(1.1;2.32) |  | 1.97(1.23;3.15) | 1.57(1.08;2.28) | 0.04 |
| rs7594526 | 388 | 104 | 781 | 279 | 353 | 146 | 726 | 244 | G | 43 | 1.32(1.01;1.72) |  | 1.5(1.11;2.03) | 1.22(0.93;1.6) | 0.03 |
| rs7599598 | 353 | 93 | 816 | 290 | 300 | 129 | 779 | 261 | G | 47 | 1.3(0.99;1.71) |  | 1.64(1.19;2.25) | 1.18(0.9;1.57) | 0.01 |
| rs7600163 | 221 | 61 | 858 | 329 | 210 | 82 | 959 | 301 | G | 43 | 1.37(0.99;1.89) |  | 1.48(1;2.21) | 1.14(0.83;1.58) | 0.04 |
| rs869186 | 468 | 134 | 611 | 256 | 421 | 157 | 748 | 226 | A | 38 | 1.5(1.17;1.93) |  | 1.34(1.02;1.77) | 1.1(0.86;1.42) | 0.00 |
| rs891500 | 305 | 94 | 774 | 296 | 323 | 124 | 846 | 259 | C | 16 | 1.35(1.02;1.78) |  | 1.33(0.97;1.84) | 1.08(0.82;1.43) | 0.03 |
| rs9309111 | 383 | 106 | 786 | 276 | 346 | 148 | 732 | 242 | G | 43 | 1.25(0.96;1.63) |  | 1.5(1.11;2.02) | 1.16(0.89;1.52) | 0.03 |
| rs997400 | 297 | 82 | 782 | 308 | 279 | 116 | 890 | 267 | T | 49 | 1.56(1.17;2.08) |  | 1.58(1.12;2.22) | 1.21(0.9;1.61) | 0.01 |
| chr 3 |  |  |  |  |  |  |  |  |  |  |  |  |  |  |  |
| rs10513452 | 875 | 258 | 294 | 125 | 749 | 280 | 330 | 110 | C | 16 | 1.44(1.11;1.87) |  | 1.22(1;1.5) | 1.15(0.88;1.5) | 0.04 |
| rs10935002 | 508 | 140 | 661 | 243 | 420 | 169 | 659 | 221 | G | 36 | 1.31(1.02;1.67) |  | 1.41(1.08;1.85) | 1.17(0.91;1.51) | 0.03 |
| rs11705826 | 606 | 185 | 563 | 198 | 547 | 220 | 532 | 170 | T | 31 | 1.19(0.94;1.52) |  | 1.31(1.03;1.66) | 1.05(0.82;1.34) | 0.04 |
| rs11706137 | 451 | 132 | 718 | 251 | 398 | 167 | 681 | 223 | C | 21 | 1.16(0.9;1.49) |  | 1.4(1.06;1.84) | 1.06(0.82;1.37) | 0.04 |
| rs11707159 | 840 | 244 | 327 | 138 | 745 | 285 | 334 | 104 | T | 16 | 1.51(1.17;1.94) |  | 1.32(1.07;1.62) | 1.05(0.8;1.37) | 0.00 |
| rs11715796 | 127 | 26 | 1042 | 357 | 104 | 57 | 975 | 333 | C | 5 | 1.74(1.1;2.76) |  | 2.51(1.44;4.38) | 1.71(1.08;2.72) | 0.04 |
| rs12485391 | 250 | 76 | 919 | 307 | 190 | 99 | 889 | 291 | A | 11 | 1.07(0.79;1.43) |  | 1.71(1.18;2.46) | 1.02(0.75;1.37) | 0.03 |
| rs12490823 | 533 | 149 | 636 | 234 | 444 | 176 | 634 | 214 | T | 34 | 1.28(1.01;1.64) |  | 1.38(1.06;1.79) | 1.15(0.9;1.48) | 0.03 |
| rs12490860 | 469 | 140 | 700 | 243 | 391 | 163 | 688 | 227 | G | 38 | 1.14(0.89;1.46) |  | 1.41(1.07;1.85) | 1.04(0.81;1.33) | 0.03 |
| rs12493885 | 883 | 258 | 286 | 125 | 764 | 289 | 315 | 101 | G | 15 | 1.48(1.14;1.92) |  | 1.26(1.03;1.54) | 1.09(0.83;1.43) | 0.01 |
| rs12496701 | 726 | 234 | 353 | 156 | 748 | 250 | 421 | 133 | T | 19 | 1.43(1.12;1.83) |  | 1.08(0.87;1.33) | 1.02(0.79;1.31) | 0.03 |
| rs13085173 | 997 | 284 | 171 | 99 | 870 | 323 | 207 | 67 | G | 10 | 1.91(1.43;2.56) |  | 1.27(1.05;1.53) | 1.1(0.8;1.51) | 0.00 |
| rs13086896 | 629 | 172 | 540 | 210 | 563 | 212 | 516 | 178 | G | 28 | 1.47(1.15;1.87) |  | 1.34(1.06;1.71) | 1.28(1;1.64) | 0.03 |
| rs1345154 | 197 | 57 | 864 | 323 | 203 | 86 | 938 | 290 | G | 10 | 1.32(0.95;1.84) |  | 1.53(1.02;2.28) | 1.11(0.8;1.55) | 0.04 |
| rs1351235 | 306 | 91 | 862 | 292 | 232 | 115 | 845 | 275 | C | 13 | 1.12(0.85;1.48) |  | 1.65(1.18;2.3) | 1.05(0.79;1.39) | 0.02 |
| rs1379195 | 429 | 122 | 650 | 268 | 442 | 159 | 727 | 224 | T | 38 | 1.37(1.07;1.77) |  | 1.26(0.95;1.66) | 1.07(0.82;1.38) | 0.02 |
| rs1436634 | 189 | 52 | 980 | 331 | 147 | 73 | 932 | 317 | A | 8 | 1.31(0.92;1.85) |  | 1.83(1.19;2.84) | 1.28(0.91;1.82) | 0.04 |
| rs1447595 | 246 | 73 | 833 | 317 | 253 | 104 | 916 | 279 | G | 12 | 1.32(0.98;1.79) |  | 1.43(1;2.05) | 1.08(0.8;1.47) | 0.03 |
| rs1452201 | 219 | 54 | 950 | 329 | 198 | 88 | 881 | 302 | T | 42 | 1.39(0.99;1.94) |  | 1.9(1.26;2.85) | 1.32(0.94;1.86) | 0.02 |
| rs1453744 | 355 | 106 | 804 | 275 | 292 | 132 | 784 | 258 | T | 16 | 1.14(0.87;1.49) |  | 1.48(1.09;2.02) | 1.07(0.82;1.4) | 0.04 |
| rs1472597 | 693 | 210 | 386 | 180 | 755 | 249 | 414 | 134 | G | 21 | 1.48(1.16;1.89) |  | 1.08(0.87;1.35) | 1.1(0.85;1.42) | 0.04 |
| rs1479176 | 316 | 95 | 853 | 288 | 242 | 119 | 837 | 271 | C | 14 | 1.1(0.83;1.44) |  | 1.58(1.14;2.2) | 1.03(0.78;1.36) | 0.03 |
| rs1482272 | 533 | 156 | 636 | 227 | 459 | 186 | 619 | 204 | T | 26 | 1.23(0.97;1.57) |  | 1.37(1.06;1.77) | 1.11(0.86;1.42) | 0.03 |
| rs1490543 | 473 | 136 | 606 | 254 | 471 | 163 | 698 | 220 | G | 36 | 1.48(1.16;1.9) |  | 1.19(0.91;1.57) | 1.16(0.9;1.49) | 0.03 |
| rs1515653 | 880 | 252 | 289 | 131 | 756 | 290 | 323 | 100 | A | 15 | 1.58(1.22;2.04) |  | 1.29(1.06;1.59) | 1.1(0.83;1.44) | 0.00 |
| rs1530735 | 458 | 117 | 710 | 266 | 348 | 139 | 730 | 251 | C | 41 | 1.57(1.21;2.03) |  | 1.63(1.21;2.18) | 1.36(1.05;1.77) | 0.01 |
| rs1532719 | 763 | 224 | 406 | 159 | 671 | 250 | 408 | 140 | T | 21 | 1.34(1.05;1.71) |  | 1.29(1.04;1.6) | 1.08(0.84;1.39) | 0.02 |
| rs16825520 | 931 | 280 | 238 | 103 | 845 | 318 | 234 | 72 | T | 11 | 1.37(1.04;1.81) |  | 1.21(0.99;1.46) | 1(0.74;1.36) | 0.03 |
| rs17068844 | 722 | 212 | 447 | 171 | 627 | 252 | 452 | 138 | C | 23 | 1.35(1.06;1.72) |  | 1.35(1.08;1.69) | 1.04(0.81;1.34) | 0.01 |
| rs1711005 | 935 | 281 | 234 | 102 | 817 | 312 | 262 | 78 | A | 12 | 1.45(1.1;1.92) |  | 1.22(1.01;1.49) | 1.02(0.76;1.37) | 0.01 |
| rs17214746 | 339 | 99 | 740 | 291 | 319 | 120 | 850 | 263 | G | 16 | 1.32(1.01;1.72) |  | 1.3(0.95;1.79) | 1.06(0.81;1.39) | 0.03 |
| rs2044569 | 243 | 71 | 836 | 319 | 249 | 104 | 920 | 279 | G | 12 | 1.35(0.99;1.83) |  | 1.48(1.03;2.13) | 1.09(0.8;1.49) | 0.02 |
| rs2202157 | 861 | 266 | 308 | 117 | 749 | 281 | 330 | 109 | T | 16 | 1.27(0.97;1.65) |  | 1.22(1;1.49) | 1.01(0.77;1.32) | 0.04 |
| rs2727926 | 335 | 86 | 834 | 297 | 272 | 110 | 807 | 280 | T | 49 | 1.45(1.09;1.92) |  | 1.56(1.11;2.19) | 1.38(1.03;1.83) | 0.04 |
| rs2929358 | 901 | 267 | 267 | 116 | 800 | 296 | 279 | 94 | C | 13 | 1.44(1.1;1.88) |  | 1.22(1;1.49) | 1.09(0.82;1.44) | 0.03 |
| rs355752 | 879 | 251 | 290 | 132 | 759 | 290 | 320 | 100 | A | 15 | 1.59(1.23;2.06) |  | 1.3(1.06;1.59) | 1.1(0.84;1.45) | 0.00 |
| rs355768 | 882 | 251 | 287 | 132 | 761 | 290 | 318 | 100 | A | 15 | 1.62(1.25;2.09) |  | 1.3(1.06;1.59) | 1.11(0.85;1.46) | 0.00 |
| rs357502 | 676 | 191 | 493 | 192 | 608 | 225 | 470 | 165 | C | 25 | 1.45(1.14;1.85) |  | 1.3(1.03;1.64) | 1.26(0.98;1.62) | 0.04 |
| rs358007 | 160 | 35 | 1009 | 348 | 122 | 59 | 957 | 331 | T | 35 | 1.5(1.01;2.22) |  | 2.18(1.33;3.57) | 1.46(0.99;2.18) | 0.03 |
| rs3772339 | 468 | 125 | 700 | 258 | 397 | 151 | 682 | 239 | A | 38 | 1.38(1.07;1.78) |  | 1.44(1.09;1.91) | 1.26(0.98;1.63) | 0.03 |
| rs3846075 | 477 | 136 | 602 | 254 | 474 | 163 | 695 | 220 | T | 36 | 1.51(1.17;1.93) |  | 1.2(0.91;1.57) | 1.17(0.91;1.51) | 0.03 |
| rs3856827 | 248 | 79 | 813 | 307 | 255 | 110 | 890 | 270 | G | 13 | 1.25(0.94;1.68) |  | 1.4(0.99;1.99) | 1.02(0.76;1.38) | 0.03 |
| rs3915052 | 353 | 103 | 816 | 280 | 327 | 134 | 752 | 256 | A | 16 | 1.14(0.88;1.49) |  | 1.46(1.08;1.99) | 1.07(0.82;1.41) | 0.04 |
| rs403286 | 230 | 58 | 849 | 332 | 252 | 96 | 916 | 287 | A | 46 | 1.53(1.11;2.12) |  | 1.5(1.02;2.2) | 1.27(0.91;1.76) | 0.03 |
| rs4399912 | 515 | 160 | 564 | 230 | 523 | 188 | 646 | 195 | T | 33 | 1.35(1.06;1.72) |  | 1.18(0.92;1.52) | 1.02(0.8;1.31) | 0.02 |
| rs4482697 | 503 | 140 | 666 | 243 | 447 | 178 | 632 | 212 | T | 35 | 1.4(1.1;1.8) |  | 1.44(1.11;1.89) | 1.24(0.96;1.6) | 0.02 |
| rs4603925 | 324 | 80 | 845 | 303 | 254 | 105 | 825 | 285 | A | 50 | 1.47(1.1;1.96) |  | 1.73(1.23;2.45) | 1.36(1.02;1.82) | 0.01 |
| rs4642101 | 470 | 129 | 675 | 247 | 415 | 167 | 657 | 215 | T | 38 | 1.31(1.01;1.68) |  | 1.4(1.06;1.84) | 1.16(0.9;1.51) | 0.03 |
| rs4679323 | 588 | 169 | 577 | 213 | 563 | 212 | 514 | 177 | C | 29 | 1.35(1.06;1.72) |  | 1.32(1.03;1.68) | 1.2(0.93;1.54) | 0.04 |
| rs4685279 | 119 | 31 | 960 | 359 | 79 | 41 | ### | 342 | T | 29 | 1.51(0.99;2.3) |  | 2.12(1.21;3.72) | 1.29(0.84;1.97) | 0.04 |
| rs4854914 | 315 | 97 | 854 | 286 | 243 | 119 | 836 | 271 | A | 14 | 1.07(0.82;1.41) |  | 1.56(1.13;2.17) | 1.01(0.77;1.33) | 0.03 |
| rs4854992 | 365 | 104 | 804 | 279 | 290 | 128 | 789 | 262 | C | 16 | 1.22(0.94;1.59) |  | 1.58(1.16;2.16) | 1.12(0.86;1.47) | 0.02 |
| rs4855026 | 294 | 85 | 875 | 298 | 227 | 114 | 852 | 276 | A | 13 | 1.16(0.87;1.54) |  | 1.72(1.23;2.42) | 1.07(0.8;1.43) | 0.01 |
| rs4855028 | 306 | 91 | 863 | 292 | 232 | 115 | 847 | 275 | A | 13 | 1.12(0.85;1.48) |  | 1.65(1.18;2.3) | 1.05(0.79;1.39) | 0.02 |
| rs491976 | 955 | 291 | 214 | 92 | 849 | 318 | 230 | 72 | C | 45 | 1.44(1.08;1.91) |  | 1.2(0.99;1.46) | 1.03(0.76;1.39) | 0.02 |
| rs4974445 | 567 | 170 | 599 | 213 | 497 | 205 | 580 | 185 | C | 28 | 1.17(0.92;1.49) |  | 1.33(1.04;1.7) | 1.04(0.81;1.33) | 0.03 |
| rs6445062 | 265 | 79 | 814 | 311 | 260 | 107 | 909 | 276 | T | 48 | 1.29(0.96;1.72) |  | 1.47(1.04;2.08) | 1.03(0.77;1.38) | 0.02 |
| rs670752 | 556 | 166 | 522 | 224 | 544 | 187 | 625 | 196 | A | 31 | 1.37(1.08;1.74) |  | 1.18(0.92;1.52) | 1.02(0.8;1.3) | 0.02 |
| rs6764257 | 309 | 89 | 770 | 301 | 312 | 125 | 857 | 258 | A | 47 | 1.36(1.03;1.8) |  | 1.42(1.02;1.96) | 1.07(0.81;1.42) | 0.02 |
| rs6782970 | 318 | 97 | 851 | 286 | 245 | 120 | 834 | 270 | C | 14 | 1.08(0.82;1.42) |  | 1.57(1.13;2.18) | 1.01(0.77;1.34) | 0.03 |
| rs6796462 | 764 | 225 | 404 | 158 | 659 | 261 | 420 | 129 | A | 21 | 1.36(1.06;1.74) |  | 1.32(1.06;1.63) | 1.04(0.8;1.34) | 0.01 |
| rs6803590 | 357 | 107 | 812 | 276 | 325 | 144 | 754 | 246 | G | 17 | 1.09(0.84;1.42) |  | 1.44(1.07;1.95) | 1.02(0.78;1.34) | 0.04 |
| rs701116 | 1003 | 301 | 166 | 82 | 896 | 328 | 183 | 62 | T | 9 | 1.67(1.23;2.27) |  | 1.19(0.99;1.44) | 1.13(0.81;1.56) | 0.02 |
| rs701147 | 880 | 251 | 289 | 132 | 760 | 289 | 319 | 101 | C | 15 | 1.6(1.23;2.07) |  | 1.29(1.05;1.58) | 1.12(0.85;1.46) | 0.00 |
| rs7372712 | 808 | 246 | 361 | 137 | 698 | 269 | 381 | 121 | T | 18 | 1.29(1.01;1.66) |  | 1.25(1.01;1.54) | 1.05(0.81;1.36) | 0.03 |
| rs7373337 | 308 | 91 | 861 | 292 | 233 | 115 | 846 | 275 | C | 13 | 1.13(0.86;1.49) |  | 1.65(1.18;2.3) | 1.06(0.8;1.4) | 0.02 |
| rs7374605 | 248 | 68 | 828 | 321 | 234 | 92 | 926 | 289 | C | 46 | 1.41(1.04;1.91) |  | 1.49(1.02;2.16) | 1.16(0.85;1.58) | 0.03 |
| rs7430029 | 346 | 101 | 822 | 280 | 281 | 124 | 797 | 266 | C | 15 | 1.24(0.95;1.63) |  | 1.5(1.09;2.07) | 1.18(0.9;1.56) | 0.04 |
| rs749948 | 372 | 96 | 796 | 287 | 326 | 130 | 751 | 259 | T | 45 | 1.34(1.03;1.76) |  | 1.48(1.08;2.03) | 1.26(0.96;1.66) | 0.04 |
| rs7611361 | 316 | 95 | 853 | 288 | 242 | 118 | 837 | 272 | A | 14 | 1.1(0.83;1.44) |  | 1.57(1.13;2.18) | 1.04(0.79;1.37) | 0.03 |
| rs7615255 | 649 | 190 | 520 | 193 | 551 | 220 | 528 | 170 | C | 27 | 1.25(0.98;1.59) |  | 1.33(1.05;1.68) | 1.07(0.83;1.36) | 0.02 |
| rs7630621 | 597 | 172 | 542 | 198 | 498 | 204 | 543 | 170 | G | 30 | 1.29(1.01;1.65) |  | 1.39(1.09;1.78) | 1.07(0.84;1.38) | 0.01 |
| rs7633040 | 282 | 79 | 797 | 311 | 279 | 111 | 890 | 272 | G | 13 | 1.47(1.1;1.97) |  | 1.52(1.08;2.15) | 1.16(0.86;1.56) | 0.01 |
| rs7637842 | 664 | 201 | 505 | 182 | 574 | 221 | 505 | 169 | C | 26 | 1.25(0.98;1.58) |  | 1.31(1.04;1.65) | 1.06(0.83;1.35) | 0.03 |
| rs7646152 | 567 | 166 | 602 | 217 | 498 | 200 | 581 | 190 | G | 27 | 1.29(1.01;1.64) |  | 1.37(1.07;1.76) | 1.12(0.88;1.44) | 0.02 |
| rs789296 | 404 | 107 | 763 | 276 | 335 | 149 | 744 | 241 | A | 42 | 1.38(1.06;1.8) |  | 1.58(1.17;2.13) | 1.24(0.95;1.62) | 0.01 |
| rs820336 | 610 | 180 | 469 | 210 | 642 | 205 | 527 | 178 | C | 27 | 1.48(1.16;1.88) |  | 1.14(0.9;1.44) | 1.1(0.86;1.41) | 0.02 |
| rs9815301 | 229 | 56 | 940 | 327 | 174 | 78 | 905 | 312 | C | 41 | 1.47(1.05;2.05) |  | 1.89(1.25;2.86) | 1.41(1.01;1.98) | 0.03 |
| rs9816337 | 852 | 257 | 317 | 126 | 777 | 296 | 302 | 94 | T | 48 | 1.29(1;1.67) |  | 1.23(1;1.5) | 1.01(0.76;1.34) | 0.03 |
| rs9832986 | 227 | 58 | 852 | 332 | 183 | 71 | 986 | 312 | T | 42 | 1.55(1.12;2.15) |  | 1.65(1.1;2.49) | 1.27(0.92;1.76) | 0.02 |
| rs9851100 | 539 | 151 | 630 | 232 | 451 | 180 | 628 | 210 | A | 34 | 1.28(1;1.64) |  | 1.4(1.08;1.81) | 1.14(0.89;1.46) | 0.02 |
| rs9851119 | 386 | 99 | 782 | 284 | 310 | 129 | 769 | 261 | C | 46 | 1.35(1.03;1.76) |  | 1.59(1.17;2.18) | 1.23(0.94;1.61) | 0.02 |
| rs9853486 | 118 | 28 | 961 | 361 | 136 | 62 | 1033 | 321 | C | 6 | 1.58(1.02;2.46) |  | 2.06(1.22;3.48) | 1.33(0.86;2.07) | 0.03 |
| rs9869163 | 760 | 225 | 409 | 158 | 654 | 249 | 425 | 141 | A | 21 | 1.32(1.04;1.69) |  | 1.31(1.05;1.62) | 1.04(0.81;1.34) | 0.01 |
| rs9870651 | 434 | 123 | 694 | 250 | 376 | 152 | 674 | 224 | T | 40 | 1.22(0.94;1.58) |  | 1.39(1.05;1.85) | 1.11(0.86;1.45) | 0.04 |
| rs9873218 | 355 | 103 | 813 | 280 | 321 | 135 | 758 | 255 | A | 16 | 1.16(0.89;1.52) |  | 1.52(1.12;2.07) | 1.07(0.82;1.4) | 0.02 |
| rs9877018 | 138 | 41 | 941 | 349 | 107 | 60 | ### | 323 | A | 6 | 1.29(0.87;1.9) |  | 1.86(1.14;3.05) | 1.09(0.74;1.6) | 0.04 |
| rs9878325 | 706 | 211 | 463 | 172 | 622 | 239 | 457 | 151 | A | 36 | 1.28(1.01;1.63) |  | 1.27(1.02;1.59) | 1.1(0.86;1.42) | 0.04 |
| chr 4 |  |  |  |  |  |  |  |  |  |  |  |  |  |  |  |
| rs10002944 | 659 | 199 | 510 | 184 | 553 | 223 | 526 | 167 | A | 32 | 1.17(0.92;1.49) |  | 1.3(1.03;1.63) | 1.02(0.8;1.3) | 0.03 |
| rs10019779 | 255 | 77 | 824 | 313 | 241 | 113 | 928 | 270 | C | 12 | 1.38(1.03;1.86) |  | 1.62(1.14;2.31) | 1.08(0.8;1.45) | 0.01 |
| rs10021978 | 132 | 22 | 1037 | 361 | 98 | 43 | 981 | 347 | G | 31 | 2.05(1.26;3.34) |  | 2.75(1.51;4.99) | 2.03(1.25;3.3) | 0.04 |
| rs10033555 | 581 | 168 | 588 | 215 | 520 | 208 | 559 | 182 | T | 31 | 1.26(0.99;1.6) |  | 1.35(1.06;1.73) | 1.1(0.86;1.41) | 0.02 |
| rs1010816 | 106 | 15 | 1063 | 368 | 77 | 38 | ### | 352 | T | 28 | 2.54(1.45;4.46) |  | 3.43(1.74;6.78) | 2.52(1.43;4.42) | 0.04 |
| rs1013411 | 690 | 214 | 479 | 169 | 603 | 237 | 476 | 153 | C | 35 | 1.2(0.94;1.53) |  | 1.27(1.02;1.59) | 1.04(0.81;1.33) | 0.04 |
| rs10213453 | 302 | 85 | 867 | 298 | 250 | 110 | 829 | 280 | A | 13 | 1.2(0.9;1.6) |  | 1.55(1.1;2.18) | 1.15(0.86;1.53) | 0.04 |
| rs10517692 | 383 | 119 | 785 | 264 | 312 | 140 | 766 | 250 | A | 17 | 1.09(0.84;1.41) |  | 1.48(1.1;2) | 1.01(0.78;1.31) | 0.03 |
| rs10805344 | 866 | 263 | 303 | 120 | 786 | 294 | 293 | 96 | C | 15 | 1.34(1.03;1.75) |  | 1.22(1;1.49) | 1.05(0.8;1.39) | 0.04 |
| rs11735290 | 933 | 275 | 232 | 103 | 864 | 319 | 210 | 70 | G | 11 | 1.55(1.17;2.05) |  | 1.25(1.03;1.51) | 1.09(0.8;1.49) | 0.01 |
| rs11933202 | 460 | 137 | 709 | 246 | 427 | 178 | 652 | 212 | A | 23 | 1.16(0.9;1.48) |  | 1.39(1.06;1.81) | 1.05(0.81;1.35) | 0.03 |
| rs11934855 | 476 | 143 | 693 | 240 | 409 | 166 | 670 | 224 | C | 37 | 1.25(0.98;1.6) |  | 1.38(1.05;1.8) | 1.15(0.9;1.48) | 0.04 |
| rs11935111 | 630 | 187 | 538 | 196 | 533 | 217 | 546 | 173 | C | 31 | 1.2(0.95;1.53) |  | 1.33(1.05;1.69) | 1.03(0.81;1.32) | 0.02 |
| rs11943753 | 1019 | 314 | 150 | 69 | 952 | 347 | 127 | 43 | A | 7 | 1.52(1.1;2.1) |  | 1.17(0.97;1.41) | 1.01(0.69;1.47) | 0.03 |
| rs12499097 | 574 | 168 | 595 | 215 | 497 | 194 | 582 | 196 | A | 31 | 1.26(0.99;1.6) |  | 1.34(1.04;1.71) | 1.12(0.88;1.43) | 0.04 |
| rs12503242 | 376 | 91 | 703 | 299 | 345 | 112 | 824 | 271 | G | 45 | 1.73(1.32;2.28) |  | 1.31(0.94;1.81) | 1.4(1.06;1.85) | 0.03 |
| rs12511322 | 777 | 228 | 392 | 155 | 672 | 256 | 407 | 134 | A | 20 | 1.39(1.08;1.77) |  | 1.32(1.06;1.63) | 1.06(0.82;1.37) | 0.01 |
| rs12640416 | 461 | 131 | 708 | 252 | 405 | 171 | 674 | 219 | C | 38 | 1.25(0.97;1.6) |  | 1.5(1.14;1.97) | 1.09(0.85;1.42) | 0.01 |
| rs13109149 | 388 | 111 | 781 | 272 | 329 | 144 | 750 | 246 | T | 18 | 1.23(0.94;1.59) |  | 1.52(1.13;2.04) | 1.12(0.86;1.46) | 0.02 |
| rs13118643 | 509 | 147 | 660 | 236 | 443 | 181 | 636 | 209 | C | 35 | 1.26(0.99;1.61) |  | 1.4(1.08;1.82) | 1.12(0.87;1.44) | 0.02 |
| rs13123581 | 386 | 105 | 783 | 278 | 311 | 139 | 768 | 251 | A | 44 | 1.44(1.1;1.87) |  | 1.72(1.27;2.34) | 1.25(0.96;1.64) | 0.00 |
| rs13123614 | 479 | 132 | 690 | 251 | 414 | 172 | 665 | 218 | G | 37 | 1.39(1.08;1.79) |  | 1.55(1.18;2.04) | 1.19(0.92;1.54) | 0.01 |
| rs13138121 | 375 | 107 | 704 | 283 | 362 | 132 | 807 | 251 | A | 43 | 1.42(1.09;1.85) |  | 1.29(0.95;1.75) | 1.14(0.87;1.49) | 0.03 |
| rs13139892 | 358 | 105 | 811 | 278 | 295 | 134 | 784 | 256 | C | 16 | 1.19(0.91;1.56) |  | 1.62(1.18;2.2) | 1.08(0.83;1.42) | 0.01 |
| rs13147507 | 369 | 101 | 710 | 289 | 347 | 131 | 822 | 252 | G | 44 | 1.51(1.16;1.98) |  | 1.39(1.02;1.9) | 1.18(0.9;1.54) | 0.01 |
| rs13148020 | 301 | 91 | 778 | 299 | 315 | 118 | 854 | 265 | C | 49 | 1.31(0.99;1.73) |  | 1.33(0.96;1.84) | 1.06(0.8;1.41) | 0.04 |
| rs1350180 | 291 | 83 | 787 | 307 | 277 | 100 | 892 | 283 | G | 50 | 1.45(1.09;1.93) |  | 1.33(0.94;1.88) | 1.19(0.89;1.59) | 0.04 |
| rs13634 | 714 | 204 | 454 | 179 | 629 | 248 | 450 | 142 | T | 23 | 1.37(1.07;1.74) |  | 1.37(1.1;1.72) | 1.05(0.81;1.35) | 0.00 |
| rs1380000 | 460 | 139 | 619 | 251 | 444 | 166 | 719 | 217 | T | 37 | 1.35(1.06;1.73) |  | 1.19(0.91;1.56) | 1.07(0.83;1.37) | 0.04 |
| rs1385761 | 129 | 22 | 1040 | 361 | 101 | 44 | 978 | 346 | C | 31 | 2.01(1.23;3.26) |  | 2.64(1.46;4.79) | 1.99(1.22;3.24) | 0.04 |
| rs1445428 | 489 | 140 | 680 | 243 | 417 | 176 | 662 | 214 | G | 37 | 1.21(0.94;1.55) |  | 1.47(1.13;1.92) | 1.05(0.82;1.36) | 0.01 |
| rs1450932 | 339 | 92 | 740 | 297 | 335 | 117 | 833 | 266 | A | 46 | 1.5(1.14;1.97) |  | 1.38(1;1.9) | 1.2(0.91;1.58) | 0.02 |
| rs1588411 | 483 | 148 | 596 | 242 | 479 | 164 | 690 | 219 | T | 35 | 1.35(1.05;1.72) |  | 1.17(0.9;1.53) | 1.06(0.82;1.36) | 0.04 |
| rs16868227 | 937 | 285 | 232 | 98 | 862 | 322 | 217 | 68 | G | 11 | 1.44(1.09;1.91) |  | 1.21(1;1.46) | 1.03(0.75;1.4) | 0.02 |
| rs17026427 | 284 | 87 | 794 | 303 | 254 | 104 | 913 | 279 | C | 13 | 1.33(1;1.76) |  | 1.38(0.98;1.95) | 1.08(0.81;1.44) | 0.03 |
| rs17028431 | 195 | 59 | 884 | 331 | 179 | 78 | 990 | 305 | A | 9 | 1.32(0.95;1.83) |  | 1.54(1.02;2.33) | 1.1(0.79;1.53) | 0.04 |
| rs17064673 | 211 | 60 | 956 | 323 | 159 | 78 | 920 | 312 | C | 9 | 1.21(0.87;1.67) |  | 1.78(1.18;2.68) | 1.17(0.85;1.63) | 0.04 |
| rs1712492 | 715 | 205 | 454 | 178 | 637 | 248 | 442 | 142 | T | 23 | 1.36(1.07;1.73) |  | 1.36(1.08;1.69) | 1.06(0.83;1.37) | 0.01 |
| rs17219243 | 359 | 105 | 810 | 278 | 295 | 133 | 784 | 257 | A | 16 | 1.2(0.92;1.56) |  | 1.61(1.18;2.19) | 1.09(0.83;1.43) | 0.01 |
| rs17323516 | 879 | 254 | 289 | 129 | 784 | 293 | 295 | 97 | T | 15 | 1.51(1.17;1.96) |  | 1.28(1.05;1.57) | 1.04(0.79;1.38) | 0.00 |
| rs17330649 | 699 | 209 | 470 | 174 | 599 | 232 | 480 | 157 | T | 35 | 1.28(1;1.63) |  | 1.28(1.02;1.6) | 1.08(0.85;1.39) | 0.03 |
| rs17342946 | 962 | 290 | 207 | 93 | 837 | 308 | 242 | 82 | C | 11 | 1.49(1.11;1.98) |  | 1.19(0.98;1.45) | 1.1(0.82;1.48) | 0.04 |
| rs17361495 | 359 | 99 | 810 | 284 | 312 | 135 | 767 | 255 | C | 16 | 1.22(0.93;1.6) |  | 1.51(1.11;2.07) | 1.14(0.87;1.5) | 0.03 |
| rs17429654 | 763 | 229 | 405 | 153 | 700 | 263 | 378 | 126 | T | 20 | 1.26(0.98;1.61) |  | 1.26(1.02;1.56) | 1.02(0.79;1.33) | 0.02 |
| rs17571707 | 205 | 55 | 964 | 328 | 158 | 77 | 921 | 313 | C | 9 | 1.21(0.87;1.69) |  | 1.87(1.23;2.84) | 1.17(0.84;1.64) | 0.03 |
| rs2061456 | 576 | 178 | 593 | 205 | 505 | 208 | 574 | 182 | A | 28 | 1.22(0.96;1.55) |  | 1.34(1.05;1.7) | 1.07(0.84;1.37) | 0.03 |
| rs219467 | 166 | 47 | 913 | 343 | 188 | 83 | 981 | 300 | A | 40 | 1.3(0.91;1.87) |  | 1.61(1.05;2.48) | 1.08(0.75;1.56) | 0.04 |
| rs2306248 | 694 | 210 | 475 | 173 | 644 | 244 | 435 | 146 | A | 37 | 1.31(1.03;1.67) |  | 1.28(1.02;1.6) | 1.11(0.87;1.43) | 0.04 |
| rs2388993 | 378 | 91 | 701 | 299 | 352 | 114 | 817 | 269 | T | 45 | 1.75(1.33;2.3) |  | 1.3(0.94;1.8) | 1.41(1.07;1.87) | 0.03 |
| rs2388996 | 378 | 91 | 701 | 299 | 345 | 112 | 824 | 271 | T | 45 | 1.75(1.33;2.3) |  | 1.31(0.95;1.82) | 1.41(1.07;1.86) | 0.03 |
| rs2575584 | 365 | 101 | 696 | 283 | 384 | 136 | 763 | 237 | G | 43 | 1.43(1.1;1.87) |  | 1.25(0.93;1.7) | 1.14(0.87;1.5) | 0.04 |
| rs260903 | 322 | 93 | 757 | 297 | 316 | 116 | 853 | 267 | A | 15 | 1.44(1.09;1.9) |  | 1.36(0.98;1.88) | 1.16(0.87;1.53) | 0.02 |
| rs317873 | 710 | 201 | 459 | 182 | 627 | 247 | 452 | 143 | G | 23 | 1.38(1.09;1.76) |  | 1.38(1.11;1.73) | 1.06(0.82;1.36) | 0.00 |
| rs34526488 | 374 | 104 | 795 | 279 | 323 | 131 | 756 | 259 | T | 45 | 1.32(1.01;1.72) |  | 1.54(1.13;2.1) | 1.22(0.93;1.59) | 0.02 |
| rs3775552 | 279 | 82 | 800 | 308 | 235 | 93 | 934 | 290 | A | 12 | 1.27(0.96;1.69) |  | 1.41(0.99;2.01) | 1.04(0.78;1.39) | 0.03 |
| rs4253431 | 846 | 256 | 323 | 127 | 760 | 286 | 318 | 104 | A | 16 | 1.32(1.02;1.7) |  | 1.23(1.01;1.51) | 1.04(0.79;1.36) | 0.03 |
| rs4397044 | 625 | 193 | 454 | 197 | 640 | 212 | 529 | 171 | T | 25 | 1.46(1.15;1.86) |  | 1.11(0.88;1.41) | 1.1(0.86;1.4) | 0.03 |
| rs4446396 | 376 | 108 | 703 | 282 | 362 | 132 | 807 | 251 | A | 43 | 1.41(1.08;1.84) |  | 1.28(0.94;1.74) | 1.13(0.86;1.48) | 0.03 |
| rs4532251 | 579 | 169 | 500 | 221 | 603 | 195 | 566 | 188 | T | 28 | 1.5(1.18;1.91) |  | 1.14(0.89;1.46) | 1.15(0.9;1.47) | 0.03 |
| rs4691833 | 286 | 67 | 882 | 316 | 241 | 106 | 837 | 284 | G | 48 | 1.45(1.07;1.96) |  | 1.78(1.24;2.56) | 1.35(0.99;1.84) | 0.02 |
| rs477565 | 681 | 201 | 488 | 182 | 595 | 235 | 484 | 155 | T | 34 | 1.21(0.95;1.54) |  | 1.3(1.04;1.64) | 1.02(0.8;1.31) | 0.02 |
| rs4833282 | 461 | 141 | 707 | 242 | 401 | 172 | 678 | 218 | G | 22 | 1.17(0.91;1.5) |  | 1.42(1.09;1.86) | 1.05(0.82;1.35) | 0.02 |
| rs550658 | 674 | 200 | 494 | 183 | 587 | 234 | 492 | 156 | C | 33 | 1.2(0.94;1.53) |  | 1.31(1.04;1.64) | 1.01(0.79;1.3) | 0.02 |
| rs6535856 | 289 | 86 | 789 | 304 | 295 | 116 | 873 | 267 | A | 50 | 1.3(0.98;1.73) |  | 1.44(1.03;2.02) | 1.03(0.77;1.37) | 0.02 |
| rs6552438 | 585 | 176 | 584 | 207 | 483 | 207 | 596 | 183 | A | 28 | 1.2(0.94;1.52) |  | 1.41(1.1;1.8) | 1.01(0.79;1.29) | 0.01 |
| rs6849073 | 497 | 138 | 672 | 245 | 446 | 182 | 632 | 208 | G | 24 | 1.3(1.01;1.66) |  | 1.41(1.08;1.84) | 1.16(0.9;1.5) | 0.02 |
| rs6851590 | 203 | 63 | 966 | 320 | 156 | 90 | 923 | 299 | C | 9 | 1.06(0.77;1.46) |  | 1.74(1.17;2.59) | 1.02(0.74;1.41) | 0.04 |
| rs7667562 | 629 | 176 | 521 | 200 | 572 | 227 | 497 | 159 | A | 26 | 1.3(1.02;1.66) |  | 1.37(1.09;1.74) | 1.06(0.82;1.36) | 0.01 |
| rs7676088 | 376 | 91 | 703 | 299 | 345 | 112 | 823 | 271 | T | 45 | 1.73(1.32;2.28) |  | 1.31(0.94;1.81) | 1.4(1.06;1.85) | 0.03 |
| rs7693247 | 394 | 122 | 775 | 261 | 350 | 154 | 729 | 236 | G | 19 | 1.11(0.86;1.44) |  | 1.44(1.08;1.92) | 1.03(0.79;1.33) | 0.03 |
| rs7693601 | 575 | 153 | 593 | 230 | 476 | 198 | 603 | 192 | T | 27 | 1.37(1.08;1.75) |  | 1.48(1.15;1.91) | 1.13(0.88;1.45) | 0.00 |
| rs7697278 | 376 | 107 | 793 | 276 | 319 | 131 | 760 | 259 | C | 45 | 1.27(0.97;1.65) |  | 1.49(1.1;2.03) | 1.18(0.9;1.55) | 0.03 |
| rs783919 | 296 | 88 | 783 | 302 | 295 | 112 | 874 | 271 | A | 14 | 1.4(1.05;1.86) |  | 1.38(0.98;1.93) | 1.13(0.85;1.5) | 0.03 |
| rs966650 | 504 | 141 | 665 | 242 | 445 | 177 | 634 | 213 | A | 35 | 1.37(1.07;1.75) |  | 1.46(1.12;1.91) | 1.2(0.93;1.54) | 0.01 |
| chr 5 |  |  |  |  |  |  |  |  |  |  |  |  |  |  |  |
| rs10040838 | 444 | 127 | 724 | 256 | 357 | 151 | 722 | 239 | T | 20 | 1.25(0.97;1.6) |  | 1.48(1.11;1.96) | 1.13(0.87;1.46) | 0.02 |
| rs10055155 | 922 | 278 | 246 | 105 | 834 | 316 | 245 | 74 | C | 12 | 1.53(1.16;2.01) |  | 1.23(1.01;1.5) | 1.06(0.79;1.43) | 0.01 |
| rs10058955 | 501 | 144 | 577 | 246 | 525 | 185 | 644 | 198 | A | 33 | 1.5(1.17;1.91) |  | 1.3(1;1.68) | 1.07(0.83;1.38) | 0.00 |
| rs10069932 | 286 | 79 | 883 | 304 | 258 | 115 | 821 | 275 | C | 13 | 1.34(1;1.79) |  | 1.69(1.2;2.39) | 1.25(0.93;1.68) | 0.02 |
| rs10075145 | 453 | 127 | 716 | 256 | 387 | 157 | 692 | 233 | T | 39 | 1.24(0.96;1.59) |  | 1.4(1.06;1.85) | 1.15(0.89;1.48) | 0.04 |
| rs10078211 | 346 | 93 | 823 | 290 | 298 | 138 | 781 | 252 | T | 16 | 1.3(0.99;1.72) |  | 1.71(1.24;2.35) | 1.16(0.88;1.54) | 0.01 |
| rs10078903 | 466 | 143 | 703 | 240 | 440 | 184 | 639 | 206 | A | 24 | 1.18(0.92;1.51) |  | 1.38(1.06;1.79) | 1.07(0.83;1.38) | 0.04 |
| rs1035467 | 122 | 31 | 957 | 358 | 99 | 54 | 1070 | 329 | T | 32 | 1.7(1.11;2.62) |  | 2.44(1.43;4.19) | 1.41(0.92;2.18) | 0.02 |
| rs10472018 | 501 | 150 | 668 | 233 | 458 | 195 | 621 | 195 | G | 25 | 1.22(0.96;1.56) |  | 1.43(1.1;1.85) | 1.06(0.82;1.36) | 0.01 |
| rs10512632 | 709 | 209 | 370 | 181 | 750 | 258 | 419 | 125 | A | 20 | 1.65(1.29;2.1) |  | 1.2(0.97;1.5) | 1.01(0.78;1.31) | 0.00 |
| rs11740524 | 457 | 134 | 712 | 249 | 421 | 177 | 658 | 213 | C | 22 | 1.23(0.96;1.58) |  | 1.41(1.08;1.84) | 1.11(0.86;1.43) | 0.03 |
| rs11745439 | 600 | 184 | 479 | 206 | 558 | 193 | 611 | 190 | A | 29 | 1.34(1.05;1.7) |  | 1.13(0.89;1.44) | 1.01(0.8;1.29) | 0.04 |
| rs11956371 | 458 | 130 | 711 | 253 | 375 | 163 | 704 | 227 | G | 21 | 1.28(1;1.65) |  | 1.47(1.11;1.94) | 1.15(0.89;1.49) | 0.02 |
| rs12186726 | 265 | 67 | 904 | 316 | 226 | 92 | 853 | 298 | C | 46 | 1.48(1.08;2.01) |  | 1.69(1.16;2.46) | 1.42(1.04;1.94) | 0.04 |
| rs12515836 | 527 | 142 | 641 | 241 | 485 | 187 | 594 | 203 | G | 33 | 1.38(1.08;1.76) |  | 1.37(1.05;1.77) | 1.25(0.97;1.61) | 0.04 |
| rs12518745 | 507 | 152 | 661 | 231 | 462 | 196 | 617 | 194 | C | 25 | 1.22(0.95;1.56) |  | 1.42(1.09;1.83) | 1.06(0.82;1.36) | 0.01 |
| rs12654099 | 862 | 252 | 307 | 131 | 765 | 284 | 314 | 106 | T | 15 | 1.43(1.11;1.86) |  | 1.26(1.03;1.54) | 1.07(0.82;1.41) | 0.01 |
| rs12697864 | 563 | 165 | 605 | 218 | 559 | 222 | 520 | 168 | G | 29 | 1.19(0.94;1.52) |  | 1.32(1.03;1.68) | 1.05(0.81;1.35) | 0.04 |
| rs13177101 | 323 | 79 | 846 | 304 | 266 | 111 | 813 | 279 | T | 49 | 1.38(1.03;1.83) |  | 1.63(1.16;2.3) | 1.29(0.97;1.73) | 0.03 |
| rs13183458 | 640 | 189 | 527 | 193 | 594 | 230 | 483 | 160 | T | 26 | 1.27(1;1.62) |  | 1.32(1.05;1.66) | 1.09(0.85;1.4) | 0.03 |
| rs13189802 | 111 | 25 | 968 | 365 | 86 | 42 | 1083 | 341 | T | 29 | 1.63(1.03;2.58) |  | 2.32(1.29;4.18) | 1.38(0.87;2.19) | 0.04 |
| rs1561734 | 487 | 152 | 682 | 231 | 428 | 190 | 651 | 200 | A | 24 | 1.13(0.88;1.44) |  | 1.38(1.06;1.79) | 1.01(0.79;1.29) | 0.03 |
| rs156436 | 383 | 117 | 696 | 272 | 405 | 145 | 763 | 238 | G | 41 | 1.34(1.04;1.74) |  | 1.24(0.93;1.66) | 1.08(0.83;1.4) | 0.04 |
| rs158487 | 571 | 172 | 508 | 218 | 578 | 195 | 591 | 188 | A | 29 | 1.39(1.09;1.77) |  | 1.16(0.91;1.49) | 1.04(0.81;1.32) | 0.02 |
| rs1588265 | 111 | 25 | 968 | 365 | 89 | 43 | 1080 | 340 | A | 30 | 1.63(1.03;2.58) |  | 2.29(1.28;4.1) | 1.38(0.87;2.19) | 0.04 |
| rs161086 | 1043 | 320 | 126 | 63 | 956 | 351 | 123 | 39 | T | 6 | 1.65(1.17;2.32) |  | 1.17(0.97;1.4) | 1.05(0.71;1.56) | 0.03 |
| rs164577 | 194 | 45 | 975 | 338 | 158 | 73 | 921 | 317 | A | 40 | 1.48(1.03;2.11) |  | 1.9(1.22;2.96) | 1.45(1.01;2.08) | 0.04 |
| rs17109268 | 157 | 39 | 922 | 351 | 137 | 65 | 1032 | 318 | G | 7 | 1.57(1.07;2.29) |  | 1.85(1.15;2.96) | 1.31(0.89;1.91) | 0.03 |
| rs17208683 | 376 | 103 | 792 | 279 | 343 | 135 | 736 | 255 | G | 44 | 1.3(1;1.7) |  | 1.48(1.09;2.02) | 1.23(0.94;1.61) | 0.04 |
| rs17223360 | 818 | 264 | 260 | 125 | 848 | 279 | 319 | 103 | C | 14 | 1.51(1.16;1.96) |  | 1.05(0.86;1.29) | 1.02(0.78;1.33) | 0.03 |
| rs17490659 | 898 | 263 | 271 | 120 | 792 | 284 | 287 | 106 | C | 14 | 1.62(1.24;2.11) |  | 1.25(1.02;1.53) | 1.18(0.9;1.54) | 0.01 |
| rs17608701 | 1083 | 339 | 86 | 44 | 990 | 361 | 89 | 29 | G | 29 | 1.78(1.2;2.64) |  | 1.15(0.96;1.38) | 1(0.64;1.57) | 0.03 |
| rs177077 | 507 | 156 | 661 | 227 | 413 | 178 | 666 | 212 | T | 24 | 1.12(0.88;1.43) |  | 1.39(1.07;1.81) | 1(0.79;1.28) | 0.03 |
| rs1799594 | 720 | 215 | 449 | 168 | 660 | 253 | 419 | 137 | T | 22 | 1.27(1;1.62) |  | 1.26(1.01;1.57) | 1.08(0.84;1.4) | 0.04 |
| rs2053028 | 645 | 180 | 524 | 203 | 582 | 221 | 497 | 169 | A | 27 | 1.47(1.16;1.88) |  | 1.35(1.07;1.71) | 1.25(0.97;1.6) | 0.02 |
| rs2246473 | 686 | 194 | 478 | 189 | 599 | 222 | 478 | 167 | C | 25 | 1.4(1.1;1.78) |  | 1.28(1.02;1.62) | 1.2(0.94;1.53) | 0.04 |
| rs2303127 | 465 | 127 | 704 | 256 | 368 | 149 | 711 | 241 | C | 39 | 1.32(1.03;1.7) |  | 1.46(1.1;1.94) | 1.2(0.93;1.55) | 0.02 |
| rs2407163 | 568 | 178 | 511 | 212 | 609 | 204 | 560 | 179 | G | 28 | 1.44(1.13;1.83) |  | 1.14(0.9;1.46) | 1.08(0.84;1.38) | 0.03 |
| rs2408632 | 649 | 195 | 520 | 188 | 555 | 224 | 524 | 166 | C | 32 | 1.18(0.93;1.5) |  | 1.31(1.04;1.66) | 1.01(0.79;1.29) | 0.03 |
| rs245082 | 428 | 120 | 650 | 270 | 378 | 124 | 791 | 259 | G | 41 | 1.52(1.18;1.96) |  | 1.23(0.91;1.65) | 1.2(0.93;1.56) | 0.03 |
| rs255759 | 508 | 140 | 661 | 243 | 447 | 175 | 632 | 215 | G | 24 | 1.3(1.02;1.67) |  | 1.45(1.11;1.89) | 1.15(0.89;1.48) | 0.02 |
| rs2605897 | 590 | 173 | 579 | 209 | 520 | 206 | 559 | 184 | C | 30 | 1.22(0.96;1.55) |  | 1.32(1.03;1.68) | 1.09(0.85;1.4) | 0.04 |
| rs2625210 | 575 | 159 | 594 | 224 | 511 | 201 | 568 | 189 | T | 31 | 1.42(1.11;1.81) |  | 1.42(1.1;1.82) | 1.21(0.94;1.55) | 0.01 |
| rs2938772 | 493 | 132 | 676 | 251 | 428 | 173 | 651 | 217 | A | 36 | 1.42(1.11;1.83) |  | 1.54(1.18;2.02) | 1.21(0.94;1.57) | 0.01 |
| rs29454 | 614 | 182 | 555 | 201 | 547 | 218 | 532 | 172 | T | 29 | 1.31(1.03;1.67) |  | 1.33(1.05;1.68) | 1.14(0.89;1.45) | 0.03 |
| rs2963051 | 661 | 205 | 508 | 178 | 579 | 233 | 500 | 157 | C | 33 | 1.2(0.94;1.52) |  | 1.28(1.02;1.6) | 1.04(0.82;1.34) | 0.04 |
| rs36271 | 543 | 166 | 626 | 217 | 469 | 190 | 610 | 200 | A | 26 | 1.18(0.93;1.51) |  | 1.34(1.04;1.72) | 1.07(0.84;1.37) | 0.04 |
| rs3734046 | 644 | 176 | 525 | 207 | 577 | 219 | 502 | 171 | G | 27 | 1.53(1.21;1.95) |  | 1.38(1.09;1.75) | 1.28(0.99;1.64) | 0.01 |
| rs3804250 | 270 | 78 | 899 | 305 | 236 | 113 | 843 | 277 | C | 12 | 1.16(0.86;1.55) |  | 1.62(1.14;2.29) | 1.1(0.82;1.48) | 0.03 |
| rs3911664 | 285 | 74 | 883 | 309 | 240 | 106 | 839 | 284 | G | 48 | 1.36(1.01;1.83) |  | 1.69(1.19;2.42) | 1.28(0.95;1.73) | 0.02 |
| rs4262084 | 521 | 159 | 558 | 231 | 562 | 190 | 607 | 193 | G | 31 | 1.4(1.1;1.79) |  | 1.16(0.91;1.5) | 1.08(0.84;1.38) | 0.03 |
| rs428047 | 386 | 97 | 775 | 285 | 341 | 142 | 732 | 246 | C | 43 | 1.38(1.06;1.81) |  | 1.56(1.15;2.12) | 1.26(0.96;1.66) | 0.02 |
| rs4349736 | 233 | 69 | 846 | 321 | 241 | 93 | 928 | 290 | A | 11 | 1.35(0.99;1.83) |  | 1.46(1.01;2.12) | 1.11(0.81;1.51) | 0.03 |
| rs4382157 | 846 | 261 | 322 | 122 | 768 | 291 | 309 | 98 | T | 16 | 1.28(0.99;1.67) |  | 1.22(0.99;1.49) | 1.02(0.78;1.35) | 0.04 |
| rs4457052 | 521 | 157 | 558 | 233 | 562 | 189 | 607 | 194 | C | 31 | 1.43(1.12;1.83) |  | 1.17(0.91;1.51) | 1.1(0.85;1.41) | 0.03 |
| rs4507470 | 520 | 158 | 559 | 232 | 560 | 189 | 609 | 194 | C | 31 | 1.41(1.11;1.8) |  | 1.16(0.9;1.5) | 1.09(0.85;1.4) | 0.03 |
| rs4700355 | 110 | 25 | 969 | 365 | 85 | 43 | 1084 | 340 | A | 29 | 1.62(1.02;2.56) |  | 2.38(1.33;4.28) | 1.37(0.86;2.17) | 0.03 |
| rs4703019 | 420 | 101 | 749 | 282 | 368 | 153 | 711 | 237 | A | 41 | 1.46(1.12;1.9) |  | 1.61(1.19;2.16) | 1.3(0.99;1.7) | 0.01 |
| rs4704416 | 510 | 140 | 659 | 243 | 506 | 202 | 573 | 188 | C | 26 | 1.3(1.02;1.67) |  | 1.4(1.08;1.81) | 1.15(0.89;1.48) | 0.02 |
| rs4836280 | 395 | 112 | 773 | 271 | 332 | 141 | 747 | 249 | G | 43 | 1.23(0.95;1.59) |  | 1.46(1.08;1.96) | 1.14(0.88;1.49) | 0.04 |
| rs4866126 | 344 | 105 | 825 | 278 | 289 | 132 | 790 | 258 | A | 16 | 1.15(0.88;1.5) |  | 1.55(1.13;2.11) | 1.06(0.81;1.39) | 0.02 |
| rs4976574 | 524 | 161 | 645 | 222 | 456 | 188 | 623 | 202 | T | 25 | 1.18(0.93;1.51) |  | 1.36(1.06;1.76) | 1.06(0.83;1.35) | 0.03 |
| rs4998063 | 251 | 58 | 918 | 325 | 194 | 84 | 885 | 306 | C | 44 | 1.57(1.14;2.17) |  | 1.83(1.23;2.71) | 1.5(1.09;2.08) | 0.03 |
| rs581957 | 177 | 40 | 992 | 343 | 140 | 71 | 939 | 319 | C | 7 | 1.56(1.07;2.29) |  | 2.26(1.42;3.6) | 1.5(1.02;2.2) | 0.02 |
| rs6555225 | 397 | 123 | 772 | 260 | 361 | 152 | 718 | 238 | G | 19 | 1.16(0.9;1.5) |  | 1.43(1.07;1.91) | 1.07(0.83;1.4) | 0.04 |
| rs6872354 | 509 | 140 | 660 | 243 | 419 | 168 | 660 | 222 | C | 36 | 1.3(1.01;1.66) |  | 1.39(1.06;1.82) | 1.18(0.92;1.51) | 0.04 |
| rs6872795 | 522 | 154 | 557 | 236 | 550 | 190 | 619 | 193 | A | 32 | 1.4(1.1;1.79) |  | 1.22(0.95;1.57) | 1.03(0.8;1.33) | 0.01 |
| rs6894443 | 315 | 86 | 854 | 297 | 248 | 102 | 830 | 288 | G | 50 | 1.28(0.96;1.69) |  | 1.61(1.14;2.28) | 1.21(0.91;1.61) | 0.03 |
| rs6896503 | 225 | 69 | 854 | 321 | 208 | 86 | 961 | 297 | A | 44 | 1.23(0.9;1.67) |  | 1.45(0.99;2.12) | 1.02(0.75;1.39) | 0.04 |
| rs7444398 | 620 | 178 | 549 | 205 | 544 | 212 | 535 | 178 | A | 29 | 1.28(1.01;1.63) |  | 1.32(1.04;1.67) | 1.12(0.88;1.44) | 0.04 |
| rs7445806 | 388 | 102 | 780 | 281 | 328 | 133 | 751 | 257 | T | 44 | 1.45(1.11;1.9) |  | 1.53(1.12;2.08) | 1.35(1.03;1.77) | 0.03 |
| rs7708940 | 444 | 114 | 724 | 269 | 362 | 134 | 717 | 256 | G | 41 | 1.54(1.19;2) |  | 1.53(1.14;2.06) | 1.39(1.07;1.81) | 0.02 |
| rs7734168 | 110 | 25 | 969 | 365 | 83 | 43 | 1085 | 340 | C | 29 | 1.62(1.02;2.56) |  | 2.42(1.35;4.36) | 1.37(0.86;2.17) | 0.03 |
| rs874061 | 277 | 73 | 802 | 317 | 272 | 104 | 897 | 279 | A | 49 | 1.55(1.15;2.09) |  | 1.53(1.07;2.19) | 1.24(0.91;1.67) | 0.01 |
| rs878196 | 277 | 82 | 799 | 308 | 266 | 113 | 900 | 268 | G | 49 | 1.28(0.96;1.71) |  | 1.44(1.02;2.02) | 1.02(0.76;1.36) | 0.02 |
| rs895304 | 346 | 101 | 823 | 282 | 267 | 122 | 812 | 268 | A | 15 | 1.17(0.89;1.53) |  | 1.56(1.13;2.14) | 1.09(0.83;1.44) | 0.03 |
| rs924623 | 141 | 28 | 938 | 362 | 124 | 46 | 1045 | 337 | T | 33 | 2.2(1.41;3.43) |  | 2.02(1.16;3.52) | 1.87(1.2;2.92) | 0.04 |
| chr 6 |  |  |  |  |  |  |  |  |  |  |  |  |  |  |  |
| rs1016835 | 742 | 216 | 427 | 167 | 660 | 250 | 419 | 140 | G | 22 | 1.31(1.03;1.67) |  | 1.28(1.03;1.6) | 1.08(0.83;1.39) | 0.02 |
| rs1033500 | 405 | 113 | 674 | 277 | 424 | 156 | 745 | 227 | A | 41 | 1.58(1.22;2.05) |  | 1.38(1.03;1.84) | 1.19(0.91;1.56) | 0.01 |
| rs10455655 | 223 | 65 | 856 | 325 | 207 | 85 | 962 | 298 | A | 10 | 1.37(1;1.88) |  | 1.56(1.06;2.31) | 1.12(0.82;1.54) | 0.02 |
| rs10456082 | 852 | 254 | 317 | 129 | 772 | 286 | 307 | 104 | G | 15 | 1.3(1.01;1.69) |  | 1.22(1;1.49) | 1.04(0.79;1.37) | 0.04 |
| rs10484820 | 770 | 227 | 399 | 156 | 659 | 251 | 420 | 139 | G | 21 | 1.29(1.01;1.65) |  | 1.28(1.03;1.58) | 1.05(0.82;1.35) | 0.02 |
| rs1075496 | 184 | 51 | 895 | 339 | 183 | 78 | 986 | 305 | C | 40 | 1.4(0.99;1.97) |  | 1.62(1.06;2.47) | 1.16(0.82;1.64) | 0.03 |
| rs1076712 | 420 | 123 | 749 | 260 | 386 | 160 | 693 | 230 | C | 41 | 1.19(0.92;1.54) |  | 1.44(1.09;1.92) | 1.09(0.84;1.41) | 0.03 |
| rs10807100 | 404 | 113 | 675 | 277 | 423 | 156 | 746 | 227 | A | 41 | 1.57(1.21;2.04) |  | 1.38(1.03;1.84) | 1.19(0.91;1.55) | 0.01 |
| rs10947698 | 229 | 43 | 940 | 340 | 179 | 73 | 900 | 317 | G | 41 | 1.79(1.25;2.56) |  | 2.03(1.31;3.14) | 1.73(1.2;2.48) | 0.03 |
| rs11153303 | 273 | 71 | 806 | 319 | 302 | 107 | 867 | 276 | G | 50 | 1.49(1.1;2.01) |  | 1.36(0.96;1.93) | 1.23(0.91;1.67) | 0.04 |
| rs11154755 | 409 | 126 | 670 | 264 | 458 | 162 | 711 | 221 | T | 38 | 1.28(0.99;1.64) |  | 1.2(0.91;1.59) | 1.01(0.78;1.31) | 0.04 |
| rs1160571 | 594 | 172 | 575 | 210 | 531 | 208 | 548 | 182 | C | 29 | 1.26(0.99;1.6) |  | 1.32(1.04;1.69) | 1.12(0.87;1.43) | 0.04 |
| rs12192468 | 149 | 37 | 930 | 353 | 129 | 61 | 1040 | 322 | C | 7 | 1.55(1.05;2.28) |  | 2.04(1.26;3.31) | 1.28(0.87;1.89) | 0.02 |
| rs12196381 | 272 | 71 | 806 | 319 | 302 | 107 | 867 | 276 | G | 50 | 1.48(1.1;2) |  | 1.35(0.95;1.92) | 1.23(0.91;1.66) | 0.04 |
| rs12208931 | 337 | 91 | 832 | 292 | 292 | 124 | 787 | 266 | C | 15 | 1.32(1;1.75) |  | 1.6(1.16;2.22) | 1.22(0.92;1.62) | 0.02 |
| rs1259078 | 250 | 72 | 829 | 318 | 231 | 96 | 938 | 287 | T | 46 | 1.34(0.99;1.81) |  | 1.48(1.03;2.14) | 1.09(0.81;1.48) | 0.02 |
| rs12663048 | 432 | 132 | 734 | 251 | 404 | 170 | 673 | 218 | C | 21 | 1.16(0.91;1.5) |  | 1.39(1.06;1.84) | 1.06(0.82;1.37) | 0.03 |
| rs1267488 | 341 | 92 | 828 | 291 | 297 | 129 | 782 | 261 | G | 16 | 1.21(0.92;1.6) |  | 1.5(1.09;2.07) | 1.15(0.87;1.52) | 0.04 |
| rs1267495 | 397 | 110 | 772 | 273 | 341 | 149 | 738 | 241 | C | 19 | 1.2(0.92;1.56) |  | 1.48(1.1;1.98) | 1.11(0.85;1.44) | 0.03 |
| rs1267499 | 396 | 110 | 773 | 273 | 340 | 148 | 739 | 242 | T | 19 | 1.2(0.92;1.55) |  | 1.47(1.09;1.97) | 1.11(0.85;1.45) | 0.03 |
| rs1267501 | 395 | 110 | 774 | 273 | 340 | 148 | 739 | 242 | T | 19 | 1.19(0.92;1.55) |  | 1.46(1.09;1.97) | 1.1(0.85;1.44) | 0.03 |
| rs1267505 | 409 | 115 | 760 | 268 | 343 | 149 | 736 | 241 | C | 19 | 1.19(0.92;1.54) |  | 1.46(1.09;1.96) | 1.1(0.85;1.43) | 0.03 |
| rs1268065 | 261 | 76 | 799 | 309 | 238 | 102 | 897 | 269 | G | 49 | 1.3(0.96;1.74) |  | 1.48(1.04;2.12) | 1.02(0.76;1.38) | 0.02 |
| rs13192060 | 318 | 88 | 832 | 292 | 284 | 121 | 787 | 266 | A | 15 | 1.32(0.99;1.75) |  | 1.61(1.16;2.23) | 1.22(0.91;1.62) | 0.02 |
| rs13194827 | 449 | 120 | 720 | 263 | 384 | 164 | 695 | 226 | G | 20 | 1.42(1.1;1.83) |  | 1.6(1.2;2.12) | 1.23(0.95;1.6) | 0.01 |
| rs13195945 | 316 | 90 | 831 | 291 | 276 | 118 | 784 | 266 | A | 15 | 1.27(0.96;1.68) |  | 1.53(1.1;2.12) | 1.18(0.89;1.57) | 0.03 |
| rs13196204 | 386 | 110 | 783 | 273 | 314 | 134 | 765 | 256 | G | 17 | 1.26(0.97;1.63) |  | 1.53(1.13;2.07) | 1.16(0.89;1.5) | 0.02 |
| rs13203718 | 422 | 107 | 747 | 276 | 358 | 140 | 721 | 250 | G | 42 | 1.42(1.1;1.85) |  | 1.55(1.15;2.09) | 1.29(0.99;1.68) | 0.02 |
| rs1328870 | 268 | 77 | 811 | 313 | 298 | 108 | 871 | 275 | A | 50 | 1.36(1.01;1.82) |  | 1.34(0.95;1.9) | 1.12(0.83;1.51) | 0.04 |
| rs1342196 | 468 | 139 | 701 | 244 | 427 | 182 | 652 | 208 | A | 23 | 1.12(0.87;1.43) |  | 1.37(1.05;1.79) | 1.02(0.79;1.31) | 0.04 |
| rs1474729 | 404 | 113 | 675 | 277 | 423 | 156 | 746 | 227 | C | 41 | 1.57(1.21;2.04) |  | 1.38(1.03;1.84) | 1.19(0.91;1.55) | 0.01 |
| rs1559876 | 432 | 126 | 646 | 264 | 452 | 163 | 716 | 220 | C | 39 | 1.52(1.18;1.96) |  | 1.31(0.99;1.73) | 1.16(0.89;1.5) | 0.01 |
| rs17119 | 401 | 110 | 768 | 273 | 351 | 152 | 728 | 238 | G | 19 | 1.21(0.93;1.57) |  | 1.47(1.09;1.97) | 1.11(0.85;1.45) | 0.03 |
| rs171836 | 683 | 207 | 486 | 176 | 646 | 247 | 433 | 143 | C | 24 | 1.3(1.02;1.66) |  | 1.28(1.03;1.6) | 1.1(0.85;1.42) | 0.03 |
| rs171895 | 227 | 54 | 942 | 329 | 155 | 72 | 924 | 318 | A | 40 | 1.47(1.06;2.05) |  | 1.88(1.23;2.87) | 1.43(1.02;1.99) | 0.03 |
| rs174372 | 431 | 131 | 738 | 252 | 402 | 168 | 677 | 222 | A | 21 | 1.18(0.91;1.51) |  | 1.39(1.06;1.84) | 1.08(0.84;1.4) | 0.04 |
| rs174373 | 455 | 136 | 714 | 247 | 420 | 173 | 659 | 217 | T | 22 | 1.2(0.94;1.54) |  | 1.39(1.06;1.83) | 1.1(0.85;1.42) | 0.04 |
| rs174379 | 431 | 131 | 738 | 252 | 402 | 168 | 677 | 222 | T | 21 | 1.18(0.91;1.51) |  | 1.39(1.06;1.84) | 1.08(0.84;1.4) | 0.04 |
| rs174380 | 431 | 132 | 738 | 251 | 401 | 168 | 678 | 222 | T | 21 | 1.17(0.91;1.5) |  | 1.39(1.05;1.83) | 1.07(0.83;1.39) | 0.04 |
| rs17539197 | 318 | 89 | 760 | 299 | 342 | 127 | 823 | 256 | C | 46 | 1.39(1.05;1.83) |  | 1.33(0.96;1.83) | 1.13(0.85;1.5) | 0.03 |
| rs17576984 | 243 | 76 | 926 | 307 | 223 | 108 | 856 | 282 | T | 12 | 1.18(0.87;1.6) |  | 1.62(1.13;2.32) | 1.13(0.83;1.53) | 0.04 |
| rs1766530 | 522 | 159 | 557 | 231 | 512 | 171 | 657 | 212 | G | 33 | 1.4(1.1;1.78) |  | 1.17(0.9;1.51) | 1.07(0.84;1.37) | 0.03 |
| rs1974226 | 419 | 117 | 750 | 266 | 323 | 150 | 756 | 240 | T | 19 | 1.26(0.98;1.63) |  | 1.63(1.22;2.18) | 1.1(0.85;1.43) | 0.01 |
| rs2022533 | 390 | 109 | 689 | 281 | 407 | 150 | 762 | 233 | G | 42 | 1.56(1.2;2.03) |  | 1.36(1.02;1.83) | 1.2(0.91;1.57) | 0.01 |
| rs2022534 | 405 | 113 | 674 | 277 | 424 | 156 | 745 | 227 | C | 41 | 1.58(1.22;2.05) |  | 1.38(1.03;1.84) | 1.19(0.91;1.56) | 0.01 |
| rs2024831 | 387 | 107 | 782 | 276 | 328 | 146 | 751 | 244 | A | 18 | 1.21(0.93;1.57) |  | 1.53(1.13;2.06) | 1.1(0.84;1.44) | 0.02 |
| rs2073046 | 405 | 113 | 674 | 277 | 424 | 156 | 745 | 227 | T | 41 | 1.58(1.22;2.05) |  | 1.38(1.03;1.84) | 1.19(0.91;1.56) | 0.01 |
| rs2075965 | 359 | 106 | 810 | 277 | 323 | 143 | 756 | 247 | T | 17 | 1.18(0.91;1.54) |  | 1.48(1.09;2) | 1.1(0.84;1.44) | 0.03 |
| rs2075966 | 359 | 107 | 810 | 276 | 323 | 143 | 756 | 247 | G | 17 | 1.16(0.89;1.52) |  | 1.46(1.08;1.98) | 1.09(0.83;1.42) | 0.04 |
| rs2076538 | 405 | 113 | 674 | 277 | 424 | 156 | 745 | 227 | C | 41 | 1.58(1.22;2.05) |  | 1.38(1.03;1.84) | 1.19(0.91;1.56) | 0.01 |
| rs2076540 | 417 | 119 | 662 | 271 | 434 | 157 | 735 | 226 | C | 40 | 1.52(1.18;1.97) |  | 1.32(0.99;1.75) | 1.17(0.9;1.52) | 0.01 |
| rs2076541 | 405 | 113 | 674 | 277 | 425 | 156 | 744 | 227 | C | 41 | 1.58(1.22;2.05) |  | 1.38(1.03;1.84) | 1.19(0.91;1.56) | 0.01 |
| rs2076542 | 405 | 113 | 674 | 277 | 424 | 156 | 744 | 227 | A | 41 | 1.58(1.22;2.05) |  | 1.38(1.03;1.84) | 1.19(0.91;1.56) | 0.01 |
| rs2143465 | 405 | 113 | 674 | 277 | 424 | 156 | 745 | 227 | C | 41 | 1.58(1.22;2.05) |  | 1.38(1.03;1.84) | 1.19(0.91;1.56) | 0.01 |
| rs2143466 | 400 | 106 | 679 | 284 | 408 | 154 | 761 | 229 | T | 41 | 1.71(1.31;2.22) |  | 1.5(1.11;2.01) | 1.25(0.95;1.64) | 0.00 |
| rs2143468 | 405 | 113 | 674 | 277 | 424 | 156 | 745 | 227 | T | 41 | 1.58(1.22;2.05) |  | 1.38(1.03;1.84) | 1.19(0.91;1.56) | 0.01 |
| rs2185509 | 1000 | 304 | 168 | 79 | 898 | 328 | 181 | 62 | T | 9 | 1.66(1.22;2.27) |  | 1.18(0.98;1.43) | 1.13(0.82;1.57) | 0.03 |
| rs2206618 | 625 | 184 | 526 | 193 | 584 | 222 | 481 | 165 | G | 27 | 1.29(1.01;1.64) |  | 1.31(1.03;1.65) | 1.13(0.88;1.45) | 0.04 |
| rs222541 | 670 | 202 | 499 | 181 | 598 | 242 | 481 | 148 | C | 34 | 1.24(0.98;1.58) |  | 1.34(1.07;1.68) | 1.01(0.79;1.3) | 0.01 |
| rs2395163 | 366 | 106 | 803 | 277 | 356 | 146 | 723 | 244 | C | 17 | 1.25(0.96;1.63) |  | 1.48(1.09;1.99) | 1.16(0.89;1.52) | 0.03 |
| rs2498588 | 795 | 228 | 374 | 155 | 729 | 267 | 350 | 123 | T | 18 | 1.47(1.15;1.88) |  | 1.26(1.02;1.55) | 1.2(0.93;1.56) | 0.03 |
| rs270413 | 290 | 74 | 879 | 309 | 264 | 124 | 815 | 266 | T | 50 | 1.53(1.13;2.06) |  | 1.9(1.34;2.69) | 1.37(1.01;1.86) | 0.01 |
| rs2738822 | 436 | 128 | 643 | 262 | 404 | 143 | 765 | 240 | C | 40 | 1.39(1.08;1.78) |  | 1.24(0.94;1.65) | 1.08(0.84;1.4) | 0.02 |
| rs28732121 | 335 | 92 | 827 | 290 | 292 | 124 | 782 | 265 | G | 15 | 1.3(0.98;1.71) |  | 1.57(1.14;2.17) | 1.2(0.91;1.59) | 0.02 |
| rs28895002 | 337 | 91 | 832 | 292 | 292 | 124 | 787 | 266 | A | 15 | 1.32(1;1.75) |  | 1.6(1.16;2.22) | 1.22(0.92;1.62) | 0.02 |
| rs28895007 | 337 | 91 | 832 | 292 | 292 | 124 | 787 | 266 | C | 15 | 1.32(1;1.75) |  | 1.6(1.16;2.22) | 1.22(0.92;1.62) | 0.02 |
| rs2894176 | 371 | 111 | 708 | 279 | 373 | 141 | 794 | 242 | C | 43 | 1.3(1;1.68) |  | 1.24(0.92;1.67) | 1.04(0.8;1.36) | 0.04 |
| rs3777913 | 432 | 131 | 737 | 252 | 402 | 168 | 677 | 222 | C | 21 | 1.18(0.92;1.52) |  | 1.4(1.06;1.84) | 1.08(0.84;1.4) | 0.04 |
| rs3800043 | 660 | 202 | 509 | 181 | 592 | 232 | 487 | 158 | T | 34 | 1.19(0.93;1.51) |  | 1.3(1.03;1.63) | 1.01(0.79;1.3) | 0.03 |
| rs3827783 | 841 | 245 | 328 | 138 | 709 | 270 | 370 | 120 | G | 17 | 1.46(1.14;1.89) |  | 1.32(1.07;1.62) | 1.05(0.81;1.36) | 0.00 |
| rs3916765 | 239 | 63 | 930 | 320 | 237 | 104 | 842 | 286 | A | 11 | 1.33(0.97;1.82) |  | 1.66(1.15;2.41) | 1.27(0.93;1.75) | 0.04 |
| rs4318902 | 940 | 284 | 229 | 99 | 867 | 326 | 211 | 64 | G | 45 | 1.43(1.08;1.89) |  | 1.21(1;1.46) | 1.01(0.74;1.4) | 0.02 |
| rs4446534 | 407 | 121 | 672 | 269 | 415 | 155 | 753 | 228 | A | 39 | 1.35(1.04;1.74) |  | 1.28(0.96;1.7) | 1.05(0.81;1.36) | 0.02 |
| rs459970 | 922 | 268 | 247 | 115 | 842 | 308 | 237 | 82 | A | 12 | 1.6(1.22;2.1) |  | 1.24(1.02;1.51) | 1.14(0.84;1.53) | 0.01 |
| rs4642516 | 298 | 83 | 869 | 300 | 253 | 110 | 825 | 280 | G | 49 | 1.22(0.92;1.62) |  | 1.56(1.11;2.2) | 1.16(0.87;1.55) | 0.04 |
| rs4706767 | 764 | 226 | 405 | 157 | 682 | 259 | 397 | 131 | G | 20 | 1.32(1.03;1.69) |  | 1.26(1.01;1.56) | 1.1(0.85;1.42) | 0.04 |
| rs4713411 | 359 | 104 | 720 | 286 | 359 | 136 | 810 | 247 | A | 44 | 1.36(1.05;1.78) |  | 1.3(0.96;1.76) | 1.08(0.83;1.42) | 0.03 |
| rs4713518 | 403 | 114 | 676 | 276 | 422 | 156 | 747 | 227 | G | 41 | 1.55(1.19;2) |  | 1.36(1.02;1.82) | 1.17(0.9;1.53) | 0.01 |
| rs4714899 | 569 | 152 | 600 | 231 | 494 | 190 | 585 | 200 | A | 33 | 1.41(1.1;1.79) |  | 1.43(1.11;1.84) | 1.21(0.94;1.56) | 0.01 |
| rs477005 | 415 | 121 | 664 | 269 | 432 | 157 | 737 | 226 | G | 40 | 1.47(1.14;1.9) |  | 1.29(0.97;1.72) | 1.13(0.87;1.47) | 0.01 |
| rs482194 | 403 | 114 | 676 | 276 | 422 | 156 | 747 | 227 | G | 41 | 1.55(1.19;2) |  | 1.36(1.02;1.82) | 1.17(0.9;1.53) | 0.01 |
| rs485774 | 404 | 114 | 675 | 276 | 422 | 156 | 747 | 227 | G | 41 | 1.55(1.2;2.01) |  | 1.37(1.02;1.83) | 1.17(0.9;1.53) | 0.01 |
| rs4959025 | 405 | 113 | 674 | 277 | 424 | 156 | 745 | 227 | C | 41 | 1.58(1.22;2.05) |  | 1.38(1.03;1.84) | 1.19(0.91;1.56) | 0.01 |
| rs4959026 | 405 | 113 | 674 | 277 | 424 | 156 | 745 | 227 | T | 41 | 1.58(1.22;2.05) |  | 1.38(1.03;1.84) | 1.19(0.91;1.56) | 0.01 |
| rs4959094 | 405 | 113 | 674 | 277 | 424 | 156 | 745 | 227 | T | 41 | 1.58(1.22;2.05) |  | 1.38(1.03;1.84) | 1.19(0.91;1.56) | 0.01 |
| rs4959096 | 405 | 113 | 674 | 277 | 425 | 156 | 744 | 227 | A | 41 | 1.58(1.22;2.05) |  | 1.38(1.03;1.84) | 1.19(0.91;1.56) | 0.01 |
| rs5000634 | 401 | 123 | 678 | 267 | 406 | 153 | 763 | 230 | G | 40 | 1.33(1.04;1.72) |  | 1.27(0.96;1.69) | 1.04(0.8;1.34) | 0.02 |
| rs502626 | 403 | 114 | 676 | 276 | 422 | 156 | 747 | 227 | G | 41 | 1.55(1.19;2) |  | 1.36(1.02;1.82) | 1.17(0.9;1.53) | 0.01 |
| rs504203 | 404 | 113 | 675 | 277 | 422 | 156 | 747 | 227 | C | 41 | 1.57(1.21;2.04) |  | 1.38(1.03;1.85) | 1.19(0.91;1.55) | 0.01 |
| rs505274 | 404 | 113 | 675 | 277 | 422 | 156 | 747 | 227 | A | 41 | 1.57(1.21;2.04) |  | 1.38(1.03;1.85) | 1.19(0.91;1.55) | 0.01 |
| rs508805 | 404 | 113 | 675 | 277 | 422 | 156 | 747 | 227 | A | 41 | 1.57(1.21;2.04) |  | 1.38(1.03;1.85) | 1.19(0.91;1.55) | 0.01 |
| rs523627 | 404 | 114 | 675 | 276 | 422 | 156 | 747 | 227 | T | 41 | 1.55(1.2;2.01) |  | 1.37(1.02;1.83) | 1.17(0.9;1.53) | 0.01 |
| rs524578 | 389 | 109 | 690 | 281 | 405 | 150 | 764 | 233 | A | 42 | 1.55(1.19;2.02) |  | 1.37(1.02;1.84) | 1.19(0.91;1.56) | 0.01 |
| rs525607 | 389 | 110 | 690 | 280 | 405 | 150 | 764 | 233 | T | 42 | 1.53(1.18;1.99) |  | 1.35(1.01;1.82) | 1.18(0.9;1.54) | 0.01 |
| rs531094 | 404 | 114 | 675 | 276 | 422 | 156 | 747 | 227 | A | 41 | 1.55(1.2;2.01) |  | 1.37(1.02;1.83) | 1.17(0.9;1.53) | 0.01 |
| rs537757 | 403 | 114 | 676 | 276 | 422 | 156 | 747 | 227 | T | 41 | 1.55(1.19;2) |  | 1.36(1.02;1.82) | 1.17(0.9;1.53) | 0.01 |
| rs539703 | 404 | 114 | 675 | 276 | 422 | 157 | 747 | 226 | C | 41 | 1.55(1.2;2.01) |  | 1.38(1.03;1.84) | 1.17(0.89;1.52) | 0.01 |
| rs546857 | 416 | 121 | 663 | 269 | 432 | 157 | 737 | 226 | T | 40 | 1.48(1.14;1.91) |  | 1.3(0.98;1.72) | 1.14(0.87;1.48) | 0.01 |
| rs547077 | 389 | 110 | 690 | 280 | 405 | 150 | 764 | 233 | C | 42 | 1.53(1.18;1.99) |  | 1.35(1.01;1.82) | 1.18(0.9;1.54) | 0.01 |
| rs547261 | 404 | 114 | 675 | 276 | 422 | 156 | 747 | 227 | A | 41 | 1.55(1.2;2.01) |  | 1.37(1.02;1.83) | 1.17(0.9;1.53) | 0.01 |
| rs552339 | 404 | 114 | 675 | 276 | 422 | 156 | 747 | 227 | A | 41 | 1.55(1.2;2.01) |  | 1.37(1.02;1.83) | 1.17(0.9;1.53) | 0.01 |
| rs557305 | 262 | 81 | 817 | 309 | 283 | 109 | 886 | 274 | T | 13 | 1.32(0.98;1.76) |  | 1.35(0.96;1.92) | 1.08(0.8;1.46) | 0.04 |
| rs560505 | 415 | 121 | 664 | 269 | 432 | 157 | 737 | 226 | G | 40 | 1.47(1.14;1.9) |  | 1.29(0.97;1.72) | 1.13(0.87;1.47) | 0.01 |
| rs584032 | 453 | 118 | 716 | 265 | 344 | 138 | 735 | 252 | A | 41 | 1.41(1.09;1.82) |  | 1.53(1.14;2.05) | 1.27(0.98;1.64) | 0.02 |
| rs598623 | 450 | 130 | 719 | 253 | 350 | 143 | 729 | 247 | C | 41 | 1.27(0.98;1.63) |  | 1.45(1.09;1.93) | 1.17(0.9;1.5) | 0.03 |
| rs610614 | 544 | 155 | 625 | 228 | 463 | 184 | 616 | 206 | C | 34 | 1.34(1.05;1.71) |  | 1.43(1.11;1.85) | 1.16(0.9;1.49) | 0.01 |
| rs611348 | 376 | 109 | 703 | 281 | 378 | 143 | 791 | 240 | A | 43 | 1.45(1.11;1.88) |  | 1.32(0.98;1.78) | 1.13(0.87;1.48) | 0.02 |
| rs6457509 | 368 | 113 | 706 | 275 | 388 | 141 | 778 | 240 | C | 44 | 1.39(1.07;1.8) |  | 1.23(0.91;1.66) | 1.11(0.85;1.45) | 0.04 |
| rs6457622 | 401 | 123 | 678 | 267 | 406 | 153 | 763 | 230 | C | 40 | 1.33(1.04;1.72) |  | 1.27(0.96;1.69) | 1.04(0.8;1.34) | 0.02 |
| rs6458555 | 637 | 181 | 532 | 202 | 547 | 217 | 532 | 173 | A | 31 | 1.3(1.02;1.65) |  | 1.37(1.09;1.74) | 1.07(0.84;1.38) | 0.01 |
| rs685449 | 450 | 130 | 718 | 251 | 351 | 143 | 728 | 247 | A | 41 | 1.26(0.98;1.62) |  | 1.45(1.08;1.93) | 1.16(0.9;1.5) | 0.03 |
| rs6902696 | 720 | 214 | 449 | 169 | 609 | 236 | 470 | 154 | G | 24 | 1.24(0.97;1.58) |  | 1.27(1.01;1.59) | 1.07(0.83;1.37) | 0.04 |
| rs6908205 | 337 | 91 | 832 | 292 | 292 | 124 | 787 | 266 | A | 15 | 1.32(1;1.75) |  | 1.6(1.16;2.22) | 1.22(0.92;1.62) | 0.02 |
| rs6926543 | 448 | 135 | 631 | 255 | 477 | 165 | 692 | 218 | G | 36 | 1.43(1.11;1.83) |  | 1.22(0.93;1.61) | 1.11(0.86;1.44) | 0.02 |
| rs6928482 | 337 | 99 | 742 | 291 | 330 | 136 | 839 | 247 | C | 45 | 1.37(1.05;1.79) |  | 1.47(1.07;2) | 1.04(0.79;1.37) | 0.01 |
| rs6929776 | 389 | 109 | 690 | 281 | 406 | 150 | 763 | 233 | A | 42 | 1.55(1.19;2.02) |  | 1.36(1.02;1.83) | 1.19(0.91;1.56) | 0.01 |
| rs6930681 | 404 | 113 | 675 | 277 | 423 | 156 | 746 | 227 | A | 41 | 1.57(1.21;2.04) |  | 1.38(1.03;1.84) | 1.19(0.91;1.55) | 0.01 |
| rs6937258 | 755 | 224 | 413 | 159 | 674 | 255 | 405 | 135 | T | 21 | 1.31(1.02;1.67) |  | 1.27(1.02;1.57) | 1.08(0.84;1.39) | 0.03 |
| rs761187 | 417 | 119 | 662 | 271 | 435 | 157 | 734 | 226 | G | 40 | 1.52(1.18;1.97) |  | 1.32(0.99;1.75) | 1.17(0.9;1.52) | 0.01 |
| rs7739550 | 279 | 81 | 758 | 301 | 272 | 101 | 842 | 271 | A | 50 | 1.38(1.03;1.84) |  | 1.34(0.95;1.91) | 1.13(0.84;1.51) | 0.04 |
| rs7744001 | 628 | 183 | 538 | 198 | 547 | 217 | 524 | 171 | A | 32 | 1.25(0.98;1.59) |  | 1.33(1.05;1.69) | 1.08(0.85;1.39) | 0.03 |
| rs7745040 | 400 | 121 | 679 | 269 | 406 | 152 | 763 | 231 | C | 40 | 1.36(1.05;1.76) |  | 1.28(0.96;1.71) | 1.06(0.82;1.37) | 0.02 |
| rs7755802 | 359 | 104 | 720 | 286 | 359 | 136 | 810 | 247 | G | 44 | 1.36(1.05;1.78) |  | 1.3(0.96;1.76) | 1.08(0.83;1.42) | 0.03 |
| rs7758976 | 359 | 104 | 720 | 286 | 359 | 136 | 810 | 247 | G | 44 | 1.36(1.05;1.78) |  | 1.3(0.96;1.76) | 1.08(0.83;1.42) | 0.03 |
| rs7771685 | 201 | 41 | 968 | 342 | 124 | 58 | 955 | 332 | T | 38 | 1.78(1.22;2.58) |  | 2.33(1.45;3.76) | 1.71(1.17;2.48) | 0.02 |
| rs7772702 | 263 | 69 | 812 | 319 | 283 | 100 | 882 | 280 | G | 49 | 1.48(1.09;2) |  | 1.36(0.95;1.95) | 1.22(0.9;1.66) | 0.04 |
| rs7772845 | 792 | 243 | 376 | 140 | 714 | 275 | 365 | 115 | T | 42 | 1.25(0.97;1.61) |  | 1.25(1.01;1.54) | 1.01(0.77;1.31) | 0.03 |
| rs7773756 | 215 | 54 | 954 | 329 | 169 | 74 | 910 | 316 | T | 41 | 1.41(1.01;1.98) |  | 1.8(1.19;2.73) | 1.38(0.98;1.93) | 0.04 |
| rs7775843 | 548 | 134 | 621 | 249 | 479 | 181 | 600 | 209 | C | 34 | 1.52(1.19;1.95) |  | 1.46(1.13;1.91) | 1.32(1.03;1.71) | 0.01 |
| rs910052 | 404 | 113 | 675 | 277 | 423 | 156 | 746 | 227 | T | 41 | 1.57(1.21;2.04) |  | 1.38(1.03;1.84) | 1.19(0.91;1.55) | 0.01 |
| rs9262499 | 359 | 104 | 720 | 286 | 359 | 136 | 810 | 247 | T | 44 | 1.36(1.05;1.78) |  | 1.3(0.96;1.76) | 1.08(0.83;1.42) | 0.03 |
| rs926594 | 405 | 113 | 674 | 277 | 423 | 156 | 746 | 227 | G | 41 | 1.58(1.22;2.05) |  | 1.38(1.03;1.85) | 1.19(0.91;1.56) | 0.01 |
| rs9267954 | 677 | 211 | 402 | 179 | 741 | 247 | 428 | 136 | T | 22 | 1.51(1.18;1.92) |  | 1.13(0.91;1.41) | 1.05(0.81;1.35) | 0.01 |
| rs9267956 | 430 | 126 | 649 | 264 | 450 | 163 | 719 | 220 | G | 39 | 1.5(1.16;1.93) |  | 1.3(0.98;1.72) | 1.14(0.88;1.48) | 0.01 |
| rs9268132 | 403 | 114 | 676 | 276 | 422 | 156 | 747 | 227 | G | 41 | 1.55(1.19;2) |  | 1.36(1.02;1.82) | 1.17(0.9;1.53) | 0.01 |
| rs9268260 | 405 | 113 | 674 | 277 | 424 | 156 | 745 | 227 | T | 41 | 1.58(1.22;2.05) |  | 1.38(1.03;1.84) | 1.19(0.91;1.56) | 0.01 |
| rs9268283 | 405 | 113 | 674 | 277 | 425 | 156 | 744 | 227 | C | 41 | 1.58(1.22;2.05) |  | 1.38(1.03;1.84) | 1.19(0.91;1.56) | 0.01 |
| rs9268284 | 405 | 113 | 674 | 277 | 425 | 156 | 744 | 227 | A | 41 | 1.58(1.22;2.05) |  | 1.38(1.03;1.84) | 1.19(0.91;1.56) | 0.01 |
| rs9268285 | 405 | 113 | 674 | 277 | 425 | 156 | 744 | 227 | C | 41 | 1.58(1.22;2.05) |  | 1.38(1.03;1.84) | 1.19(0.91;1.56) | 0.01 |
| rs9268326 | 405 | 113 | 674 | 277 | 424 | 156 | 745 | 227 | C | 41 | 1.58(1.22;2.05) |  | 1.38(1.03;1.84) | 1.19(0.91;1.56) | 0.01 |
| rs9268343 | 405 | 113 | 674 | 277 | 424 | 156 | 745 | 227 | G | 41 | 1.58(1.22;2.05) |  | 1.38(1.03;1.84) | 1.19(0.91;1.56) | 0.01 |
| rs9268345 | 405 | 113 | 674 | 277 | 424 | 156 | 745 | 227 | A | 41 | 1.58(1.22;2.05) |  | 1.38(1.03;1.84) | 1.19(0.91;1.56) | 0.01 |
| rs9268368 | 405 | 113 | 674 | 277 | 424 | 156 | 745 | 227 | C | 41 | 1.58(1.22;2.05) |  | 1.38(1.03;1.84) | 1.19(0.91;1.56) | 0.01 |
| rs9268384 | 405 | 113 | 674 | 277 | 424 | 156 | 745 | 227 | G | 41 | 1.58(1.22;2.05) |  | 1.38(1.03;1.84) | 1.19(0.91;1.56) | 0.01 |
| rs9268394 | 403 | 113 | 672 | 276 | 422 | 156 | 741 | 227 | C | 41 | 1.57(1.21;2.04) |  | 1.38(1.03;1.84) | 1.19(0.91;1.56) | 0.01 |
| rs9268521 | 395 | 118 | 774 | 265 | 376 | 158 | 703 | 232 | C | 19 | 1.2(0.93;1.55) |  | 1.46(1.09;1.94) | 1.09(0.84;1.42) | 0.03 |
| rs9268528 | 189 | 49 | 980 | 334 | 147 | 70 | 932 | 320 | A | 39 | 1.35(0.95;1.91) |  | 1.87(1.21;2.9) | 1.32(0.93;1.87) | 0.04 |
| rs9268542 | 197 | 50 | 972 | 333 | 152 | 72 | 927 | 318 | A | 40 | 1.37(0.97;1.94) |  | 1.89(1.22;2.9) | 1.34(0.94;1.89) | 0.04 |
| rs9268556 | 197 | 50 | 972 | 333 | 152 | 72 | 927 | 318 | T | 40 | 1.37(0.97;1.94) |  | 1.89(1.22;2.9) | 1.34(0.94;1.89) | 0.04 |
| rs9268589 | 215 | 54 | 954 | 329 | 169 | 74 | 910 | 316 | G | 41 | 1.41(1.01;1.98) |  | 1.8(1.19;2.73) | 1.38(0.98;1.93) | 0.04 |
| rs9268606 | 215 | 54 | 954 | 329 | 169 | 74 | 910 | 316 | G | 41 | 1.41(1.01;1.98) |  | 1.8(1.19;2.73) | 1.38(0.98;1.93) | 0.04 |
| rs9268614 | 373 | 108 | 796 | 275 | 362 | 149 | 717 | 241 | G | 18 | 1.26(0.97;1.64) |  | 1.48(1.1;1.99) | 1.16(0.89;1.52) | 0.03 |
| rs9268615 | 224 | 54 | 945 | 329 | 172 | 74 | 907 | 316 | G | 41 | 1.48(1.06;2.07) |  | 1.83(1.21;2.77) | 1.44(1.03;2.01) | 0.04 |
| rs9268644 | 435 | 124 | 734 | 259 | 347 | 143 | 732 | 247 | A | 42 | 1.17(0.91;1.51) |  | 1.42(1.07;1.9) | 1.09(0.85;1.41) | 0.04 |
| rs9268645 | 215 | 54 | 954 | 329 | 169 | 73 | 910 | 317 | C | 41 | 1.42(1.01;1.98) |  | 1.78(1.17;2.71) | 1.38(0.99;1.93) | 0.04 |
| rs9268844 | 336 | 102 | 825 | 274 | 331 | 144 | 736 | 242 | G | 16 | 1.15(0.88;1.5) |  | 1.49(1.1;2.02) | 1.08(0.82;1.42) | 0.04 |
| rs9269821 | 608 | 187 | 561 | 196 | 537 | 215 | 539 | 174 | G | 28 | 1.19(0.94;1.52) |  | 1.31(1.03;1.66) | 1.05(0.82;1.34) | 0.04 |
| rs9271100 | 608 | 187 | 561 | 196 | 537 | 215 | 542 | 175 | T | 28 | 1.19(0.94;1.52) |  | 1.31(1.03;1.66) | 1.04(0.82;1.34) | 0.04 |
| rs9271209 | 608 | 187 | 561 | 196 | 537 | 215 | 542 | 175 | G | 28 | 1.19(0.94;1.52) |  | 1.31(1.03;1.66) | 1.04(0.82;1.34) | 0.04 |
| rs9271252 | 607 | 186 | 560 | 195 | 536 | 215 | 540 | 175 | C | 28 | 1.19(0.94;1.51) |  | 1.32(1.04;1.67) | 1.05(0.82;1.35) | 0.04 |
| rs9271255 | 607 | 187 | 560 | 196 | 537 | 215 | 542 | 175 | C | 28 | 1.2(0.94;1.52) |  | 1.31(1.03;1.66) | 1.04(0.82;1.34) | 0.03 |
| rs9272358 | 556 | 168 | 576 | 204 | 518 | 203 | 522 | 170 | A | 29 | 1.22(0.95;1.55) |  | 1.3(1.02;1.66) | 1.07(0.83;1.37) | 0.04 |
| rs9275141 | 298 | 83 | 871 | 300 | 253 | 110 | 826 | 280 | T | 49 | 1.22(0.92;1.62) |  | 1.56(1.11;2.2) | 1.16(0.87;1.55) | 0.04 |
| rs9275208 | 255 | 65 | 903 | 316 | 211 | 92 | 863 | 294 | G | 44 | 1.34(0.98;1.82) |  | 1.76(1.21;2.57) | 1.26(0.92;1.72) | 0.02 |
| rs9275563 | 248 | 63 | 917 | 320 | 204 | 89 | 873 | 301 | C | 44 | 1.34(0.98;1.83) |  | 1.76(1.2;2.58) | 1.28(0.93;1.75) | 0.03 |
| rs9275614 | 240 | 63 | 929 | 320 | 237 | 105 | 842 | 285 | G | 11 | 1.34(0.98;1.83) |  | 1.69(1.17;2.45) | 1.28(0.93;1.76) | 0.03 |
| rs9344790 | 718 | 216 | 451 | 166 | 670 | 259 | 408 | 131 | G | 38 | 1.23(0.97;1.57) |  | 1.27(1.02;1.57) | 1.04(0.8;1.34) | 0.04 |
| rs9348880 | 389 | 109 | 690 | 281 | 406 | 150 | 763 | 233 | T | 42 | 1.55(1.19;2.02) |  | 1.36(1.02;1.83) | 1.19(0.91;1.56) | 0.01 |
| rs9348881 | 389 | 109 | 690 | 281 | 406 | 150 | 763 | 233 | G | 42 | 1.55(1.19;2.02) |  | 1.36(1.02;1.83) | 1.19(0.91;1.56) | 0.01 |
| rs9348882 | 404 | 113 | 675 | 277 | 423 | 156 | 746 | 227 | T | 41 | 1.57(1.21;2.04) |  | 1.38(1.03;1.84) | 1.19(0.91;1.55) | 0.01 |
| rs9354960 | 601 | 160 | 568 | 223 | 527 | 200 | 552 | 190 | A | 30 | 1.5(1.18;1.91) |  | 1.4(1.1;1.8) | 1.27(0.99;1.63) | 0.01 |
| rs9357140 | 404 | 113 | 675 | 277 | 423 | 156 | 746 | 227 | A | 41 | 1.57(1.21;2.04) |  | 1.38(1.03;1.84) | 1.19(0.91;1.55) | 0.01 |
| rs9358854 | 143 | 28 | 1026 | 355 | 112 | 52 | 966 | 338 | G | 32 | 1.87(1.21;2.91) |  | 2.49(1.45;4.28) | 1.84(1.18;2.86) | 0.03 |
| rs9366793 | 389 | 109 | 690 | 281 | 406 | 150 | 763 | 233 | C | 42 | 1.55(1.19;2.02) |  | 1.36(1.02;1.83) | 1.19(0.91;1.56) | 0.01 |
| rs9368713 | 404 | 113 | 675 | 277 | 423 | 156 | 746 | 227 | C | 41 | 1.57(1.21;2.04) |  | 1.38(1.03;1.84) | 1.19(0.91;1.55) | 0.01 |
| rs9370332 | 564 | 156 | 604 | 227 | 473 | 182 | 606 | 208 | G | 32 | 1.32(1.04;1.68) |  | 1.38(1.07;1.78) | 1.16(0.91;1.49) | 0.02 |
| rs9374263 | 385 | 112 | 784 | 271 | 352 | 149 | 727 | 241 | G | 18 | 1.2(0.93;1.56) |  | 1.44(1.07;1.93) | 1.12(0.86;1.46) | 0.04 |
| rs9374266 | 115 | 23 | 964 | 366 | 109 | 47 | 1059 | 336 | C | 31 | 1.83(1.14;2.94) |  | 2.17(1.22;3.88) | 1.57(0.98;2.52) | 0.04 |
| rs9380290 | 389 | 109 | 690 | 281 | 406 | 150 | 763 | 233 | G | 42 | 1.55(1.19;2.02) |  | 1.36(1.02;1.83) | 1.19(0.91;1.56) | 0.01 |
| rs9394368 | 174 | 38 | 994 | 345 | 153 | 70 | 926 | 320 | G | 37 | 1.61(1.1;2.36) |  | 1.99(1.25;3.17) | 1.58(1.08;2.32) | 0.04 |
| rs9398274 | 432 | 131 | 737 | 252 | 402 | 168 | 677 | 222 | T | 21 | 1.18(0.92;1.52) |  | 1.4(1.06;1.84) | 1.08(0.84;1.4) | 0.04 |
| rs9401003 | 473 | 140 | 696 | 243 | 404 | 167 | 675 | 223 | G | 22 | 1.16(0.9;1.48) |  | 1.39(1.06;1.82) | 1.06(0.82;1.36) | 0.04 |
| rs9405090 | 404 | 113 | 675 | 277 | 423 | 156 | 746 | 227 | G | 41 | 1.57(1.21;2.04) |  | 1.38(1.03;1.84) | 1.19(0.91;1.55) | 0.01 |
| rs9406138 | 776 | 235 | 391 | 148 | 714 | 274 | 363 | 115 | G | 19 | 1.28(1;1.64) |  | 1.26(1.02;1.55) | 1.01(0.77;1.32) | 0.02 |
| rs9452592 | 601 | 167 | 568 | 216 | 552 | 209 | 526 | 181 | A | 29 | 1.33(1.05;1.69) |  | 1.33(1.04;1.69) | 1.18(0.92;1.52) | 0.04 |
| rs9460636 | 220 | 64 | 859 | 326 | 221 | 92 | 948 | 291 | C | 10 | 1.35(0.98;1.85) |  | 1.49(1.01;2.18) | 1.11(0.8;1.52) | 0.03 |
| rs9481408 | 544 | 170 | 535 | 220 | 555 | 190 | 614 | 193 | T | 31 | 1.39(1.09;1.76) |  | 1.16(0.9;1.48) | 1.05(0.82;1.34) | 0.03 |
| rs9492461 | 267 | 73 | 902 | 310 | 245 | 110 | 834 | 280 | C | 12 | 1.36(1;1.83) |  | 1.67(1.16;2.38) | 1.29(0.95;1.75) | 0.03 |
| rs9498070 | 1072 | 322 | 97 | 61 | 961 | 350 | 118 | 40 | A | 5 | 1.99(1.39;2.84) |  | 1.19(0.99;1.43) | 1.04(0.7;1.54) | 0.01 |
| rs970218 | 113 | 23 | 966 | 367 | 104 | 46 | 1064 | 337 | G | 30 | 1.81(1.12;2.9) |  | 2.17(1.21;3.89) | 1.54(0.96;2.48) | 0.04 |
| rs973037 | 402 | 113 | 672 | 276 | 420 | 155 | 745 | 226 | T | 41 | 1.57(1.21;2.04) |  | 1.37(1.03;1.84) | 1.18(0.9;1.54) | 0.01 |
| rs990060 | 721 | 216 | 448 | 167 | 670 | 259 | 409 | 131 | C | 38 | 1.25(0.98;1.6) |  | 1.27(1.02;1.58) | 1.04(0.8;1.34) | 0.03 |
| chr 7 |  |  |  |  |  |  |  |  |  |  |  |  |  |  |  |
| rs10233492 | 172 | 42 | 997 | 341 | 120 | 61 | 959 | 329 | A | 35 | 1.38(0.95;2) |  | 2.12(1.32;3.41) | 1.34(0.92;1.95) | 0.03 |
| rs10234851 | 514 | 145 | 565 | 245 | 505 | 189 | 664 | 194 | G | 34 | 1.63(1.27;2.08) |  | 1.4(1.08;1.81) | 1.09(0.85;1.41) | 0.00 |
| rs10263966 | 172 | 42 | 994 | 340 | 120 | 62 | 958 | 328 | G | 36 | 1.38(0.95;2) |  | 2.17(1.35;3.47) | 1.34(0.92;1.95) | 0.03 |
| rs10275469 | 475 | 144 | 694 | 238 | 427 | 177 | 652 | 212 | G | 23 | 1.14(0.89;1.45) |  | 1.36(1.05;1.78) | 1.04(0.81;1.34) | 0.04 |
| rs10278273 | 314 | 96 | 765 | 294 | 297 | 122 | 872 | 261 | C | 15 | 1.3(0.99;1.71) |  | 1.44(1.04;1.99) | 1.01(0.77;1.34) | 0.01 |
| rs10278661 | 172 | 43 | 995 | 339 | 120 | 60 | 958 | 329 | T | 36 | 1.34(0.92;1.94) |  | 2.04(1.27;3.27) | 1.31(0.9;1.9) | 0.04 |
| rs1034757 | 188 | 49 | 891 | 340 | 156 | 60 | 1012 | 322 | A | 38 | 1.48(1.05;2.1) |  | 1.61(1.03;2.51) | 1.25(0.88;1.77) | 0.04 |
| rs1045463 | 679 | 186 | 490 | 197 | 592 | 235 | 487 | 155 | T | 25 | 1.43(1.12;1.81) |  | 1.45(1.15;1.82) | 1.07(0.83;1.38) | 0.00 |
| rs10499795 | 434 | 123 | 637 | 265 | 449 | 150 | 707 | 230 | A | 38 | 1.46(1.14;1.89) |  | 1.22(0.92;1.62) | 1.16(0.9;1.5) | 0.03 |
| rs11979904 | 375 | 106 | 704 | 284 | 326 | 114 | 843 | 269 | C | 45 | 1.45(1.11;1.89) |  | 1.28(0.93;1.75) | 1.16(0.89;1.52) | 0.03 |
| rs12532433 | 474 | 144 | 695 | 239 | 376 | 166 | 703 | 224 | C | 22 | 1.09(0.85;1.39) |  | 1.39(1.06;1.82) | 1(0.78;1.29) | 0.04 |
| rs12537271 | 586 | 159 | 583 | 224 | 513 | 198 | 566 | 192 | T | 31 | 1.37(1.08;1.75) |  | 1.42(1.11;1.82) | 1.16(0.9;1.49) | 0.01 |
| rs12540728 | 791 | 228 | 378 | 155 | 723 | 278 | 356 | 112 | T | 18 | 1.43(1.12;1.83) |  | 1.32(1.07;1.63) | 1.05(0.8;1.37) | 0.00 |
| rs12699131 | 375 | 90 | 794 | 293 | 294 | 109 | 785 | 281 | A | 47 | 1.52(1.15;2) |  | 1.56(1.12;2.17) | 1.43(1.08;1.88) | 0.03 |
| rs1294875 | 257 | 68 | 912 | 315 | 192 | 89 | 887 | 301 | A | 45 | 1.29(0.95;1.75) |  | 1.67(1.14;2.43) | 1.24(0.91;1.69) | 0.04 |
| rs1345252 | 551 | 175 | 618 | 208 | 469 | 202 | 610 | 188 | T | 27 | 1.15(0.9;1.46) |  | 1.37(1.07;1.75) | 1.01(0.79;1.29) | 0.02 |
| rs1524756 | 359 | 105 | 810 | 278 | 305 | 136 | 774 | 254 | C | 16 | 1.18(0.9;1.53) |  | 1.5(1.1;2.04) | 1.1(0.84;1.44) | 0.03 |
| rs1626408 | 142 | 38 | 1026 | 343 | 118 | 66 | 961 | 324 | A | 6 | 1.32(0.89;1.95) |  | 2.21(1.36;3.59) | 1.29(0.87;1.91) | 0.03 |
| rs17635734 | 267 | 68 | 902 | 315 | 229 | 95 | 850 | 295 | T | 47 | 1.36(1;1.85) |  | 1.65(1.13;2.39) | 1.31(0.96;1.79) | 0.04 |
| rs2072312 | 873 | 268 | 296 | 115 | 750 | 285 | 329 | 105 | C | 15 | 1.27(0.98;1.66) |  | 1.22(0.99;1.49) | 1.02(0.78;1.33) | 0.04 |
| rs217373 | 211 | 46 | 957 | 337 | 200 | 81 | 879 | 309 | C | 41 | 1.59(1.11;2.26) |  | 1.9(1.24;2.9) | 1.53(1.07;2.19) | 0.03 |
| rs2215169 | 655 | 199 | 514 | 184 | 590 | 236 | 489 | 154 | T | 34 | 1.18(0.93;1.5) |  | 1.29(1.03;1.62) | 1.01(0.79;1.3) | 0.03 |
| rs2233404 | 163 | 43 | 1006 | 340 | 119 | 60 | 960 | 330 | A | 35 | 1.29(0.89;1.88) |  | 2(1.25;3.22) | 1.27(0.88;1.85) | 0.04 |
| rs2529038 | 529 | 162 | 639 | 221 | 469 | 191 | 610 | 199 | T | 26 | 1.14(0.9;1.46) |  | 1.33(1.03;1.72) | 1.03(0.81;1.33) | 0.04 |
| rs2531840 | 434 | 136 | 645 | 254 | 425 | 161 | 744 | 222 | A | 39 | 1.38(1.07;1.77) |  | 1.31(1;1.73) | 1.03(0.8;1.32) | 0.01 |
| rs2711091 | 530 | 162 | 639 | 221 | 470 | 192 | 609 | 198 | G | 26 | 1.15(0.9;1.46) |  | 1.34(1.04;1.72) | 1.03(0.81;1.32) | 0.04 |
| rs2711130 | 535 | 163 | 634 | 220 | 471 | 192 | 608 | 198 | C | 26 | 1.16(0.91;1.47) |  | 1.34(1.04;1.73) | 1.04(0.81;1.33) | 0.04 |
| rs2718016 | 466 | 132 | 703 | 251 | 406 | 173 | 673 | 217 | A | 22 | 1.28(0.99;1.64) |  | 1.49(1.13;1.95) | 1.12(0.87;1.45) | 0.01 |
| rs2944839 | 376 | 92 | 793 | 291 | 298 | 110 | 781 | 280 | C | 46 | 1.49(1.14;1.96) |  | 1.53(1.1;2.13) | 1.41(1.07;1.85) | 0.04 |
| rs3750145 | 791 | 228 | 378 | 155 | 723 | 278 | 356 | 112 | C | 18 | 1.43(1.11;1.83) |  | 1.32(1.07;1.63) | 1.05(0.8;1.37) | 0.00 |
| rs3917478 | 939 | 285 | 230 | 98 | 855 | 316 | 224 | 74 | C | 11 | 1.38(1.04;1.84) |  | 1.19(0.98;1.45) | 1.04(0.77;1.41) | 0.04 |
| rs40922 | 326 | 97 | 753 | 293 | 311 | 126 | 857 | 257 | A | 47 | 1.26(0.96;1.65) |  | 1.35(0.98;1.85) | 1.01(0.77;1.32) | 0.02 |
| rs420437 | 199 | 50 | 880 | 340 | 180 | 70 | 989 | 313 | C | 41 | 1.48(1.05;2.09) |  | 1.55(1.01;2.38) | 1.25(0.88;1.76) | 0.04 |
| rs4720172 | 536 | 152 | 633 | 231 | 465 | 191 | 614 | 199 | T | 34 | 1.38(1.08;1.76) |  | 1.48(1.14;1.91) | 1.16(0.9;1.49) | 0.01 |
| rs4720174 | 555 | 165 | 614 | 218 | 494 | 206 | 585 | 184 | C | 32 | 1.27(1;1.62) |  | 1.42(1.11;1.82) | 1.07(0.83;1.37) | 0.01 |
| rs4722116 | 339 | 102 | 740 | 288 | 338 | 129 | 831 | 254 | G | 45 | 1.29(0.98;1.68) |  | 1.31(0.96;1.78) | 1.03(0.78;1.35) | 0.03 |
| rs509834 | 277 | 76 | 887 | 307 | 237 | 111 | 839 | 278 | C | 12 | 1.23(0.91;1.65) |  | 1.64(1.15;2.33) | 1.15(0.85;1.56) | 0.03 |
| rs6461513 | 566 | 155 | 603 | 228 | 491 | 187 | 588 | 203 | C | 32 | 1.36(1.07;1.74) |  | 1.38(1.07;1.78) | 1.2(0.94;1.54) | 0.02 |
| rs6466265 | 469 | 133 | 700 | 250 | 415 | 167 | 664 | 223 | G | 22 | 1.3(1.02;1.67) |  | 1.39(1.06;1.83) | 1.2(0.93;1.54) | 0.04 |
| rs6467735 | 886 | 267 | 276 | 114 | 830 | 304 | 246 | 84 | T | 13 | 1.39(1.07;1.82) |  | 1.21(0.99;1.47) | 1.08(0.8;1.45) | 0.04 |
| rs6954269 | 190 | 49 | 963 | 329 | 141 | 67 | 926 | 319 | G | 37 | 1.34(0.94;1.9) |  | 1.94(1.24;3.02) | 1.3(0.92;1.86) | 0.03 |
| rs6964558 | 325 | 98 | 844 | 285 | 287 | 131 | 792 | 259 | A | 15 | 1.15(0.88;1.51) |  | 1.49(1.09;2.05) | 1.09(0.83;1.43) | 0.04 |
| rs6978212 | 314 | 83 | 855 | 300 | 268 | 114 | 811 | 276 | T | 49 | 1.37(1.03;1.82) |  | 1.59(1.13;2.23) | 1.3(0.97;1.73) | 0.03 |
| rs712825 | 785 | 237 | 374 | 143 | 690 | 259 | 384 | 130 | C | 19 | 1.31(1.02;1.69) |  | 1.25(1.01;1.54) | 1.09(0.84;1.4) | 0.04 |
| rs7357118 | 207 | 61 | 872 | 329 | 182 | 82 | 987 | 301 | C | 9 | 1.34(0.97;1.85) |  | 1.6(1.07;2.39) | 1.1(0.79;1.52) | 0.02 |
| rs756912 | 373 | 90 | 796 | 293 | 293 | 108 | 786 | 282 | C | 47 | 1.51(1.14;1.98) |  | 1.54(1.11;2.15) | 1.42(1.08;1.88) | 0.04 |
| rs7779018 | 775 | 234 | 394 | 149 | 706 | 271 | 372 | 119 | G | 42 | 1.27(0.99;1.63) |  | 1.25(1.01;1.55) | 1.04(0.8;1.36) | 0.03 |
| rs7790494 | 351 | 108 | 728 | 282 | 328 | 127 | 841 | 256 | T | 17 | 1.3(1;1.7) |  | 1.31(0.96;1.79) | 1.04(0.8;1.36) | 0.03 |
| rs7797991 | 742 | 231 | 337 | 159 | 775 | 255 | 394 | 128 | T | 19 | 1.48(1.15;1.89) |  | 1.08(0.87;1.34) | 1.04(0.8;1.35) | 0.03 |
| rs7800747 | 305 | 94 | 774 | 296 | 294 | 123 | 875 | 260 | T | 15 | 1.32(1;1.74) |  | 1.4(1.01;1.94) | 1.05(0.79;1.38) | 0.02 |
| rs7810874 | 115 | 29 | 1054 | 354 | 88 | 56 | 991 | 334 | A | 5 | 1.26(0.81;1.94) |  | 2.44(1.42;4.21) | 1.23(0.8;1.91) | 0.03 |
| rs848494 | 677 | 185 | 492 | 198 | 587 | 235 | 492 | 155 | G | 25 | 1.43(1.13;1.82) |  | 1.47(1.16;1.85) | 1.06(0.82;1.37) | 0.00 |
| rs9642617 | 359 | 110 | 719 | 280 | 336 | 129 | 830 | 254 | A | 17 | 1.32(1.01;1.71) |  | 1.31(0.97;1.79) | 1.05(0.8;1.37) | 0.02 |
| rs972400 | 760 | 232 | 409 | 151 | 687 | 265 | 392 | 125 | A | 20 | 1.22(0.96;1.57) |  | 1.26(1.01;1.55) | 1.01(0.78;1.3) | 0.03 |
| chr8 |  |  |  |  |  |  |  |  |  |  |  |  |  |  |  |
| rs10086120 | 214 | 59 | 865 | 331 | 215 | 80 | 954 | 303 | T | 44 | 1.43(1.04;1.98) |  | 1.47(0.99;2.18) | 1.2(0.86;1.66) | 0.04 |
| rs10101215 | 141 | 34 | 1028 | 349 | 116 | 58 | 963 | 332 | C | 34 | 1.48(0.98;2.24) |  | 2.2(1.32;3.68) | 1.46(0.96;2.2) | 0.04 |
| rs1010999 | 620 | 183 | 459 | 207 | 638 | 208 | 531 | 175 | G | 26 | 1.52(1.2;1.94) |  | 1.12(0.89;1.42) | 1.15(0.9;1.47) | 0.03 |
| rs1030431 | 689 | 208 | 479 | 175 | 601 | 244 | 478 | 146 | A | 35 | 1.23(0.97;1.57) |  | 1.32(1.05;1.65) | 1.01(0.78;1.29) | 0.01 |
| rs11136344 | 202 | 56 | 877 | 334 | 205 | 86 | 964 | 297 | T | 43 | 1.45(1.04;2.03) |  | 1.7(1.13;2.54) | 1.18(0.84;1.65) | 0.02 |
| rs11782455 | 476 | 145 | 603 | 245 | 474 | 160 | 695 | 223 | A | 36 | 1.29(1.01;1.65) |  | 1.18(0.9;1.54) | 1.02(0.79;1.31) | 0.04 |
| rs12542335 | 219 | 63 | 860 | 327 | 182 | 83 | 987 | 300 | T | 10 | 1.31(0.95;1.8) |  | 1.57(1.05;2.33) | 1.08(0.79;1.49) | 0.03 |
| rs12679938 | 682 | 215 | 487 | 168 | 598 | 240 | 481 | 150 | G | 35 | 1.2(0.95;1.53) |  | 1.28(1.02;1.6) | 1.03(0.8;1.32) | 0.04 |
| rs1269693 | 749 | 232 | 420 | 151 | 674 | 260 | 404 | 129 | G | 40 | 1.22(0.95;1.56) |  | 1.25(1.01;1.54) | 1.03(0.79;1.33) | 0.04 |
| rs1316738 | 349 | 93 | 820 | 290 | 271 | 110 | 808 | 280 | A | 48 | 1.41(1.07;1.85) |  | 1.56(1.12;2.18) | 1.33(1;1.75) | 0.03 |
| rs13253599 | 310 | 92 | 769 | 298 | 323 | 127 | 846 | 256 | T | 15 | 1.35(1.03;1.79) |  | 1.45(1.05;2) | 1.05(0.79;1.4) | 0.01 |
| rs13264831 | 761 | 218 | 392 | 163 | 678 | 252 | 391 | 131 | G | 20 | 1.46(1.15;1.87) |  | 1.31(1.06;1.63) | 1.1(0.85;1.42) | 0.01 |
| rs13265334 | 524 | 139 | 645 | 244 | 493 | 183 | 586 | 207 | T | 34 | 1.37(1.07;1.76) |  | 1.38(1.06;1.8) | 1.24(0.96;1.6) | 0.03 |
| rs13269097 | 723 | 201 | 446 | 182 | 653 | 237 | 426 | 153 | G | 23 | 1.41(1.11;1.8) |  | 1.3(1.04;1.63) | 1.19(0.92;1.53) | 0.03 |
| rs13271046 | 252 | 65 | 917 | 318 | 193 | 87 | 886 | 303 | T | 44 | 1.34(0.98;1.83) |  | 1.8(1.22;2.65) | 1.28(0.93;1.75) | 0.02 |
| rs13277801 | 733 | 222 | 436 | 161 | 643 | 258 | 436 | 132 | C | 38 | 1.3(1.02;1.66) |  | 1.32(1.06;1.64) | 1.01(0.79;1.31) | 0.01 |
| rs13277934 | 662 | 209 | 417 | 181 | 697 | 235 | 472 | 148 | C | 23 | 1.48(1.16;1.88) |  | 1.11(0.88;1.38) | 1.07(0.83;1.38) | 0.02 |
| rs1376772 | 223 | 64 | 946 | 319 | 173 | 80 | 906 | 310 | A | 9 | 1.3(0.95;1.79) |  | 1.75(1.18;2.61) | 1.26(0.92;1.74) | 0.04 |
| rs1442408 | 616 | 177 | 552 | 206 | 511 | 203 | 568 | 187 | A | 29 | 1.31(1.03;1.66) |  | 1.38(1.08;1.76) | 1.11(0.87;1.41) | 0.01 |
| rs1499439 | 574 | 180 | 595 | 203 | 553 | 225 | 526 | 165 | A | 30 | 1.16(0.91;1.47) |  | 1.32(1.04;1.67) | 1.01(0.78;1.29) | 0.03 |
| rs1564424 | 530 | 157 | 639 | 226 | 480 | 192 | 599 | 198 | C | 33 | 1.21(0.95;1.55) |  | 1.34(1.04;1.73) | 1.1(0.85;1.41) | 0.04 |
| rs16870364 | 594 | 173 | 575 | 210 | 483 | 201 | 596 | 189 | C | 31 | 1.28(1.01;1.63) |  | 1.44(1.12;1.84) | 1.06(0.83;1.35) | 0.01 |
| rs16897057 | 360 | 92 | 809 | 291 | 304 | 123 | 775 | 267 | A | 46 | 1.41(1.07;1.85) |  | 1.56(1.13;2.15) | 1.32(1;1.74) | 0.03 |
| rs16897060 | 361 | 92 | 808 | 291 | 305 | 123 | 774 | 267 | C | 46 | 1.41(1.07;1.86) |  | 1.56(1.13;2.14) | 1.32(1;1.74) | 0.03 |
| rs16919692 | 492 | 137 | 677 | 246 | 404 | 161 | 675 | 229 | C | 38 | 1.22(0.95;1.56) |  | 1.39(1.06;1.82) | 1.12(0.87;1.44) | 0.04 |
| rs17273109 | 950 | 295 | 219 | 88 | 856 | 318 | 223 | 72 | C | 10 | 1.41(1.06;1.89) |  | 1.19(0.98;1.44) | 1.05(0.77;1.42) | 0.04 |
| rs17674240 | 1033 | 318 | 136 | 65 | 948 | 348 | 131 | 42 | T | 6 | 1.65(1.19;2.31) |  | 1.18(0.98;1.42) | 1.01(0.69;1.48) | 0.02 |
| rs1801280 | 392 | 92 | 776 | 290 | 325 | 118 | 754 | 270 | C | 45 | 1.58(1.2;2.08) |  | 1.52(1.1;2.1) | 1.48(1.12;1.96) | 0.04 |
| rs2252534 | 443 | 126 | 726 | 257 | 361 | 153 | 718 | 237 | C | 20 | 1.23(0.95;1.58) |  | 1.49(1.12;1.98) | 1.11(0.86;1.43) | 0.02 |
| rs2326077 | 741 | 223 | 428 | 160 | 646 | 257 | 433 | 133 | C | 38 | 1.35(1.06;1.72) |  | 1.32(1.07;1.65) | 1.05(0.81;1.35) | 0.01 |
| rs2410556 | 300 | 90 | 779 | 300 | 271 | 106 | 898 | 277 | C | 14 | 1.26(0.96;1.67) |  | 1.33(0.95;1.86) | 1.04(0.78;1.37) | 0.04 |
| rs2922467 | 696 | 201 | 473 | 182 | 615 | 233 | 464 | 157 | C | 24 | 1.32(1.03;1.67) |  | 1.28(1.02;1.61) | 1.13(0.89;1.45) | 0.04 |
| rs312112 | 770 | 231 | 397 | 152 | 672 | 260 | 407 | 130 | T | 20 | 1.27(0.99;1.63) |  | 1.26(1.02;1.56) | 1.03(0.8;1.33) | 0.02 |
| rs3812429 | 301 | 92 | 868 | 291 | 246 | 119 | 833 | 271 | C | 14 | 1.1(0.83;1.45) |  | 1.57(1.12;2.19) | 1.04(0.78;1.38) | 0.03 |
| rs3824260 | 831 | 256 | 337 | 127 | 741 | 285 | 338 | 105 | A | 45 | 1.37(1.06;1.77) |  | 1.25(1.02;1.53) | 1.05(0.8;1.38) | 0.02 |
| rs3886841 | 243 | 63 | 926 | 320 | 218 | 97 | 860 | 293 | A | 11 | 1.45(1.06;1.99) |  | 1.73(1.18;2.53) | 1.39(1.01;1.91) | 0.03 |
| rs4282557 | 690 | 219 | 479 | 164 | 596 | 237 | 483 | 153 | T | 35 | 1.19(0.93;1.51) |  | 1.27(1.01;1.58) | 1.03(0.8;1.31) | 0.04 |
| rs441890 | 384 | 117 | 785 | 266 | 341 | 140 | 738 | 250 | C | 42 | 1.2(0.92;1.55) |  | 1.44(1.07;1.93) | 1.12(0.86;1.45) | 0.04 |
| rs4738684 | 734 | 220 | 435 | 163 | 646 | 257 | 433 | 133 | A | 38 | 1.36(1.07;1.73) |  | 1.33(1.07;1.65) | 1.05(0.82;1.36) | 0.01 |
| rs4739730 | 595 | 177 | 574 | 205 | 530 | 210 | 548 | 180 | A | 29 | 1.25(0.98;1.59) |  | 1.34(1.05;1.71) | 1.1(0.86;1.4) | 0.03 |
| rs4740144 | 647 | 189 | 522 | 194 | 585 | 230 | 494 | 160 | T | 26 | 1.29(1.02;1.64) |  | 1.34(1.06;1.69) | 1.09(0.84;1.39) | 0.02 |
| rs4976972 | 300 | 80 | 848 | 287 | 271 | 120 | 773 | 260 | C | 50 | 1.31(0.98;1.76) |  | 1.69(1.2;2.37) | 1.26(0.94;1.7) | 0.02 |
| rs6469206 | 365 | 98 | 714 | 292 | 347 | 123 | 821 | 260 | G | 45 | 1.42(1.09;1.86) |  | 1.27(0.93;1.74) | 1.16(0.88;1.52) | 0.04 |
| rs6470186 | 310 | 95 | 769 | 295 | 325 | 127 | 844 | 256 | C | 15 | 1.3(0.99;1.71) |  | 1.4(1.01;1.93) | 1.02(0.77;1.35) | 0.02 |
| rs6472653 | 318 | 82 | 851 | 301 | 269 | 113 | 810 | 277 | T | 49 | 1.42(1.07;1.9) |  | 1.65(1.17;2.31) | 1.33(1;1.78) | 0.02 |
| rs6982080 | 562 | 161 | 607 | 222 | 467 | 193 | 612 | 197 | T | 32 | 1.24(0.98;1.58) |  | 1.39(1.08;1.79) | 1.08(0.84;1.38) | 0.02 |
| rs6988602 | 310 | 94 | 769 | 296 | 329 | 127 | 840 | 256 | C | 15 | 1.33(1.01;1.75) |  | 1.4(1.01;1.92) | 1.05(0.79;1.38) | 0.02 |
| rs6996219 | 828 | 244 | 341 | 139 | 714 | 278 | 365 | 112 | G | 17 | 1.46(1.13;1.88) |  | 1.31(1.07;1.62) | 1.04(0.8;1.35) | 0.00 |
| rs6996358 | 855 | 263 | 314 | 120 | 790 | 298 | 288 | 91 | A | 15 | 1.3(1;1.69) |  | 1.22(1;1.49) | 1.01(0.76;1.34) | 0.03 |
| rs7005778 | 560 | 171 | 609 | 212 | 497 | 201 | 582 | 189 | A | 31 | 1.21(0.95;1.54) |  | 1.36(1.06;1.74) | 1.06(0.83;1.36) | 0.02 |
| rs7017060 | 132 | 30 | 947 | 360 | 123 | 52 | 1046 | 331 | G | 34 | 1.65(1.08;2.51) |  | 1.91(1.13;3.24) | 1.4(0.92;2.14) | 0.04 |
| rs7463863 | 304 | 84 | 847 | 293 | 274 | 121 | 787 | 263 | A | 50 | 1.3(0.98;1.73) |  | 1.62(1.16;2.26) | 1.21(0.9;1.61) | 0.02 |
| rs748960 | 217 | 63 | 862 | 327 | 216 | 96 | 953 | 287 | A | 10 | 1.44(1.04;1.97) |  | 1.63(1.11;2.4) | 1.15(0.84;1.59) | 0.01 |
| rs7815937 | 404 | 115 | 743 | 254 | 359 | 149 | 700 | 230 | G | 40 | 1.21(0.93;1.57) |  | 1.44(1.08;1.93) | 1.13(0.87;1.47) | 0.04 |
| rs7827910 | 688 | 218 | 478 | 164 | 596 | 236 | 483 | 152 | A | 35 | 1.19(0.93;1.52) |  | 1.26(1.01;1.58) | 1.02(0.8;1.3) | 0.04 |
| rs7832643 | 181 | 44 | 898 | 346 | 185 | 82 | 984 | 301 | G | 41 | 1.68(1.16;2.42) |  | 2.05(1.32;3.17) | 1.34(0.93;1.93) | 0.01 |
| rs7836428 | 909 | 282 | 260 | 101 | 822 | 305 | 257 | 85 | A | 12 | 1.35(1.03;1.78) |  | 1.21(0.99;1.47) | 1.01(0.76;1.35) | 0.03 |
| rs7837487 | 856 | 259 | 312 | 124 | 765 | 286 | 314 | 104 | A | 15 | 1.38(1.06;1.79) |  | 1.24(1.01;1.52) | 1.05(0.8;1.37) | 0.02 |
| rs7839808 | 1050 | 318 | 119 | 65 | 939 | 346 | 140 | 44 | G | 6 | 1.8(1.28;2.53) |  | 1.18(0.98;1.42) | 1.05(0.72;1.53) | 0.01 |
| rs7840935 | 397 | 105 | 770 | 278 | 354 | 142 | 725 | 248 | G | 43 | 1.31(1.01;1.71) |  | 1.48(1.09;2) | 1.22(0.93;1.59) | 0.03 |
| rs7846137 | 539 | 144 | 630 | 239 | 479 | 180 | 600 | 210 | C | 33 | 1.43(1.12;1.83) |  | 1.39(1.07;1.8) | 1.28(1;1.64) | 0.03 |
| rs7846607 | 351 | 104 | 818 | 279 | 271 | 124 | 808 | 266 | C | 15 | 1.23(0.94;1.61) |  | 1.58(1.15;2.18) | 1.14(0.87;1.49) | 0.02 |
| rs893043 | 82 | 20 | 1086 | 363 | 38 | 32 | 1040 | 358 | G | 23 | 1.37(0.81;2.32) |  | 3.85(1.9;7.8) | 1.37(0.81;2.32) | 0.04 |
| rs9298186 | 165 | 39 | 914 | 351 | 143 | 58 | 1026 | 325 | A | 37 | 1.54(1.05;2.26) |  | 1.73(1.07;2.79) | 1.3(0.89;1.91) | 0.04 |
| rs9649981 | 159 | 41 | 920 | 349 | 142 | 64 | 1026 | 319 | G | 37 | 1.5(1.03;2.17) |  | 1.8(1.13;2.86) | 1.25(0.86;1.81) | 0.03 |
| rs972393 | 289 | 65 | 880 | 318 | 222 | 89 | 857 | 301 | G | 47 | 1.7(1.25;2.32) |  | 1.76(1.21;2.57) | 1.61(1.18;2.2) | 0.03 |
| chr 9 |  |  |  |  |  |  |  |  |  |  |  |  |  |  |  |
| rs1021493 | 733 | 213 | 436 | 170 | 658 | 247 | 421 | 143 | A | 22 | 1.37(1.07;1.74) |  | 1.28(1.03;1.59) | 1.15(0.89;1.48) | 0.03 |
| rs1021494 | 724 | 210 | 445 | 173 | 648 | 245 | 431 | 145 | T | 22 | 1.37(1.07;1.74) |  | 1.28(1.03;1.6) | 1.15(0.89;1.48) | 0.03 |
| rs10306150 | 272 | 81 | 807 | 309 | 288 | 110 | 881 | 273 | C | 13 | 1.32(0.99;1.76) |  | 1.34(0.95;1.89) | 1.08(0.81;1.45) | 0.04 |
| rs1041205 | 918 | 272 | 251 | 111 | 835 | 307 | 244 | 83 | G | 12 | 1.37(1.05;1.8) |  | 1.21(0.99;1.47) | 1.05(0.78;1.41) | 0.04 |
| rs10758658 | 809 | 242 | 360 | 141 | 693 | 258 | 386 | 132 | A | 19 | 1.32(1.03;1.7) |  | 1.24(1.01;1.54) | 1.08(0.84;1.4) | 0.04 |
| rs10781317 | 491 | 135 | 677 | 248 | 468 | 183 | 611 | 207 | G | 35 | 1.35(1.06;1.74) |  | 1.44(1.1;1.89) | 1.2(0.93;1.55) | 0.02 |
| rs10867178 | 674 | 178 | 495 | 205 | 591 | 228 | 488 | 162 | C | 25 | 1.58(1.24;2) |  | 1.43(1.14;1.81) | 1.23(0.95;1.58) | 0.00 |
| rs10971025 | 406 | 121 | 763 | 262 | 370 | 158 | 709 | 232 | A | 19 | 1.13(0.87;1.46) |  | 1.41(1.06;1.87) | 1.05(0.81;1.36) | 0.04 |
| rs10973348 | 649 | 191 | 517 | 191 | 558 | 222 | 517 | 167 | A | 27 | 1.23(0.97;1.57) |  | 1.34(1.06;1.69) | 1.04(0.81;1.33) | 0.02 |
| rs10976810 | 413 | 119 | 756 | 264 | 372 | 153 | 707 | 237 | G | 19 | 1.25(0.97;1.61) |  | 1.42(1.06;1.89) | 1.16(0.89;1.5) | 0.04 |
| rs10990268 | 353 | 108 | 816 | 275 | 292 | 130 | 787 | 260 | C | 16 | 1.15(0.88;1.5) |  | 1.49(1.09;2.03) | 1.08(0.83;1.41) | 0.04 |
| rs11794708 | 591 | 167 | 578 | 216 | 532 | 189 | 547 | 201 | A | 30 | 1.4(1.1;1.78) |  | 1.33(1.04;1.7) | 1.25(0.98;1.6) | 0.04 |
| rs11794852 | 699 | 199 | 470 | 184 | 629 | 243 | 450 | 147 | A | 24 | 1.36(1.07;1.74) |  | 1.34(1.07;1.68) | 1.1(0.85;1.41) | 0.01 |
| rs12178 | 953 | 292 | 216 | 91 | 858 | 318 | 221 | 72 | G | 11 | 1.4(1.05;1.87) |  | 1.19(0.99;1.45) | 1.02(0.75;1.39) | 0.03 |
| rs12343867 | 599 | 172 | 570 | 211 | 545 | 196 | 534 | 194 | C | 29 | 1.39(1.09;1.77) |  | 1.32(1.04;1.69) | 1.23(0.97;1.58) | 0.04 |
| rs13286706 | 652 | 194 | 517 | 189 | 568 | 220 | 511 | 170 | G | 27 | 1.2(0.95;1.53) |  | 1.29(1.02;1.63) | 1.05(0.82;1.35) | 0.04 |
| rs13290752 | 560 | 174 | 519 | 216 | 575 | 202 | 594 | 181 | A | 29 | 1.41(1.11;1.8) |  | 1.17(0.91;1.49) | 1.05(0.82;1.34) | 0.02 |
| rs1331384 | 307 | 96 | 772 | 294 | 291 | 110 | 878 | 273 | A | 48 | 1.3(0.99;1.71) |  | 1.32(0.95;1.84) | 1.06(0.8;1.39) | 0.04 |
| rs1340510 | 601 | 176 | 568 | 207 | 564 | 217 | 515 | 173 | G | 28 | 1.27(1;1.61) |  | 1.34(1.05;1.7) | 1.1(0.86;1.41) | 0.03 |
| rs1468758 | 527 | 159 | 642 | 224 | 459 | 191 | 620 | 199 | T | 25 | 1.13(0.89;1.44) |  | 1.34(1.04;1.72) | 1.02(0.79;1.3) | 0.04 |
| rs1571566 | 689 | 195 | 480 | 188 | 594 | 224 | 485 | 166 | T | 25 | 1.43(1.12;1.82) |  | 1.34(1.06;1.68) | 1.18(0.92;1.51) | 0.02 |
| rs16892 | 764 | 220 | 405 | 163 | 712 | 267 | 367 | 123 | C | 19 | 1.44(1.13;1.84) |  | 1.28(1.03;1.58) | 1.18(0.9;1.53) | 0.03 |
| rs17175230 | 810 | 234 | 359 | 149 | 755 | 275 | 324 | 115 | G | 17 | 1.4(1.09;1.8) |  | 1.25(1.02;1.54) | 1.13(0.87;1.48) | 0.03 |
| rs1886372 | 372 | 107 | 797 | 276 | 331 | 142 | 748 | 248 | C | 44 | 1.18(0.91;1.54) |  | 1.46(1.08;1.97) | 1.1(0.84;1.44) | 0.04 |
| rs1888652 | 551 | 159 | 618 | 224 | 479 | 188 | 600 | 202 | T | 33 | 1.23(0.96;1.56) |  | 1.34(1.04;1.72) | 1.1(0.86;1.41) | 0.04 |
| rs2136693 | 355 | 98 | 814 | 285 | 303 | 128 | 776 | 262 | G | 16 | 1.24(0.94;1.62) |  | 1.54(1.12;2.12) | 1.15(0.88;1.52) | 0.03 |
| rs2150720 | 625 | 173 | 544 | 210 | 521 | 210 | 558 | 180 | A | 28 | 1.37(1.07;1.74) |  | 1.4(1.1;1.78) | 1.13(0.89;1.45) | 0.01 |
| rs2314 | 564 | 168 | 604 | 215 | 469 | 187 | 610 | 203 | T | 32 | 1.24(0.98;1.58) |  | 1.34(1.04;1.72) | 1.11(0.87;1.42) | 0.04 |
| rs2769614 | 1001 | 308 | 168 | 75 | 873 | 325 | 206 | 65 | T | 42 | 1.51(1.1;2.07) |  | 1.18(0.98;1.43) | 1.04(0.76;1.42) | 0.03 |
| rs2777019 | 123 | 24 | 956 | 366 | 101 | 46 | 1068 | 337 | C | 31 | 1.94(1.22;3.09) |  | 2.47(1.4;4.39) | 1.63(1.02;2.59) | 0.02 |
| rs2818940 | 792 | 236 | 377 | 147 | 738 | 278 | 341 | 112 | T | 18 | 1.36(1.06;1.75) |  | 1.24(1.01;1.53) | 1.12(0.86;1.47) | 0.04 |
| rs2818941 | 722 | 210 | 447 | 173 | 649 | 243 | 430 | 147 | A | 22 | 1.36(1.07;1.74) |  | 1.27(1.02;1.58) | 1.17(0.91;1.5) | 0.04 |
| rs348483 | 319 | 91 | 850 | 292 | 256 | 119 | 823 | 271 | C | 14 | 1.14(0.86;1.51) |  | 1.58(1.13;2.2) | 1.08(0.81;1.43) | 0.03 |
| rs3758180 | 567 | 170 | 602 | 213 | 445 | 179 | 634 | 211 | T | 26 | 1.2(0.95;1.53) |  | 1.34(1.04;1.73) | 1.09(0.86;1.39) | 0.04 |
| rs411167 | 672 | 198 | 497 | 185 | 589 | 232 | 490 | 158 | C | 34 | 1.24(0.98;1.58) |  | 1.32(1.05;1.65) | 1.04(0.81;1.34) | 0.02 |
| rs4313219 | 675 | 183 | 494 | 199 | 577 | 212 | 502 | 178 | A | 26 | 1.63(1.28;2.07) |  | 1.34(1.06;1.69) | 1.39(1.08;1.77) | 0.03 |
| rs4618769 | 495 | 142 | 673 | 241 | 466 | 182 | 613 | 208 | C | 35 | 1.25(0.98;1.6) |  | 1.37(1.05;1.79) | 1.14(0.88;1.46) | 0.04 |
| rs481294 | 698 | 209 | 470 | 173 | 623 | 239 | 456 | 151 | C | 24 | 1.25(0.99;1.6) |  | 1.29(1.03;1.61) | 1.07(0.83;1.37) | 0.03 |
| rs521239 | 685 | 203 | 483 | 180 | 610 | 237 | 469 | 153 | G | 24 | 1.3(1.02;1.65) |  | 1.32(1.06;1.66) | 1.07(0.83;1.38) | 0.02 |
| rs693520 | 520 | 146 | 648 | 237 | 457 | 187 | 622 | 203 | A | 34 | 1.26(0.98;1.6) |  | 1.4(1.08;1.81) | 1.11(0.87;1.43) | 0.02 |
| rs7029058 | 174 | 32 | 995 | 351 | 150 | 64 | 928 | 326 | C | 37 | 2.02(1.35;3.03) |  | 2.39(1.46;3.91) | 1.95(1.3;2.93) | 0.02 |
| rs7846890 | 579 | 164 | 590 | 219 | 503 | 190 | 576 | 200 | G | 31 | 1.31(1.03;1.67) |  | 1.33(1.04;1.71) | 1.18(0.92;1.51) | 0.04 |
| rs7855909 | 324 | 90 | 845 | 293 | 272 | 115 | 807 | 275 | C | 14 | 1.24(0.94;1.63) |  | 1.56(1.12;2.18) | 1.17(0.88;1.55) | 0.03 |
| rs838819 | 316 | 76 | 852 | 307 | 257 | 104 | 822 | 286 | C | 50 | 1.45(1.09;1.94) |  | 1.7(1.2;2.41) | 1.35(1.01;1.81) | 0.02 |
| rs866311 | 322 | 80 | 846 | 303 | 260 | 107 | 819 | 283 | T | 50 | 1.41(1.06;1.88) |  | 1.68(1.2;2.37) | 1.31(0.98;1.75) | 0.02 |
| rs868969 | 441 | 117 | 728 | 266 | 429 | 159 | 650 | 231 | T | 38 | 1.42(1.1;1.84) |  | 1.44(1.08;1.91) | 1.32(1.01;1.71) | 0.04 |
| rs912058 | 557 | 164 | 612 | 219 | 505 | 197 | 574 | 193 | T | 28 | 1.25(0.98;1.59) |  | 1.34(1.05;1.72) | 1.11(0.87;1.43) | 0.03 |
| rs913711 | 555 | 161 | 614 | 222 | 526 | 206 | 553 | 184 | A | 31 | 1.32(1.03;1.68) |  | 1.37(1.07;1.75) | 1.16(0.9;1.49) | 0.02 |
| rs9298754 | 611 | 186 | 467 | 204 | 620 | 209 | 549 | 174 | G | 28 | 1.5(1.18;1.91) |  | 1.17(0.92;1.48) | 1.07(0.84;1.37) | 0.01 |
| chr 10 |  |  |  |  |  |  |  |  |  |  |  |  |  |  |  |
| rs10508570 | 1000 | 303 | 169 | 80 | 907 | 334 | 172 | 56 | C | 8 | 1.55(1.14;2.11) |  | 1.18(0.98;1.42) | 1.09(0.78;1.52) | 0.04 |
| rs10733789 | 508 | 161 | 571 | 229 | 570 | 194 | 599 | 189 | C | 31 | 1.32(1.04;1.69) |  | 1.15(0.89;1.48) | 1.03(0.8;1.32) | 0.04 |
| rs10761741 | 390 | 107 | 689 | 283 | 415 | 156 | 753 | 227 | T | 41 | 1.6(1.23;2.08) |  | 1.48(1.1;1.98) | 1.17(0.89;1.54) | 0.00 |
| rs10761784 | 286 | 90 | 792 | 300 | 328 | 121 | 840 | 262 | T | 47 | 1.29(0.97;1.7) |  | 1.3(0.94;1.81) | 1.05(0.79;1.4) | 0.04 |
| rs10762563 | 216 | 51 | 863 | 339 | 220 | 91 | 949 | 292 | A | 42 | 1.74(1.24;2.45) |  | 1.9(1.27;2.86) | 1.37(0.97;1.94) | 0.01 |
| rs10786706 | 338 | 95 | 741 | 295 | 356 | 121 | 813 | 262 | T | 45 | 1.55(1.18;2.04) |  | 1.31(0.95;1.8) | 1.26(0.95;1.66) | 0.03 |
| rs10786794 | 328 | 97 | 751 | 293 | 334 | 122 | 835 | 261 | C | 46 | 1.36(1.03;1.78) |  | 1.3(0.95;1.79) | 1.1(0.83;1.44) | 0.03 |
| rs10822184 | 272 | 83 | 807 | 306 | 299 | 113 | 869 | 270 | C | 49 | 1.32(0.98;1.76) |  | 1.37(0.97;1.92) | 1.07(0.8;1.44) | 0.03 |
| rs10822186 | 274 | 83 | 805 | 307 | 300 | 113 | 869 | 270 | G | 49 | 1.34(1;1.78) |  | 1.38(0.98;1.94) | 1.08(0.81;1.45) | 0.03 |
| rs10824709 | 552 | 145 | 617 | 238 | 472 | 180 | 607 | 210 | T | 33 | 1.45(1.14;1.85) |  | 1.38(1.07;1.79) | 1.3(1.01;1.67) | 0.03 |
| rs10829573 | 263 | 69 | 816 | 321 | 252 | 89 | 917 | 294 | C | 48 | 1.48(1.09;2.01) |  | 1.4(0.96;2.02) | 1.23(0.91;1.67) | 0.04 |
| rs10829595 | 503 | 150 | 576 | 240 | 497 | 179 | 672 | 204 | T | 33 | 1.45(1.14;1.85) |  | 1.3(1;1.68) | 1.04(0.81;1.34) | 0.00 |
| rs10885273 | 113 | 21 | 1056 | 362 | 85 | 44 | 994 | 346 | A | 29 | 1.95(1.18;3.24) |  | 2.94(1.58;5.47) | 1.93(1.16;3.21) | 0.04 |
| rs10886849 | 844 | 271 | 235 | 119 | 915 | 299 | 254 | 84 | T | 12 | 1.6(1.22;2.09) |  | 1.05(0.86;1.27) | 1.05(0.78;1.41) | 0.03 |
| rs10901591 | 493 | 141 | 676 | 242 | 430 | 180 | 649 | 210 | C | 23 | 1.3(1.01;1.66) |  | 1.42(1.09;1.86) | 1.16(0.9;1.49) | 0.02 |
| rs11016687 | 666 | 199 | 503 | 184 | 562 | 233 | 516 | 157 | G | 33 | 1.24(0.98;1.58) |  | 1.36(1.08;1.71) | 1.01(0.78;1.29) | 0.01 |
| rs1108472 | 209 | 52 | 870 | 338 | 202 | 78 | 967 | 305 | C | 42 | 1.58(1.13;2.22) |  | 1.61(1.06;2.44) | 1.31(0.93;1.84) | 0.03 |
| rs11101342 | 518 | 156 | 651 | 227 | 463 | 191 | 616 | 199 | T | 25 | 1.11(0.87;1.41) |  | 1.34(1.04;1.72) | 1(0.78;1.29) | 0.04 |
| rs11195491 | 702 | 215 | 467 | 168 | 620 | 238 | 459 | 152 | G | 36 | 1.23(0.96;1.57) |  | 1.26(1.01;1.58) | 1.06(0.83;1.35) | 0.04 |
| rs11196361 | 808 | 241 | 360 | 142 | 710 | 268 | 369 | 122 | T | 18 | 1.37(1.06;1.76) |  | 1.26(1.02;1.56) | 1.09(0.84;1.41) | 0.02 |
| rs11197921 | 595 | 172 | 574 | 211 | 531 | 209 | 548 | 181 | C | 30 | 1.3(1.02;1.65) |  | 1.33(1.04;1.7) | 1.14(0.89;1.46) | 0.03 |
| rs11253471 | 225 | 53 | 944 | 330 | 161 | 71 | 918 | 319 | G | 40 | 1.46(1.04;2.06) |  | 1.95(1.28;2.99) | 1.41(1;1.99) | 0.03 |
| rs11254854 | 447 | 137 | 722 | 246 | 356 | 157 | 723 | 233 | T | 20 | 1.1(0.86;1.41) |  | 1.4(1.06;1.84) | 1.02(0.8;1.32) | 0.04 |
| rs11499158 | 633 | 203 | 446 | 186 | 652 | 218 | 517 | 165 | T | 25 | 1.39(1.09;1.77) |  | 1.12(0.89;1.41) | 1.02(0.8;1.31) | 0.03 |
| rs11592650 | 398 | 115 | 771 | 268 | 343 | 137 | 736 | 253 | G | 18 | 1.26(0.97;1.63) |  | 1.44(1.07;1.95) | 1.18(0.91;1.54) | 0.04 |
| rs11595114 | 544 | 164 | 624 | 219 | 458 | 184 | 620 | 205 | T | 25 | 1.14(0.89;1.45) |  | 1.34(1.04;1.73) | 1.03(0.81;1.31) | 0.04 |
| rs12218711 | 431 | 125 | 648 | 265 | 425 | 155 | 744 | 228 | C | 39 | 1.45(1.12;1.86) |  | 1.31(0.99;1.74) | 1.1(0.85;1.42) | 0.01 |
| rs12247028 | 441 | 121 | 727 | 262 | 380 | 165 | 699 | 225 | G | 41 | 1.24(0.96;1.6) |  | 1.52(1.15;2.02) | 1.1(0.84;1.42) | 0.01 |
| rs1250554 | 528 | 144 | 641 | 239 | 455 | 182 | 624 | 208 | T | 35 | 1.34(1.05;1.72) |  | 1.42(1.09;1.84) | 1.18(0.92;1.52) | 0.02 |
| rs12776774 | 165 | 49 | 914 | 341 | 145 | 76 | ### | 307 | A | 7 | 1.23(0.86;1.74) |  | 1.7(1.1;2.64) | 1.02(0.71;1.45) | 0.03 |
| rs12778125 | 497 | 152 | 670 | 229 | 420 | 177 | 653 | 212 | T | 24 | 1.16(0.91;1.49) |  | 1.38(1.06;1.79) | 1.07(0.83;1.37) | 0.04 |
| rs12779373 | 593 | 179 | 576 | 204 | 553 | 220 | 526 | 170 | T | 29 | 1.17(0.92;1.48) |  | 1.32(1.04;1.67) | 1.02(0.79;1.31) | 0.03 |
| rs1545214 | 639 | 183 | 528 | 199 | 530 | 212 | 549 | 178 | A | 28 | 1.3(1.02;1.65) |  | 1.38(1.09;1.75) | 1.08(0.85;1.38) | 0.01 |
| rs17784294 | 651 | 201 | 518 | 182 | 597 | 238 | 482 | 152 | A | 33 | 1.16(0.91;1.48) |  | 1.28(1.02;1.6) | 1.01(0.78;1.29) | 0.04 |
| rs1779384 | 467 | 138 | 701 | 245 | 390 | 165 | 689 | 225 | A | 21 | 1.18(0.92;1.51) |  | 1.42(1.08;1.86) | 1.07(0.83;1.38) | 0.03 |
| rs2123419 | 525 | 159 | 554 | 231 | 522 | 186 | 647 | 197 | T | 32 | 1.36(1.06;1.73) |  | 1.2(0.93;1.54) | 1.01(0.79;1.3) | 0.02 |
| rs2147268 | 737 | 214 | 421 | 160 | 638 | 246 | 425 | 138 | C | 21 | 1.32(1.03;1.69) |  | 1.33(1.07;1.66) | 1.07(0.83;1.38) | 0.01 |
| rs2148959 | 498 | 149 | 671 | 234 | 415 | 167 | 664 | 223 | T | 23 | 1.2(0.94;1.54) |  | 1.37(1.05;1.78) | 1.1(0.86;1.41) | 0.04 |
| rs2227551 | 666 | 181 | 503 | 202 | 562 | 215 | 517 | 175 | G | 27 | 1.48(1.16;1.88) |  | 1.38(1.09;1.74) | 1.22(0.95;1.55) | 0.01 |
| rs2227564 | 720 | 209 | 449 | 174 | 612 | 237 | 467 | 153 | T | 24 | 1.34(1.05;1.7) |  | 1.31(1.05;1.63) | 1.1(0.86;1.41) | 0.02 |
| rs2243897 | 495 | 147 | 674 | 236 | 416 | 167 | 663 | 223 | G | 23 | 1.22(0.95;1.56) |  | 1.38(1.05;1.8) | 1.11(0.87;1.43) | 0.04 |
| rs2275774 | 735 | 241 | 344 | 149 | 750 | 245 | 419 | 138 | G | 19 | 1.41(1.1;1.81) |  | 1.06(0.86;1.31) | 1.02(0.79;1.31) | 0.04 |
| rs2393368 | 279 | 66 | 889 | 317 | 226 | 95 | 853 | 295 | C | 47 | 1.54(1.13;2.1) |  | 1.75(1.2;2.54) | 1.46(1.07;2) | 0.03 |
| rs2393967 | 508 | 162 | 571 | 228 | 565 | 194 | 604 | 189 | C | 32 | 1.32(1.04;1.68) |  | 1.15(0.9;1.48) | 1.02(0.79;1.31) | 0.04 |
| rs2459449 | 687 | 192 | 482 | 191 | 574 | 224 | 505 | 166 | T | 26 | 1.41(1.11;1.79) |  | 1.36(1.08;1.72) | 1.14(0.89;1.46) | 0.01 |
| rs2463963 | 456 | 137 | 623 | 253 | 456 | 172 | 713 | 211 | A | 23 | 1.39(1.08;1.77) |  | 1.28(0.98;1.67) | 1.03(0.8;1.33) | 0.01 |
| rs2675662 | 413 | 111 | 755 | 272 | 343 | 150 | 736 | 240 | G | 43 | 1.35(1.04;1.75) |  | 1.59(1.19;2.14) | 1.19(0.92;1.55) | 0.01 |
| rs2688607 | 714 | 201 | 455 | 182 | 596 | 233 | 483 | 157 | A | 25 | 1.43(1.12;1.82) |  | 1.36(1.08;1.7) | 1.14(0.89;1.46) | 0.01 |
| rs2688608 | 396 | 111 | 773 | 272 | 326 | 144 | 753 | 246 | G | 44 | 1.28(0.99;1.66) |  | 1.55(1.15;2.08) | 1.16(0.89;1.51) | 0.02 |
| rs2688842 | 487 | 145 | 682 | 238 | 408 | 172 | 671 | 218 | A | 37 | 1.23(0.96;1.58) |  | 1.47(1.12;1.92) | 1.08(0.84;1.39) | 0.01 |
| rs2797496 | 647 | 202 | 427 | 186 | 644 | 213 | 509 | 169 | G | 25 | 1.47(1.16;1.87) |  | 1.14(0.9;1.43) | 1.07(0.84;1.37) | 0.02 |
| rs2803449 | 269 | 81 | 877 | 296 | 217 | 101 | 851 | 280 | G | 12 | 1.12(0.84;1.5) |  | 1.58(1.1;2.25) | 1.06(0.79;1.42) | 0.04 |
| rs3740170 | 149 | 45 | 930 | 345 | 156 | 73 | 1013 | 310 | A | 7 | 1.26(0.88;1.83) |  | 1.67(1.06;2.61) | 1.06(0.73;1.53) | 0.04 |
| rs4144552 | 574 | 171 | 505 | 219 | 571 | 195 | 598 | 188 | G | 30 | 1.45(1.14;1.85) |  | 1.22(0.95;1.55) | 1.04(0.81;1.32) | 0.01 |
| rs4620622 | 557 | 158 | 611 | 225 | 475 | 182 | 604 | 208 | G | 33 | 1.33(1.04;1.69) |  | 1.35(1.05;1.75) | 1.19(0.93;1.52) | 0.04 |
| rs4749364 | 232 | 66 | 847 | 324 | 225 | 88 | 944 | 295 | G | 44 | 1.33(0.98;1.82) |  | 1.46(1;2.13) | 1.11(0.81;1.51) | 0.04 |
| rs4750519 | 369 | 100 | 710 | 290 | 364 | 119 | 805 | 264 | T | 44 | 1.5(1.15;1.96) |  | 1.26(0.92;1.72) | 1.22(0.93;1.6) | 0.04 |
| rs619270 | 910 | 278 | 239 | 98 | 812 | 309 | 237 | 76 | A | 12 | 1.31(0.99;1.73) |  | 1.22(1;1.48) | 1(0.74;1.35) | 0.04 |
| rs6584627 | 573 | 171 | 506 | 219 | 573 | 195 | 596 | 188 | C | 30 | 1.45(1.14;1.84) |  | 1.21(0.95;1.55) | 1.04(0.81;1.33) | 0.01 |
| rs6602221 | 532 | 158 | 547 | 232 | 528 | 180 | 640 | 203 | G | 32 | 1.43(1.12;1.82) |  | 1.15(0.89;1.48) | 1.11(0.87;1.42) | 0.04 |
| rs7072888 | 381 | 89 | 788 | 294 | 329 | 124 | 750 | 266 | C | 45 | 1.6(1.22;2.11) |  | 1.58(1.15;2.17) | 1.49(1.13;1.97) | 0.03 |
| rs7078248 | 503 | 150 | 576 | 240 | 496 | 179 | 673 | 204 | A | 33 | 1.45(1.14;1.85) |  | 1.3(1;1.68) | 1.04(0.81;1.33) | 0.00 |
| rs7896518 | 385 | 104 | 689 | 283 | 409 | 155 | 747 | 224 | G | 41 | 1.6(1.22;2.08) |  | 1.5(1.11;2.02) | 1.17(0.89;1.54) | 0.00 |
| rs7912269 | 178 | 46 | 991 | 337 | 119 | 60 | 960 | 330 | C | 7 | 1.32(0.93;1.89) |  | 2.03(1.28;3.22) | 1.3(0.91;1.86) | 0.03 |
| rs7920142 | 537 | 156 | 632 | 227 | 437 | 183 | 642 | 207 | A | 25 | 1.17(0.92;1.49) |  | 1.37(1.06;1.78) | 1.05(0.82;1.34) | 0.03 |
| rs792724 | 404 | 115 | 765 | 268 | 367 | 148 | 712 | 242 | C | 42 | 1.28(0.99;1.66) |  | 1.49(1.11;1.99) | 1.17(0.9;1.52) | 0.02 |
| rs928571 | 500 | 148 | 578 | 242 | 497 | 179 | 672 | 204 | A | 33 | 1.47(1.15;1.87) |  | 1.3(1;1.69) | 1.05(0.81;1.35) | 0.00 |
| rs9664184 | 185 | 50 | 894 | 340 | 179 | 70 | 990 | 313 | C | 38 | 1.46(1.03;2.07) |  | 1.58(1.02;2.44) | 1.23(0.87;1.74) | 0.04 |
| rs999994 | 138 | 35 | 1031 | 348 | 97 | 54 | 982 | 336 | T | 6 | 1.31(0.87;1.96) |  | 2.24(1.34;3.75) | 1.29(0.86;1.93) | 0.04 |
| chr 11 |  |  |  |  |  |  |  |  |  |  |  |  |  |  |  |
| rs10466659 | 481 | 138 | 686 | 243 | 426 | 177 | 649 | 211 | G | 23 | 1.22(0.95;1.57) |  | 1.43(1.09;1.87) | 1.09(0.84;1.4) | 0.02 |
| rs10500844 | 519 | 156 | 560 | 234 | 566 | 184 | 602 | 199 | C | 31 | 1.43(1.12;1.82) |  | 1.15(0.89;1.48) | 1.11(0.87;1.43) | 0.04 |
| rs10502207 | 124 | 33 | 955 | 357 | 104 | 50 | 1065 | 333 | C | 33 | 1.42(0.94;2.15) |  | 1.95(1.15;3.31) | 1.21(0.8;1.83) | 0.04 |
| rs1051992 | 251 | 59 | 918 | 324 | 200 | 81 | 879 | 308 | G | 43 | 1.52(1.1;2.1) |  | 1.84(1.24;2.74) | 1.45(1.05;2) | 0.02 |
| rs1054997 | 260 | 80 | 819 | 310 | 237 | 102 | 932 | 281 | A | 12 | 1.25(0.94;1.67) |  | 1.43(1;2.03) | 1.02(0.76;1.37) | 0.03 |
| rs1075127 | 565 | 174 | 514 | 216 | 540 | 189 | 629 | 194 | C | 30 | 1.36(1.07;1.73) |  | 1.16(0.9;1.48) | 1.03(0.81;1.31) | 0.03 |
| rs10765186 | 273 | 85 | 805 | 303 | 249 | 96 | 918 | 287 | T | 49 | 1.29(0.97;1.72) |  | 1.4(0.98;1.99) | 1.06(0.8;1.42) | 0.03 |
| rs10765987 | 423 | 115 | 746 | 268 | 356 | 150 | 723 | 240 | T | 42 | 1.3(1.01;1.69) |  | 1.49(1.11;2) | 1.19(0.92;1.55) | 0.02 |
| rs10767634 | 387 | 84 | 782 | 299 | 349 | 137 | 730 | 253 | C | 43 | 1.68(1.27;2.22) |  | 1.7(1.24;2.33) | 1.51(1.14;2.01) | 0.01 |
| rs10789819 | 568 | 168 | 600 | 215 | 473 | 192 | 606 | 197 | A | 32 | 1.14(0.9;1.45) |  | 1.33(1.04;1.71) | 1.02(0.8;1.3) | 0.04 |
| rs10789820 | 568 | 168 | 598 | 214 | 473 | 192 | 605 | 197 | A | 32 | 1.14(0.89;1.45) |  | 1.33(1.04;1.71) | 1.03(0.8;1.31) | 0.04 |
| rs10831106 | 384 | 93 | 781 | 288 | 338 | 133 | 736 | 257 | G | 44 | 1.42(1.08;1.87) |  | 1.54(1.13;2.11) | 1.34(1.01;1.77) | 0.03 |
| rs10834401 | 905 | 270 | 264 | 113 | 830 | 310 | 249 | 80 | A | 12 | 1.43(1.09;1.87) |  | 1.23(1.01;1.49) | 1.06(0.79;1.43) | 0.02 |
| rs10891872 | 124 | 33 | 955 | 357 | 104 | 50 | 1065 | 333 | A | 33 | 1.42(0.94;2.15) |  | 1.95(1.15;3.31) | 1.21(0.8;1.83) | 0.04 |
| rs11026755 | 856 | 253 | 313 | 130 | 726 | 278 | 353 | 112 | A | 16 | 1.38(1.07;1.79) |  | 1.28(1.04;1.57) | 1.01(0.77;1.31) | 0.01 |
| rs11213245 | 459 | 119 | 710 | 264 | 423 | 172 | 656 | 218 | A | 38 | 1.42(1.1;1.84) |  | 1.49(1.13;1.97) | 1.27(0.97;1.65) | 0.02 |
| rs11214677 | 243 | 53 | 925 | 330 | 198 | 81 | 881 | 309 | T | 43 | 1.55(1.11;2.17) |  | 1.86(1.24;2.79) | 1.49(1.07;2.08) | 0.03 |
| rs11217593 | 349 | 109 | 729 | 281 | 350 | 137 | 819 | 246 | A | 17 | 1.35(1.04;1.76) |  | 1.34(0.99;1.82) | 1.05(0.81;1.38) | 0.02 |
| rs11229389 | 459 | 140 | 620 | 250 | 480 | 176 | 689 | 207 | A | 36 | 1.4(1.09;1.79) |  | 1.24(0.95;1.61) | 1.06(0.82;1.37) | 0.02 |
| rs11237269 | 216 | 56 | 953 | 327 | 197 | 86 | 882 | 304 | A | 44 | 1.39(0.99;1.94) |  | 1.75(1.17;2.63) | 1.35(0.96;1.89) | 0.04 |
| rs11237805 | 819 | 249 | 350 | 134 | 712 | 272 | 367 | 118 | G | 17 | 1.25(0.97;1.61) |  | 1.23(1;1.52) | 1.02(0.78;1.32) | 0.04 |
| rs1144397 | 343 | 102 | 736 | 288 | 334 | 123 | 835 | 260 | T | 45 | 1.26(0.97;1.65) |  | 1.27(0.93;1.74) | 1.03(0.78;1.35) | 0.04 |
| rs11601356 | 726 | 201 | 443 | 182 | 628 | 230 | 450 | 160 | A | 23 | 1.45(1.14;1.85) |  | 1.28(1.03;1.61) | 1.24(0.97;1.59) | 0.04 |
| rs11602707 | 526 | 152 | 643 | 231 | 457 | 185 | 622 | 205 | T | 25 | 1.21(0.95;1.54) |  | 1.35(1.04;1.75) | 1.1(0.86;1.41) | 0.04 |
| rs12224346 | 211 | 51 | 958 | 332 | 153 | 67 | 926 | 323 | A | 39 | 1.35(0.96;1.9) |  | 1.84(1.2;2.83) | 1.32(0.94;1.86) | 0.04 |
| rs12285111 | 483 | 140 | 686 | 243 | 430 | 179 | 649 | 211 | G | 23 | 1.21(0.94;1.55) |  | 1.42(1.09;1.86) | 1.08(0.83;1.39) | 0.02 |
| rs12576775 | 819 | 249 | 350 | 134 | 710 | 272 | 369 | 118 | G | 17 | 1.25(0.97;1.61) |  | 1.24(1.01;1.52) | 1.01(0.78;1.31) | 0.03 |
| rs12786091 | 532 | 158 | 547 | 232 | 572 | 196 | 596 | 187 | C | 30 | 1.54(1.2;1.96) |  | 1.24(0.96;1.59) | 1.12(0.87;1.44) | 0.01 |
| rs12807171 | 499 | 135 | 670 | 248 | 475 | 185 | 604 | 205 | G | 35 | 1.35(1.05;1.73) |  | 1.42(1.09;1.84) | 1.2(0.93;1.55) | 0.02 |
| rs1318781 | 571 | 158 | 598 | 225 | 508 | 190 | 571 | 200 | A | 32 | 1.36(1.07;1.74) |  | 1.36(1.06;1.75) | 1.21(0.94;1.55) | 0.03 |
| rs1350762 | 227 | 74 | 852 | 316 | 195 | 85 | 974 | 298 | G | 44 | 1.22(0.9;1.66) |  | 1.45(0.99;2.13) | 1.01(0.75;1.38) | 0.04 |
| rs1615640 | 575 | 163 | 594 | 220 | 494 | 192 | 585 | 198 | C | 32 | 1.33(1.04;1.69) |  | 1.36(1.06;1.75) | 1.17(0.91;1.5) | 0.03 |
| rs17138230 | 817 | 250 | 352 | 133 | 711 | 272 | 368 | 118 | T | 17 | 1.23(0.95;1.58) |  | 1.23(1;1.51) | 1.01(0.78;1.31) | 0.04 |
| rs174570 | 350 | 103 | 819 | 280 | 280 | 129 | 799 | 261 | T | 15 | 1.21(0.93;1.58) |  | 1.54(1.13;2.12) | 1.13(0.86;1.48) | 0.03 |
| rs1783925 | 573 | 162 | 596 | 221 | 491 | 191 | 588 | 199 | G | 32 | 1.33(1.04;1.69) |  | 1.37(1.06;1.76) | 1.17(0.92;1.5) | 0.03 |
| rs1792282 | 624 | 185 | 545 | 198 | 531 | 208 | 548 | 182 | A | 29 | 1.22(0.96;1.55) |  | 1.31(1.03;1.66) | 1.08(0.84;1.38) | 0.04 |
| rs1793062 | 335 | 92 | 834 | 291 | 282 | 119 | 797 | 271 | C | 47 | 1.28(0.97;1.68) |  | 1.56(1.12;2.16) | 1.2(0.91;1.59) | 0.03 |
| rs1850875 | 340 | 86 | 828 | 296 | 309 | 126 | 770 | 264 | T | 47 | 1.36(1.03;1.8) |  | 1.54(1.11;2.13) | 1.29(0.97;1.71) | 0.04 |
| rs1870675 | 227 | 74 | 852 | 316 | 195 | 85 | 974 | 298 | G | 45 | 1.22(0.9;1.66) |  | 1.45(0.99;2.13) | 1.01(0.75;1.38) | 0.04 |
| rs1938623 | 609 | 166 | 559 | 217 | 533 | 208 | 546 | 182 | C | 30 | 1.36(1.07;1.73) |  | 1.4(1.1;1.79) | 1.14(0.89;1.46) | 0.01 |
| rs1940742 | 565 | 174 | 513 | 216 | 542 | 189 | 627 | 194 | G | 30 | 1.36(1.07;1.73) |  | 1.15(0.9;1.47) | 1.03(0.81;1.31) | 0.03 |
| rs1944055 | 176 | 46 | 993 | 337 | 156 | 72 | 923 | 318 | G | 8 | 1.39(0.96;2) |  | 1.88(1.2;2.94) | 1.36(0.94;1.96) | 0.04 |
| rs2072114 | 802 | 247 | 277 | 143 | 810 | 266 | 359 | 117 | G | 16 | 1.55(1.2;2) |  | 1.09(0.89;1.34) | 1(0.77;1.3) | 0.01 |
| rs2077654 | 258 | 82 | 911 | 301 | 232 | 113 | 847 | 277 | G | 12 | 1.04(0.78;1.4) |  | 1.56(1.1;2.22) | 1(0.75;1.34) | 0.04 |
| rs2446884 | 227 | 74 | 852 | 316 | 194 | 85 | 975 | 298 | G | 44 | 1.22(0.9;1.66) |  | 1.46(0.99;2.13) | 1.01(0.75;1.38) | 0.04 |
| rs2446886 | 798 | 239 | 371 | 144 | 709 | 266 | 370 | 124 | C | 42 | 1.3(1.02;1.68) |  | 1.24(1;1.52) | 1.08(0.83;1.4) | 0.04 |
| rs2446898 | 244 | 79 | 835 | 311 | 215 | 92 | 954 | 291 | A | 46 | 1.23(0.91;1.65) |  | 1.42(0.98;2.04) | 1.01(0.75;1.37) | 0.04 |
| rs2446901 | 642 | 185 | 527 | 198 | 566 | 219 | 513 | 171 | T | 31 | 1.35(1.06;1.71) |  | 1.34(1.06;1.7) | 1.14(0.89;1.46) | 0.02 |
| rs2512868 | 848 | 253 | 321 | 130 | 766 | 292 | 313 | 98 | T | 15 | 1.36(1.05;1.75) |  | 1.25(1.02;1.53) | 1.02(0.77;1.35) | 0.01 |
| rs2513634 | 168 | 44 | 911 | 346 | 141 | 60 | 1028 | 323 | C | 38 | 1.48(1.03;2.13) |  | 1.74(1.09;2.77) | 1.24(0.86;1.79) | 0.03 |
| rs2515294 | 227 | 74 | 852 | 316 | 195 | 85 | 974 | 298 | C | 44 | 1.22(0.9;1.66) |  | 1.45(0.99;2.13) | 1.01(0.75;1.38) | 0.04 |
| rs2716450 | 700 | 184 | 469 | 199 | 622 | 228 | 457 | 161 | T | 25 | 1.56(1.23;1.99) |  | 1.36(1.08;1.72) | 1.28(0.99;1.64) | 0.01 |
| rs2845839 | 918 | 274 | 251 | 109 | 812 | 303 | 267 | 87 | A | 13 | 1.41(1.07;1.85) |  | 1.22(1;1.49) | 1.04(0.78;1.39) | 0.02 |
| rs286902 | 448 | 119 | 721 | 264 | 419 | 165 | 660 | 225 | C | 38 | 1.39(1.08;1.8) |  | 1.51(1.14;2) | 1.24(0.95;1.61) | 0.01 |
| rs308780 | 867 | 265 | 302 | 118 | 736 | 282 | 343 | 108 | T | 47 | 1.25(0.96;1.63) |  | 1.22(1;1.49) | 1(0.77;1.31) | 0.04 |
| rs3133435 | 227 | 74 | 852 | 316 | 195 | 85 | 974 | 298 | G | 44 | 1.22(0.9;1.66) |  | 1.45(0.99;2.13) | 1.01(0.75;1.38) | 0.04 |
| rs353565 | 499 | 144 | 670 | 239 | 425 | 184 | 654 | 206 | C | 23 | 1.23(0.96;1.57) |  | 1.45(1.12;1.89) | 1.07(0.83;1.37) | 0.01 |
| rs3781884 | 323 | 89 | 846 | 294 | 264 | 112 | 815 | 278 | G | 14 | 1.33(1;1.76) |  | 1.55(1.11;2.17) | 1.26(0.95;1.68) | 0.04 |
| rs3892259 | 396 | 114 | 683 | 276 | 364 | 125 | 804 | 258 | G | 43 | 1.49(1.15;1.93) |  | 1.25(0.93;1.69) | 1.19(0.92;1.55) | 0.03 |
| rs3913857 | 374 | 90 | 795 | 293 | 327 | 125 | 752 | 265 | A | 45 | 1.42(1.08;1.88) |  | 1.51(1.09;2.08) | 1.35(1.02;1.79) | 0.04 |
| rs4148636 | 843 | 276 | 235 | 114 | 905 | 300 | 261 | 83 | T | 12 | 1.55(1.18;2.04) |  | 1.04(0.86;1.27) | 1(0.75;1.34) | 0.02 |
| rs4237696 | 897 | 268 | 271 | 115 | 807 | 306 | 272 | 84 | A | 49 | 1.34(1.03;1.76) |  | 1.22(1;1.49) | 1.02(0.76;1.36) | 0.03 |
| rs4245170 | 569 | 168 | 600 | 215 | 474 | 192 | 605 | 198 | A | 32 | 1.14(0.9;1.45) |  | 1.33(1.04;1.71) | 1.03(0.8;1.31) | 0.04 |
| rs4309189 | 569 | 168 | 600 | 215 | 475 | 192 | 604 | 198 | T | 32 | 1.14(0.9;1.45) |  | 1.33(1.03;1.7) | 1.03(0.81;1.31) | 0.04 |
| rs4620705 | 936 | 276 | 233 | 107 | 863 | 318 | 216 | 72 | G | 11 | 1.53(1.16;2.02) |  | 1.22(1.01;1.49) | 1.1(0.81;1.49) | 0.02 |
| rs4757993 | 440 | 114 | 729 | 269 | 345 | 139 | 734 | 251 | G | 41 | 1.38(1.07;1.79) |  | 1.52(1.13;2.04) | 1.25(0.97;1.63) | 0.02 |
| rs4936382 | 565 | 168 | 600 | 215 | 470 | 193 | 604 | 196 | G | 32 | 1.13(0.89;1.44) |  | 1.34(1.05;1.72) | 1.01(0.79;1.29) | 0.03 |
| rs4937277 | 565 | 174 | 514 | 216 | 541 | 189 | 628 | 194 | C | 30 | 1.36(1.07;1.73) |  | 1.15(0.9;1.48) | 1.03(0.81;1.31) | 0.03 |
| rs4937514 | 304 | 83 | 865 | 300 | 240 | 105 | 839 | 285 | G | 49 | 1.3(0.98;1.74) |  | 1.66(1.17;2.36) | 1.23(0.92;1.64) | 0.02 |
| rs538737 | 537 | 154 | 631 | 229 | 472 | 192 | 607 | 198 | G | 34 | 1.25(0.98;1.6) |  | 1.38(1.07;1.78) | 1.1(0.86;1.41) | 0.02 |
| rs540436 | 268 | 80 | 811 | 310 | 242 | 104 | 927 | 279 | T | 13 | 1.31(0.98;1.75) |  | 1.47(1.04;2.1) | 1.05(0.78;1.41) | 0.02 |
| rs540723 | 269 | 80 | 810 | 310 | 242 | 105 | 927 | 278 | A | 13 | 1.31(0.98;1.75) |  | 1.49(1.05;2.12) | 1.05(0.78;1.41) | 0.02 |
| rs548098 | 663 | 184 | 506 | 199 | 606 | 227 | 473 | 163 | A | 26 | 1.33(1.04;1.69) |  | 1.3(1.03;1.64) | 1.15(0.89;1.48) | 0.04 |
| rs556884 | 269 | 79 | 810 | 311 | 239 | 102 | 930 | 281 | G | 13 | 1.34(1;1.79) |  | 1.5(1.05;2.13) | 1.07(0.8;1.44) | 0.02 |
| rs582285 | 559 | 154 | 610 | 229 | 477 | 175 | 602 | 215 | G | 33 | 1.47(1.16;1.88) |  | 1.36(1.05;1.77) | 1.32(1.03;1.69) | 0.04 |
| rs610037 | 295 | 78 | 784 | 312 | 252 | 90 | 917 | 293 | A | 48 | 1.52(1.13;2.03) |  | 1.38(0.96;1.97) | 1.25(0.93;1.67) | 0.04 |
| rs611267 | 251 | 56 | 917 | 327 | 191 | 84 | 888 | 306 | G | 42 | 1.62(1.17;2.24) |  | 2.02(1.35;3.01) | 1.51(1.09;2.1) | 0.01 |
| rs657607 | 310 | 85 | 859 | 298 | 271 | 114 | 808 | 276 | T | 14 | 1.35(1.02;1.8) |  | 1.61(1.15;2.25) | 1.27(0.95;1.7) | 0.03 |
| rs6590292 | 540 | 149 | 628 | 234 | 485 | 194 | 594 | 196 | A | 33 | 1.34(1.05;1.71) |  | 1.43(1.11;1.84) | 1.15(0.9;1.49) | 0.01 |
| rs666160 | 910 | 269 | 259 | 114 | 800 | 303 | 279 | 87 | G | 14 | 1.44(1.1;1.89) |  | 1.25(1.03;1.52) | 1(0.75;1.34) | 0.01 |
| rs671188 | 837 | 250 | 332 | 133 | 740 | 279 | 339 | 111 | G | 44 | 1.43(1.11;1.84) |  | 1.24(1.01;1.53) | 1.13(0.86;1.47) | 0.03 |
| rs671789 | 568 | 161 | 601 | 222 | 496 | 191 | 583 | 199 | A | 32 | 1.32(1.04;1.68) |  | 1.35(1.05;1.74) | 1.18(0.92;1.51) | 0.03 |
| rs7116989 | 596 | 177 | 573 | 206 | 538 | 215 | 541 | 175 | G | 29 | 1.26(0.99;1.6) |  | 1.37(1.08;1.74) | 1.07(0.83;1.37) | 0.01 |
| rs7118743 | 471 | 131 | 698 | 252 | 367 | 154 | 712 | 236 | C | 21 | 1.32(1.03;1.7) |  | 1.43(1.08;1.89) | 1.21(0.94;1.55) | 0.03 |
| rs7479949 | 850 | 255 | 319 | 128 | 755 | 292 | 323 | 98 | C | 46 | 1.41(1.09;1.82) |  | 1.27(1.04;1.56) | 1.02(0.78;1.34) | 0.01 |
| rs7930778 | 376 | 119 | 703 | 271 | 393 | 147 | 776 | 236 | T | 42 | 1.33(1.03;1.72) |  | 1.26(0.94;1.68) | 1.05(0.81;1.37) | 0.03 |
| rs7937898 | 392 | 105 | 777 | 278 | 335 | 146 | 744 | 244 | G | 44 | 1.33(1.02;1.74) |  | 1.59(1.17;2.15) | 1.2(0.92;1.57) | 0.01 |
| rs7938923 | 847 | 254 | 322 | 129 | 753 | 289 | 326 | 101 | C | 46 | 1.41(1.09;1.82) |  | 1.27(1.03;1.55) | 1.05(0.8;1.37) | 0.01 |
| rs7944602 | 539 | 138 | 630 | 245 | 460 | 171 | 619 | 219 | A | 35 | 1.43(1.12;1.83) |  | 1.4(1.07;1.82) | 1.29(1;1.65) | 0.03 |
| rs7946015 | 337 | 96 | 832 | 287 | 256 | 122 | 823 | 268 | T | 15 | 1.11(0.85;1.46) |  | 1.51(1.09;2.08) | 1.06(0.81;1.4) | 0.04 |
| rs7947200 | 637 | 175 | 532 | 208 | 512 | 191 | 567 | 199 | T | 29 | 1.43(1.12;1.82) |  | 1.33(1.04;1.7) | 1.25(0.98;1.59) | 0.03 |
| rs893006 | 546 | 156 | 623 | 227 | 472 | 192 | 607 | 198 | C | 34 | 1.25(0.98;1.6) |  | 1.39(1.08;1.79) | 1.1(0.86;1.41) | 0.02 |
| rs897101 | 523 | 149 | 645 | 234 | 476 | 185 | 603 | 205 | C | 33 | 1.36(1.06;1.73) |  | 1.38(1.07;1.79) | 1.21(0.94;1.56) | 0.03 |
| chr 12 |  |  |  |  |  |  |  |  |  |  |  |  |  |  |  |
| rs10082963 | 320 | 95 | 849 | 288 | 245 | 116 | 834 | 274 | T | 14 | 1.1(0.84;1.45) |  | 1.53(1.1;2.14) | 1.05(0.8;1.39) | 0.04 |
| rs1015249 | 556 | 172 | 523 | 218 | 582 | 196 | 587 | 187 | T | 30 | 1.4(1.1;1.78) |  | 1.15(0.9;1.47) | 1.06(0.83;1.36) | 0.03 |
| rs1063856 | 478 | 144 | 601 | 246 | 509 | 174 | 660 | 209 | C | 34 | 1.35(1.06;1.73) |  | 1.17(0.9;1.52) | 1.06(0.83;1.36) | 0.04 |
| rs1063857 | 478 | 145 | 601 | 245 | 509 | 174 | 660 | 209 | G | 34 | 1.34(1.05;1.71) |  | 1.16(0.89;1.51) | 1.06(0.82;1.36) | 0.04 |
| rs10747595 | 547 | 163 | 620 | 220 | 501 | 203 | 578 | 187 | G | 28 | 1.22(0.96;1.55) |  | 1.37(1.07;1.76) | 1.06(0.82;1.36) | 0.02 |
| rs10747607 | 551 | 157 | 618 | 226 | 492 | 198 | 587 | 192 | A | 32 | 1.31(1.02;1.66) |  | 1.37(1.07;1.77) | 1.15(0.89;1.47) | 0.02 |
| rs10773243 | 137 | 36 | 942 | 354 | 133 | 58 | 1036 | 325 | A | 34 | 1.47(0.99;2.2) |  | 1.81(1.1;2.97) | 1.24(0.83;1.86) | 0.04 |
| rs10774048 | 681 | 192 | 488 | 191 | 630 | 240 | 448 | 150 | T | 24 | 1.37(1.07;1.74) |  | 1.33(1.06;1.67) | 1.13(0.88;1.46) | 0.02 |
| rs10774497 | 519 | 133 | 650 | 250 | 453 | 169 | 626 | 221 | G | 35 | 1.5(1.17;1.92) |  | 1.47(1.12;1.92) | 1.31(1.02;1.69) | 0.01 |
| rs10777862 | 539 | 143 | 630 | 240 | 493 | 198 | 585 | 192 | A | 33 | 1.4(1.1;1.79) |  | 1.47(1.14;1.9) | 1.19(0.92;1.54) | 0.01 |
| rs10777981 | 524 | 162 | 555 | 228 | 532 | 189 | 637 | 194 | G | 27 | 1.3(1.02;1.66) |  | 1.15(0.9;1.48) | 1(0.78;1.29) | 0.04 |
| rs10784524 | 629 | 178 | 540 | 205 | 539 | 212 | 540 | 178 | G | 29 | 1.33(1.05;1.69) |  | 1.36(1.07;1.73) | 1.13(0.88;1.44) | 0.02 |
| rs10794472 | 492 | 144 | 587 | 246 | 471 | 173 | 698 | 210 | A | 35 | 1.43(1.12;1.83) |  | 1.26(0.97;1.64) | 1.07(0.83;1.37) | 0.01 |
| rs10842723 | 130 | 32 | 949 | 358 | 95 | 46 | 1072 | 337 | T | 32 | 1.54(1.02;2.34) |  | 1.95(1.14;3.35) | 1.32(0.87;1.99) | 0.04 |
| rs10844690 | 282 | 62 | 887 | 321 | 257 | 106 | 822 | 284 | A | 49 | 1.63(1.19;2.22) |  | 1.85(1.28;2.68) | 1.52(1.11;2.08) | 0.01 |
| rs10845549 | 186 | 53 | 893 | 337 | 170 | 67 | 998 | 316 | A | 39 | 1.38(0.98;1.94) |  | 1.56(1.01;2.41) | 1.16(0.83;1.64) | 0.04 |
| rs10847102 | 136 | 36 | 939 | 354 | 133 | 58 | 1032 | 325 | C | 34 | 1.47(0.98;2.19) |  | 1.8(1.09;2.95) | 1.24(0.83;1.85) | 0.04 |
| rs10847105 | 137 | 36 | 938 | 354 | 133 | 58 | 1034 | 325 | A | 34 | 1.48(0.99;2.21) |  | 1.81(1.1;2.97) | 1.25(0.83;1.86) | 0.04 |
| rs10847107 | 136 | 36 | 940 | 354 | 133 | 58 | 1035 | 325 | A | 34 | 1.47(0.98;2.19) |  | 1.79(1.09;2.94) | 1.23(0.82;1.85) | 0.04 |
| rs10847109 | 137 | 35 | 941 | 354 | 133 | 57 | 1033 | 326 | A | 34 | 1.53(1.02;2.29) |  | 1.85(1.12;3.05) | 1.29(0.86;1.94) | 0.04 |
| rs10847503 | 213 | 55 | 956 | 328 | 169 | 74 | 910 | 316 | C | 42 | 1.35(0.97;1.88) |  | 1.78(1.17;2.69) | 1.32(0.95;1.84) | 0.04 |
| rs10848096 | 753 | 230 | 416 | 153 | 652 | 255 | 427 | 135 | G | 21 | 1.27(0.99;1.63) |  | 1.27(1.02;1.58) | 1.04(0.81;1.34) | 0.03 |
| rs10850369 | 527 | 144 | 642 | 239 | 479 | 186 | 600 | 204 | G | 34 | 1.33(1.04;1.7) |  | 1.36(1.05;1.76) | 1.21(0.94;1.56) | 0.04 |
| rs10861404 | 560 | 160 | 609 | 223 | 469 | 180 | 610 | 210 | C | 33 | 1.32(1.03;1.68) |  | 1.35(1.05;1.75) | 1.19(0.93;1.52) | 0.04 |
| rs11046192 | 990 | 298 | 178 | 85 | 903 | 331 | 176 | 59 | A | 9 | 1.62(1.2;2.2) |  | 1.2(0.99;1.45) | 1.08(0.77;1.51) | 0.02 |
| rs11055443 | 292 | 68 | 877 | 315 | 262 | 106 | 817 | 284 | C | 49 | 1.51(1.12;2.04) |  | 1.67(1.17;2.38) | 1.43(1.06;1.95) | 0.03 |
| rs11055463 | 576 | 148 | 593 | 235 | 512 | 187 | 567 | 203 | G | 32 | 1.47(1.15;1.87) |  | 1.37(1.06;1.77) | 1.31(1.02;1.68) | 0.03 |
| rs11058561 | 137 | 36 | 942 | 354 | 134 | 58 | 1035 | 325 | T | 34 | 1.47(0.99;2.2) |  | 1.79(1.09;2.94) | 1.25(0.83;1.86) | 0.04 |
| rs11059504 | 697 | 208 | 471 | 175 | 629 | 243 | 449 | 146 | T | 36 | 1.21(0.95;1.55) |  | 1.27(1.02;1.59) | 1.03(0.8;1.32) | 0.03 |
| rs11059507 | 696 | 208 | 471 | 175 | 627 | 244 | 448 | 146 | C | 36 | 1.21(0.95;1.54) |  | 1.28(1.03;1.6) | 1.03(0.8;1.33) | 0.03 |
| rs11060979 | 186 | 51 | 893 | 339 | 188 | 82 | 981 | 301 | T | 9 | 1.43(1.01;2.03) |  | 1.67(1.09;2.55) | 1.18(0.83;1.67) | 0.03 |
| rs11061371 | 284 | 81 | 885 | 302 | 228 | 109 | 851 | 281 | A | 12 | 1.23(0.92;1.64) |  | 1.7(1.2;2.41) | 1.15(0.86;1.53) | 0.02 |
| rs11114085 | 411 | 121 | 758 | 262 | 358 | 151 | 721 | 239 | C | 19 | 1.21(0.93;1.56) |  | 1.42(1.07;1.9) | 1.12(0.87;1.45) | 0.04 |
| rs11114086 | 411 | 121 | 758 | 262 | 358 | 151 | 721 | 239 | T | 19 | 1.21(0.94;1.56) |  | 1.42(1.07;1.9) | 1.12(0.87;1.46) | 0.04 |
| rs11168267 | 241 | 70 | 829 | 317 | 226 | 92 | 930 | 290 | A | 11 | 1.38(1.01;1.87) |  | 1.45(1;2.1) | 1.14(0.84;1.55) | 0.03 |
| rs11180235 | 764 | 227 | 404 | 156 | 671 | 260 | 407 | 129 | T | 20 | 1.36(1.06;1.74) |  | 1.29(1.04;1.6) | 1.07(0.83;1.39) | 0.01 |
| rs1147106 | 457 | 143 | 712 | 240 | 413 | 174 | 666 | 216 | G | 23 | 1.12(0.88;1.44) |  | 1.37(1.05;1.8) | 1.03(0.8;1.33) | 0.04 |
| rs1148412 | 224 | 63 | 855 | 326 | 221 | 91 | 948 | 292 | T | 45 | 1.34(0.98;1.84) |  | 1.48(1.01;2.18) | 1.11(0.8;1.53) | 0.03 |
| rs11574113 | 888 | 257 | 281 | 126 | 805 | 292 | 274 | 98 | G | 14 | 1.49(1.14;1.93) |  | 1.22(1;1.5) | 1.16(0.88;1.54) | 0.04 |
| rs11608334 | 816 | 246 | 353 | 137 | 690 | 260 | 389 | 130 | G | 19 | 1.3(1.01;1.68) |  | 1.26(1.02;1.55) | 1.05(0.81;1.35) | 0.03 |
| rs1169077 | 852 | 250 | 317 | 133 | 790 | 293 | 289 | 97 | A | 15 | 1.47(1.14;1.9) |  | 1.25(1.02;1.53) | 1.12(0.85;1.49) | 0.02 |
| rs1181334 | 818 | 231 | 350 | 152 | 707 | 262 | 371 | 127 | T | 18 | 1.57(1.22;2.01) |  | 1.3(1.05;1.61) | 1.18(0.91;1.53) | 0.01 |
| rs12315279 | 178 | 54 | 901 | 336 | 184 | 85 | 985 | 298 | T | 9 | 1.26(0.89;1.78) |  | 1.58(1.04;2.41) | 1.04(0.74;1.48) | 0.04 |
| rs12318183 | 458 | 144 | 621 | 246 | 494 | 175 | 675 | 208 | A | 36 | 1.31(1.02;1.68) |  | 1.18(0.9;1.53) | 1.03(0.8;1.32) | 0.04 |
| rs12370197 | 455 | 125 | 714 | 258 | 406 | 160 | 673 | 229 | C | 38 | 1.28(1;1.65) |  | 1.41(1.06;1.86) | 1.18(0.91;1.52) | 0.04 |
| rs12372629 | 730 | 224 | 439 | 159 | 644 | 254 | 435 | 136 | T | 22 | 1.28(1.01;1.64) |  | 1.3(1.05;1.62) | 1.03(0.8;1.33) | 0.01 |
| rs12423724 | 137 | 36 | 942 | 354 | 133 | 58 | 1036 | 325 | G | 34 | 1.47(0.99;2.2) |  | 1.81(1.1;2.97) | 1.24(0.83;1.86) | 0.04 |
| rs12423726 | 137 | 36 | 942 | 354 | 135 | 58 | 1034 | 325 | G | 34 | 1.47(0.99;2.2) |  | 1.79(1.09;2.93) | 1.25(0.83;1.86) | 0.04 |
| rs12717991 | 445 | 134 | 634 | 256 | 404 | 139 | 765 | 244 | T | 40 | 1.39(1.08;1.78) |  | 1.21(0.91;1.6) | 1.1(0.85;1.41) | 0.04 |
| rs1421454 | 313 | 92 | 766 | 298 | 264 | 103 | 905 | 280 | G | 50 | 1.38(1.05;1.83) |  | 1.42(1.01;1.99) | 1.11(0.84;1.47) | 0.02 |
| rs1476798 | 137 | 36 | 942 | 354 | 133 | 58 | 1036 | 325 | G | 34 | 1.47(0.99;2.2) |  | 1.81(1.1;2.97) | 1.24(0.83;1.86) | 0.04 |
| rs1480625 | 538 | 158 | 631 | 225 | 445 | 183 | 634 | 207 | T | 26 | 1.2(0.95;1.53) |  | 1.38(1.07;1.78) | 1.07(0.84;1.37) | 0.03 |
| rs17045649 | 819 | 247 | 350 | 136 | 690 | 262 | 389 | 128 | A | 19 | 1.31(1.02;1.68) |  | 1.27(1.03;1.56) | 1.03(0.8;1.33) | 0.02 |
| rs17188203 | 651 | 199 | 518 | 184 | 562 | 220 | 517 | 170 | T | 26 | 1.24(0.98;1.58) |  | 1.3(1.03;1.63) | 1.08(0.84;1.38) | 0.04 |
| rs17313592 | 204 | 62 | 872 | 326 | 193 | 86 | 974 | 297 | G | 10 | 1.21(0.88;1.67) |  | 1.55(1.05;2.3) | 1(0.73;1.38) | 0.03 |
| rs17723777 | 320 | 88 | 849 | 295 | 279 | 120 | 800 | 270 | A | 14 | 1.32(1;1.76) |  | 1.65(1.18;2.31) | 1.23(0.92;1.63) | 0.02 |
| rs17767008 | 290 | 82 | 879 | 301 | 236 | 107 | 843 | 283 | T | 13 | 1.2(0.9;1.6) |  | 1.56(1.1;2.21) | 1.15(0.86;1.54) | 0.04 |
| rs1798682 | 737 | 221 | 432 | 162 | 697 | 263 | 382 | 127 | G | 40 | 1.27(0.99;1.62) |  | 1.25(1.01;1.55) | 1.07(0.82;1.38) | 0.04 |
| rs1842599 | 395 | 103 | 759 | 276 | 358 | 142 | 709 | 247 | G | 42 | 1.38(1.06;1.8) |  | 1.49(1.1;2.02) | 1.29(0.99;1.69) | 0.03 |
| rs1862013 | 592 | 155 | 577 | 228 | 502 | 178 | 577 | 212 | C | 32 | 1.53(1.2;1.95) |  | 1.38(1.07;1.78) | 1.34(1.05;1.71) | 0.02 |
| rs1994474 | 242 | 68 | 837 | 322 | 276 | 104 | 893 | 279 | C | 48 | 1.4(1.03;1.9) |  | 1.41(0.98;2.03) | 1.15(0.84;1.57) | 0.04 |
| rs2129029 | 524 | 151 | 645 | 232 | 436 | 178 | 642 | 211 | T | 24 | 1.32(1.03;1.69) |  | 1.42(1.09;1.84) | 1.16(0.9;1.49) | 0.02 |
| rs2160525 | 800 | 237 | 369 | 146 | 704 | 259 | 375 | 131 | A | 19 | 1.41(1.1;1.81) |  | 1.25(1.01;1.55) | 1.15(0.89;1.49) | 0.03 |
| rs2165039 | 599 | 168 | 570 | 215 | 525 | 199 | 554 | 191 | T | 30 | 1.39(1.09;1.77) |  | 1.37(1.07;1.75) | 1.2(0.94;1.54) | 0.02 |
| rs2193041 | 459 | 144 | 620 | 246 | 495 | 175 | 674 | 208 | G | 36 | 1.31(1.03;1.68) |  | 1.18(0.9;1.53) | 1.03(0.8;1.33) | 0.04 |
| rs2238104 | 346 | 101 | 733 | 289 | 335 | 118 | 834 | 265 | T | 45 | 1.41(1.08;1.84) |  | 1.28(0.93;1.76) | 1.14(0.87;1.5) | 0.04 |
| rs2239186 | 423 | 123 | 746 | 260 | 359 | 158 | 720 | 232 | G | 20 | 1.2(0.93;1.55) |  | 1.5(1.13;1.99) | 1.08(0.83;1.4) | 0.02 |
| rs2264420 | 259 | 74 | 820 | 316 | 284 | 116 | 885 | 267 | G | 13 | 1.38(1.02;1.86) |  | 1.43(1.01;2.03) | 1.12(0.82;1.51) | 0.03 |
| rs2280684 | 245 | 61 | 924 | 322 | 194 | 89 | 885 | 301 | A | 44 | 1.35(0.98;1.86) |  | 1.7(1.16;2.52) | 1.31(0.95;1.8) | 0.04 |
| rs2283342 | 361 | 107 | 808 | 276 | 307 | 139 | 772 | 251 | G | 17 | 1.18(0.9;1.53) |  | 1.55(1.14;2.1) | 1.08(0.83;1.41) | 0.02 |
| rs2287155 | 287 | 92 | 792 | 298 | 247 | 99 | 922 | 284 | A | 13 | 1.28(0.97;1.7) |  | 1.45(1.03;2.05) | 1.03(0.78;1.36) | 0.02 |
| rs2287538 | 414 | 110 | 755 | 273 | 354 | 134 | 725 | 256 | C | 42 | 1.46(1.12;1.89) |  | 1.54(1.14;2.08) | 1.32(1.01;1.71) | 0.02 |
| rs2289768 | 381 | 110 | 698 | 280 | 405 | 144 | 764 | 239 | T | 42 | 1.52(1.17;1.97) |  | 1.34(1;1.81) | 1.17(0.89;1.53) | 0.01 |
| rs27633 | 400 | 122 | 679 | 268 | 403 | 147 | 766 | 236 | G | 41 | 1.27(0.99;1.64) |  | 1.23(0.92;1.64) | 1.01(0.78;1.31) | 0.04 |
| rs2883575 | 362 | 107 | 717 | 283 | 377 | 136 | 792 | 247 | A | 43 | 1.31(1.01;1.7) |  | 1.27(0.94;1.72) | 1.05(0.8;1.37) | 0.03 |
| rs2888604 | 329 | 100 | 750 | 290 | 282 | 108 | 887 | 275 | T | 49 | 1.28(0.98;1.68) |  | 1.33(0.96;1.84) | 1.04(0.79;1.37) | 0.03 |
| rs289061 | 664 | 182 | 505 | 201 | 567 | 224 | 512 | 166 | T | 27 | 1.48(1.16;1.88) |  | 1.42(1.12;1.8) | 1.16(0.91;1.5) | 0.00 |
| rs34529 | 524 | 155 | 645 | 228 | 466 | 188 | 613 | 202 | T | 33 | 1.23(0.97;1.57) |  | 1.4(1.08;1.81) | 1.09(0.85;1.4) | 0.02 |
| rs3782587 | 919 | 277 | 250 | 106 | 847 | 307 | 232 | 83 | T | 12 | 1.47(1.12;1.93) |  | 1.21(0.99;1.47) | 1.12(0.84;1.51) | 0.04 |
| rs3847987 | 839 | 237 | 330 | 146 | 750 | 283 | 329 | 107 | A | 17 | 1.51(1.17;1.94) |  | 1.3(1.06;1.6) | 1.09(0.83;1.43) | 0.00 |
| rs3907505 | 371 | 110 | 708 | 280 | 388 | 138 | 781 | 245 | T | 42 | 1.32(1.01;1.71) |  | 1.26(0.93;1.7) | 1.05(0.81;1.37) | 0.03 |
| rs3923249 | 343 | 99 | 826 | 284 | 284 | 129 | 793 | 261 | G | 15 | 1.24(0.94;1.62) |  | 1.65(1.2;2.26) | 1.13(0.86;1.48) | 0.01 |
| rs403734 | 97 | 24 | 1052 | 357 | 67 | 44 | 1008 | 343 | G | 27 | 1.37(0.85;2.19) |  | 2.63(1.44;4.79) | 1.34(0.83;2.16) | 0.04 |
| rs4293213 | 386 | 106 | 783 | 277 | 313 | 130 | 766 | 260 | T | 45 | 1.35(1.03;1.76) |  | 1.55(1.14;2.11) | 1.24(0.95;1.62) | 0.02 |
| rs4612892 | 478 | 149 | 691 | 234 | 404 | 180 | 675 | 210 | C | 23 | 1.12(0.87;1.43) |  | 1.39(1.07;1.81) | 1.01(0.79;1.29) | 0.03 |
| rs4760543 | 1029 | 318 | 140 | 65 | 904 | 333 | 175 | 57 | T | 7 | 1.55(1.11;2.17) |  | 1.17(0.97;1.41) | 1.02(0.73;1.42) | 0.03 |
| rs4765339 | 137 | 36 | 942 | 354 | 133 | 58 | 1036 | 325 | A | 34 | 1.47(0.99;2.2) |  | 1.81(1.1;2.97) | 1.24(0.83;1.86) | 0.04 |
| rs626979 | 963 | 294 | 206 | 89 | 858 | 318 | 221 | 72 | G | 10 | 1.43(1.06;1.91) |  | 1.19(0.99;1.45) | 1.04(0.76;1.41) | 0.03 |
| rs7137457 | 137 | 36 | 942 | 354 | 133 | 58 | 1036 | 325 | C | 34 | 1.47(0.99;2.2) |  | 1.81(1.1;2.97) | 1.24(0.83;1.86) | 0.04 |
| rs7138407 | 459 | 144 | 620 | 246 | 495 | 175 | 674 | 208 | A | 36 | 1.31(1.03;1.68) |  | 1.18(0.9;1.53) | 1.03(0.8;1.33) | 0.04 |
| rs7294867 | 137 | 36 | 940 | 352 | 134 | 58 | 1030 | 323 | T | 34 | 1.47(0.98;2.2) |  | 1.8(1.1;2.95) | 1.24(0.83;1.86) | 0.04 |
| rs7311151 | 386 | 104 | 783 | 279 | 340 | 135 | 739 | 255 | G | 44 | 1.35(1.04;1.76) |  | 1.48(1.09;2) | 1.27(0.97;1.66) | 0.04 |
| rs7486184 | 690 | 207 | 479 | 176 | 632 | 238 | 447 | 152 | A | 24 | 1.26(0.99;1.6) |  | 1.27(1.02;1.59) | 1.08(0.84;1.39) | 0.04 |
| rs772000 | 593 | 188 | 576 | 195 | 531 | 215 | 548 | 175 | C | 30 | 1.15(0.91;1.47) |  | 1.32(1.04;1.67) | 1.01(0.79;1.3) | 0.03 |
| rs774039 | 367 | 115 | 712 | 275 | 350 | 133 | 819 | 250 | G | 44 | 1.29(0.99;1.67) |  | 1.25(0.92;1.68) | 1.04(0.8;1.35) | 0.04 |
| rs7964127 | 512 | 134 | 657 | 249 | 434 | 162 | 645 | 228 | T | 36 | 1.45(1.13;1.85) |  | 1.42(1.08;1.86) | 1.3(1.01;1.67) | 0.03 |
| rs7964826 | 379 | 110 | 790 | 273 | 316 | 136 | 763 | 254 | A | 17 | 1.19(0.92;1.55) |  | 1.5(1.11;2.03) | 1.11(0.85;1.45) | 0.03 |
| rs7967352 | 137 | 36 | 942 | 354 | 133 | 58 | 1036 | 325 | T | 34 | 1.47(0.99;2.2) |  | 1.81(1.1;2.97) | 1.24(0.83;1.86) | 0.04 |
| rs7967559 | 819 | 247 | 350 | 136 | 690 | 261 | 389 | 129 | A | 19 | 1.31(1.02;1.68) |  | 1.26(1.02;1.55) | 1.04(0.81;1.34) | 0.02 |
| rs7970953 | 134 | 25 | 1035 | 358 | 111 | 46 | 968 | 344 | G | 32 | 2.02(1.27;3.2) |  | 2.5(1.42;4.4) | 1.99(1.26;3.17) | 0.04 |
| rs7972971 | 668 | 200 | 498 | 183 | 618 | 242 | 461 | 148 | G | 35 | 1.22(0.96;1.55) |  | 1.3(1.03;1.62) | 1.02(0.79;1.31) | 0.02 |
| rs7976512 | 565 | 171 | 600 | 211 | 490 | 198 | 588 | 190 | G | 28 | 1.23(0.96;1.56) |  | 1.34(1.05;1.71) | 1.08(0.84;1.38) | 0.03 |
| rs7977889 | 136 | 36 | 943 | 354 | 128 | 58 | 1040 | 325 | C | 33 | 1.46(0.98;2.18) |  | 1.86(1.13;3.06) | 1.23(0.82;1.84) | 0.04 |
| rs998377 | 511 | 139 | 658 | 244 | 456 | 184 | 623 | 206 | A | 35 | 1.33(1.04;1.71) |  | 1.43(1.1;1.87) | 1.17(0.91;1.51) | 0.02 |
| chr 13 |  |  |  |  |  |  |  |  |  |  |  |  |  |  |  |
| rs11841946 | 345 | 103 | 734 | 287 | 354 | 130 | 815 | 253 | A | 45 | 1.38(1.06;1.81) |  | 1.31(0.96;1.78) | 1.1(0.84;1.45) | 0.03 |
| rs12020228 | 883 | 255 | 286 | 128 | 742 | 282 | 337 | 108 | T | 15 | 1.54(1.19;2) |  | 1.3(1.06;1.6) | 1.06(0.81;1.38) | 0.00 |
| rs12021161 | 800 | 233 | 369 | 150 | 665 | 257 | 414 | 133 | C | 20 | 1.45(1.13;1.86) |  | 1.33(1.07;1.65) | 1.08(0.84;1.39) | 0.00 |
| rs12323080 | 875 | 250 | 294 | 133 | 759 | 288 | 319 | 102 | C | 15 | 1.56(1.2;2.02) |  | 1.31(1.07;1.6) | 1.06(0.8;1.39) | 0.00 |
| rs1409570 | 745 | 215 | 424 | 168 | 691 | 264 | 388 | 126 | C | 20 | 1.41(1.1;1.8) |  | 1.31(1.05;1.62) | 1.11(0.85;1.44) | 0.01 |
| rs17086822 | 227 | 65 | 942 | 318 | 188 | 102 | 890 | 288 | A | 10 | 1.21(0.89;1.66) |  | 1.85(1.27;2.71) | 1.14(0.83;1.57) | 0.02 |
| rs17716584 | 446 | 121 | 723 | 262 | 409 | 160 | 670 | 230 | A | 39 | 1.39(1.08;1.79) |  | 1.44(1.09;1.91) | 1.27(0.98;1.64) | 0.03 |
| rs1926447 | 105 | 21 | 1060 | 361 | 74 | 40 | 1004 | 350 | G | 28 | 1.67(1.02;2.73) |  | 2.81(1.51;5.24) | 1.66(1.01;2.72) | 0.04 |
| rs1927850 | 377 | 92 | 792 | 291 | 340 | 133 | 739 | 257 | A | 45 | 1.55(1.17;2.04) |  | 1.67(1.22;2.29) | 1.4(1.06;1.85) | 0.01 |
| rs2182493 | 396 | 119 | 773 | 264 | 369 | 153 | 710 | 237 | A | 19 | 1.21(0.93;1.57) |  | 1.43(1.07;1.91) | 1.13(0.86;1.47) | 0.04 |
| rs2325219 | 425 | 115 | 744 | 268 | 352 | 142 | 727 | 248 | C | 42 | 1.3(1;1.68) |  | 1.48(1.1;1.98) | 1.2(0.92;1.55) | 0.03 |
| rs2875652 | 431 | 129 | 738 | 254 | 381 | 162 | 698 | 228 | C | 21 | 1.17(0.91;1.51) |  | 1.45(1.1;1.92) | 1.06(0.82;1.37) | 0.02 |
| rs3099362 | 830 | 272 | 249 | 118 | 874 | 285 | 294 | 98 | C | 13 | 1.57(1.2;2.05) |  | 1.04(0.85;1.26) | 1.07(0.81;1.41) | 0.04 |
| rs3888918 | 415 | 121 | 664 | 269 | 430 | 147 | 739 | 236 | G | 40 | 1.46(1.13;1.89) |  | 1.28(0.96;1.7) | 1.13(0.87;1.47) | 0.02 |
| rs4769524 | 992 | 296 | 177 | 87 | 914 | 330 | 165 | 60 | T | 9 | 1.6(1.19;2.16) |  | 1.19(0.99;1.44) | 1.12(0.8;1.56) | 0.03 |
| rs6314 | 188 | 56 | 891 | 334 | 167 | 79 | 1002 | 304 | A | 8 | 1.26(0.9;1.76) |  | 1.6(1.06;2.44) | 1.05(0.75;1.47) | 0.03 |
| rs7318912 | 263 | 80 | 816 | 310 | 245 | 107 | 924 | 276 | A | 12 | 1.26(0.94;1.69) |  | 1.49(1.05;2.11) | 1.01(0.75;1.35) | 0.02 |
| rs7321236 | 642 | 181 | 527 | 202 | 578 | 218 | 501 | 172 | C | 27 | 1.36(1.07;1.73) |  | 1.31(1.04;1.66) | 1.19(0.93;1.53) | 0.04 |
| rs7324312 | 574 | 156 | 595 | 227 | 532 | 199 | 547 | 191 | G | 31 | 1.43(1.12;1.82) |  | 1.35(1.05;1.73) | 1.28(0.99;1.64) | 0.04 |
| rs7328971 | 655 | 199 | 514 | 184 | 577 | 224 | 502 | 166 | T | 27 | 1.19(0.94;1.51) |  | 1.29(1.02;1.62) | 1.04(0.81;1.33) | 0.04 |
| rs7333197 | 381 | 120 | 698 | 270 | 370 | 140 | 799 | 243 | T | 19 | 1.27(0.98;1.64) |  | 1.25(0.93;1.68) | 1.01(0.78;1.32) | 0.03 |
| rs7338915 | 648 | 187 | 521 | 196 | 558 | 215 | 521 | 175 | A | 28 | 1.33(1.05;1.7) |  | 1.34(1.06;1.7) | 1.13(0.88;1.44) | 0.02 |
| rs7787 | 288 | 72 | 787 | 318 | 317 | 103 | 851 | 279 | A | 50 | 1.61(1.2;2.17) |  | 1.41(0.99;2) | 1.31(0.97;1.77) | 0.02 |
| rs7983595 | 565 | 177 | 604 | 206 | 497 | 208 | 582 | 182 | C | 28 | 1.12(0.88;1.42) |  | 1.31(1.03;1.68) | 1(0.78;1.28) | 0.04 |
| rs7999161 | 882 | 255 | 287 | 128 | 742 | 282 | 337 | 108 | G | 15 | 1.53(1.18;1.99) |  | 1.3(1.06;1.59) | 1.05(0.81;1.38) | 0.00 |
| rs816142 | 825 | 270 | 254 | 120 | 873 | 289 | 296 | 94 | A | 13 | 1.57(1.21;2.05) |  | 1.05(0.86;1.28) | 1.04(0.78;1.37) | 0.02 |
| rs9316636 | 297 | 82 | 872 | 301 | 247 | 106 | 832 | 283 | G | 48 | 1.31(0.98;1.74) |  | 1.59(1.12;2.24) | 1.25(0.93;1.67) | 0.04 |
| rs9506622 | 767 | 226 | 402 | 157 | 641 | 255 | 438 | 135 | A | 21 | 1.3(1.02;1.66) |  | 1.3(1.05;1.61) | 1.03(0.8;1.33) | 0.01 |
| rs9522034 | 673 | 195 | 496 | 188 | 539 | 216 | 540 | 174 | A | 27 | 1.34(1.06;1.71) |  | 1.37(1.08;1.73) | 1.11(0.87;1.41) | 0.01 |
| rs9522099 | 295 | 88 | 782 | 302 | 269 | 117 | 898 | 266 | G | 14 | 1.32(0.99;1.74) |  | 1.52(1.09;2.12) | 1.02(0.77;1.36) | 0.01 |
| rs9525560 | 314 | 80 | 854 | 303 | 275 | 107 | 804 | 283 | C | 49 | 1.48(1.1;1.97) |  | 1.61(1.14;2.28) | 1.4(1.04;1.87) | 0.03 |
| rs9559794 | 877 | 264 | 292 | 119 | 790 | 288 | 289 | 102 | G | 14 | 1.36(1.05;1.77) |  | 1.22(1;1.5) | 1.06(0.8;1.39) | 0.03 |
| rs9568944 | 309 | 88 | 860 | 295 | 259 | 110 | 820 | 280 | A | 14 | 1.27(0.96;1.69) |  | 1.61(1.14;2.26) | 1.2(0.9;1.6) | 0.03 |
| rs9572452 | 777 | 238 | 392 | 145 | 715 | 277 | 364 | 113 | C | 18 | 1.24(0.97;1.59) |  | 1.25(1.01;1.54) | 1(0.77;1.31) | 0.03 |
| rs9593305 | 249 | 77 | 830 | 313 | 241 | 103 | 928 | 280 | T | 12 | 1.24(0.93;1.67) |  | 1.4(0.98;2) | 1.02(0.76;1.38) | 0.04 |
| rs9634641 | 936 | 273 | 233 | 110 | 828 | 302 | 251 | 88 | C | 12 | 1.61(1.22;2.11) |  | 1.22(1;1.48) | 1.18(0.88;1.58) | 0.02 |
| chr 14 |  |  |  |  |  |  |  |  |  |  |  |  |  |  |  |
| rs10131232 | 142 | 36 | 937 | 354 | 140 | 64 | 1029 | 319 | G | 34 | 1.52(1.02;2.28) |  | 1.91(1.17;3.12) | 1.27(0.85;1.91) | 0.03 |
| rs10146997 | 781 | 229 | 388 | 154 | 690 | 255 | 389 | 135 | G | 19 | 1.36(1.06;1.74) |  | 1.26(1.02;1.56) | 1.12(0.87;1.45) | 0.03 |
| rs11627269 | 543 | 150 | 626 | 233 | 472 | 179 | 607 | 211 | T | 34 | 1.36(1.07;1.74) |  | 1.38(1.07;1.79) | 1.22(0.95;1.56) | 0.03 |
| rs11627633 | 309 | 84 | 860 | 299 | 282 | 118 | 797 | 272 | T | 49 | 1.32(0.99;1.75) |  | 1.6(1.14;2.24) | 1.24(0.93;1.65) | 0.03 |
| rs11627827 | 933 | 285 | 236 | 98 | 838 | 314 | 240 | 76 | A | 11 | 1.38(1.04;1.83) |  | 1.2(0.99;1.46) | 1.04(0.77;1.41) | 0.04 |
| rs11847091 | 599 | 172 | 570 | 211 | 522 | 210 | 557 | 180 | T | 29 | 1.29(1.01;1.64) |  | 1.4(1.1;1.79) | 1.08(0.84;1.38) | 0.01 |
| rs1187628 | 249 | 67 | 830 | 323 | 231 | 81 | 938 | 302 | T | 46 | 1.5(1.1;2.05) |  | 1.42(0.97;2.09) | 1.25(0.92;1.7) | 0.04 |
| rs1188177 | 322 | 79 | 847 | 304 | 253 | 102 | 826 | 288 | T | 50 | 1.43(1.07;1.91) |  | 1.58(1.11;2.23) | 1.37(1.02;1.83) | 0.04 |
| rs1191355 | 742 | 219 | 427 | 164 | 647 | 251 | 432 | 139 | T | 22 | 1.25(0.98;1.59) |  | 1.26(1.01;1.57) | 1.06(0.82;1.36) | 0.04 |
| rs1191385 | 744 | 220 | 425 | 163 | 643 | 255 | 436 | 135 | T | 22 | 1.24(0.97;1.58) |  | 1.28(1.03;1.59) | 1.01(0.79;1.3) | 0.02 |
| rs1191386 | 649 | 183 | 520 | 200 | 553 | 217 | 526 | 173 | A | 27 | 1.31(1.03;1.67) |  | 1.34(1.06;1.69) | 1.12(0.88;1.43) | 0.02 |
| rs1241486 | 351 | 99 | 818 | 284 | 269 | 119 | 810 | 271 | G | 47 | 1.21(0.93;1.59) |  | 1.55(1.12;2.14) | 1.14(0.87;1.5) | 0.03 |
| rs12431626 | 675 | 203 | 494 | 180 | 596 | 238 | 483 | 152 | A | 34 | 1.18(0.93;1.5) |  | 1.28(1.02;1.6) | 1.02(0.8;1.31) | 0.04 |
| rs1243164 | 709 | 207 | 460 | 176 | 580 | 230 | 499 | 160 | A | 25 | 1.27(1;1.62) |  | 1.29(1.03;1.62) | 1.09(0.85;1.39) | 0.03 |
| rs12435212 | 577 | 168 | 592 | 215 | 501 | 199 | 578 | 191 | G | 31 | 1.32(1.04;1.68) |  | 1.35(1.05;1.72) | 1.17(0.91;1.5) | 0.03 |
| rs12589312 | 651 | 194 | 518 | 189 | 598 | 228 | 481 | 162 | T | 33 | 1.33(1.04;1.69) |  | 1.29(1.03;1.63) | 1.15(0.9;1.48) | 0.04 |
| rs12887555 | 260 | 72 | 909 | 311 | 226 | 96 | 853 | 294 | G | 12 | 1.28(0.94;1.73) |  | 1.66(1.14;2.4) | 1.23(0.91;1.67) | 0.04 |
| rs12893432 | 128 | 24 | 1041 | 359 | 121 | 63 | 958 | 327 | T | 6 | 1.7(1.07;2.69) |  | 2.62(1.52;4.53) | 1.65(1.04;2.62) | 0.03 |
| rs1400745 | 264 | 71 | 815 | 319 | 282 | 103 | 887 | 280 | G | 49 | 1.45(1.08;1.96) |  | 1.38(0.96;1.97) | 1.2(0.89;1.63) | 0.04 |
| rs1465643 | 274 | 82 | 805 | 308 | 269 | 110 | 900 | 273 | T | 49 | 1.29(0.96;1.71) |  | 1.43(1.01;2.01) | 1.04(0.77;1.39) | 0.02 |
| rs1642866 | 837 | 250 | 332 | 133 | 769 | 286 | 310 | 104 | A | 16 | 1.35(1.05;1.75) |  | 1.24(1.01;1.53) | 1.05(0.8;1.38) | 0.02 |
| rs17109256 | 784 | 230 | 385 | 153 | 694 | 255 | 385 | 135 | A | 19 | 1.36(1.06;1.74) |  | 1.25(1.01;1.55) | 1.13(0.88;1.46) | 0.04 |
| rs17616128 | 708 | 216 | 461 | 167 | 630 | 241 | 449 | 149 | T | 23 | 1.31(1.03;1.67) |  | 1.27(1.02;1.58) | 1.12(0.87;1.43) | 0.04 |
| rs1812181 | 818 | 239 | 351 | 144 | 720 | 279 | 359 | 111 | T | 18 | 1.38(1.07;1.77) |  | 1.29(1.05;1.59) | 1.02(0.78;1.33) | 0.01 |
| rs181455 | 172 | 42 | 907 | 348 | 184 | 69 | 985 | 314 | T | 39 | 1.55(1.07;2.24) |  | 1.63(1.04;2.56) | 1.3(0.9;1.89) | 0.04 |
| rs1950996 | 258 | 62 | 911 | 321 | 213 | 93 | 866 | 297 | G | 44 | 1.45(1.05;1.98) |  | 1.78(1.21;2.6) | 1.38(1;1.89) | 0.03 |
| rs1956175 | 686 | 199 | 483 | 184 | 580 | 226 | 499 | 164 | A | 25 | 1.34(1.05;1.7) |  | 1.3(1.04;1.64) | 1.15(0.9;1.47) | 0.03 |
| rs1958664 | 333 | 86 | 832 | 297 | 265 | 109 | 813 | 281 | G | 49 | 1.35(1.02;1.8) |  | 1.54(1.1;2.16) | 1.29(0.97;1.72) | 0.04 |
| rs2149841 | 258 | 76 | 821 | 314 | 235 | 93 | 934 | 290 | C | 47 | 1.23(0.92;1.66) |  | 1.39(0.96;1.99) | 1.02(0.76;1.38) | 0.04 |
| rs221898 | 351 | 103 | 728 | 287 | 364 | 135 | 803 | 248 | C | 44 | 1.26(0.96;1.64) |  | 1.27(0.93;1.72) | 1.01(0.77;1.33) | 0.04 |
| rs221899 | 636 | 198 | 533 | 185 | 577 | 233 | 501 | 157 | A | 33 | 1.18(0.93;1.51) |  | 1.31(1.04;1.65) | 1.01(0.79;1.29) | 0.02 |
| rs221903 | 440 | 135 | 639 | 255 | 456 | 157 | 713 | 226 | T | 37 | 1.31(1.02;1.68) |  | 1.19(0.9;1.56) | 1.04(0.8;1.33) | 0.04 |
| rs222629 | 228 | 57 | 851 | 332 | 240 | 85 | 929 | 298 | C | 44 | 1.65(1.19;2.29) |  | 1.55(1.04;2.29) | 1.36(0.98;1.9) | 0.03 |
| rs2526856 | 344 | 100 | 735 | 290 | 359 | 131 | 809 | 252 | C | 45 | 1.28(0.98;1.68) |  | 1.27(0.93;1.73) | 1.04(0.79;1.37) | 0.04 |
| rs2616774 | 960 | 286 | 209 | 97 | 824 | 301 | 255 | 89 | A | 11 | 1.58(1.19;2.1) |  | 1.22(1;1.48) | 1.12(0.84;1.49) | 0.02 |
| rs3814871 | 653 | 204 | 516 | 179 | 592 | 235 | 487 | 155 | A | 34 | 1.18(0.93;1.5) |  | 1.28(1.02;1.61) | 1.02(0.79;1.3) | 0.04 |
| rs712311 | 367 | 103 | 712 | 287 | 368 | 142 | 801 | 241 | G | 42 | 1.35(1.03;1.76) |  | 1.36(1;1.84) | 1.04(0.8;1.36) | 0.01 |
| rs7144011 | 784 | 230 | 385 | 153 | 693 | 255 | 386 | 135 | T | 19 | 1.36(1.06;1.74) |  | 1.25(1.01;1.55) | 1.13(0.87;1.45) | 0.04 |
| rs7149774 | 443 | 119 | 726 | 264 | 388 | 146 | 691 | 244 | G | 40 | 1.44(1.12;1.86) |  | 1.43(1.07;1.91) | 1.33(1.03;1.73) | 0.04 |
| rs808232 | 677 | 193 | 491 | 190 | 597 | 228 | 482 | 162 | C | 25 | 1.3(1.03;1.66) |  | 1.3(1.03;1.63) | 1.12(0.87;1.43) | 0.03 |
| rs8176373 | 700 | 214 | 469 | 169 | 626 | 243 | 453 | 147 | A | 24 | 1.18(0.93;1.51) |  | 1.27(1.02;1.59) | 1(0.78;1.29) | 0.03 |
| rs860328 | 595 | 181 | 573 | 202 | 482 | 195 | 597 | 195 | A | 28 | 1.18(0.93;1.5) |  | 1.33(1.04;1.7) | 1.05(0.82;1.33) | 0.04 |
| rs912854 | 418 | 128 | 751 | 255 | 399 | 179 | 680 | 211 | C | 21 | 1.12(0.87;1.45) |  | 1.41(1.07;1.86) | 1.02(0.78;1.32) | 0.03 |
| rs912857 | 238 | 59 | 931 | 324 | 208 | 87 | 871 | 303 | T | 45 | 1.47(1.06;2.03) |  | 1.78(1.2;2.63) | 1.41(1.02;1.95) | 0.03 |
| rs917065 | 654 | 204 | 515 | 179 | 592 | 235 | 487 | 155 | G | 34 | 1.18(0.93;1.5) |  | 1.28(1.02;1.61) | 1.02(0.79;1.31) | 0.04 |
| rs9743657 | 810 | 264 | 269 | 125 | 834 | 282 | 334 | 101 | T | 15 | 1.45(1.11;1.88) |  | 1.04(0.85;1.27) | 1(0.76;1.32) | 0.04 |
| chr 15 |  |  |  |  |  |  |  |  |  |  |  |  |  |  |  |
| rs10438342 | 501 | 144 | 578 | 246 | 487 | 165 | 682 | 218 | A | 35 | 1.5(1.17;1.92) |  | 1.21(0.93;1.58) | 1.14(0.89;1.47) | 0.02 |
| rs10775211 | 231 | 55 | 938 | 328 | 160 | 65 | 917 | 325 | G | 41 | 1.64(1.17;2.29) |  | 1.86(1.21;2.85) | 1.59(1.14;2.22) | 0.04 |
| rs11632307 | 607 | 182 | 561 | 201 | 540 | 213 | 537 | 177 | A | 29 | 1.22(0.96;1.55) |  | 1.36(1.07;1.72) | 1.04(0.81;1.33) | 0.02 |
| rs11632441 | 282 | 74 | 797 | 316 | 267 | 94 | 901 | 289 | G | 47 | 1.5(1.11;2.01) |  | 1.36(0.95;1.96) | 1.24(0.92;1.67) | 0.04 |
| rs11633785 | 326 | 90 | 842 | 293 | 262 | 113 | 817 | 277 | C | 49 | 1.24(0.94;1.64) |  | 1.54(1.1;2.14) | 1.18(0.89;1.57) | 0.04 |
| rs11636496 | 160 | 39 | 919 | 351 | 130 | 62 | 1039 | 321 | G | 35 | 1.58(1.08;2.32) |  | 2.07(1.28;3.34) | 1.3(0.88;1.91) | 0.02 |
| rs11853833 | 412 | 102 | 756 | 281 | 353 | 136 | 726 | 254 | T | 43 | 1.53(1.18;2) |  | 1.5(1.1;2.04) | 1.43(1.09;1.87) | 0.04 |
| rs12439227 | 263 | 72 | 906 | 311 | 216 | 101 | 863 | 289 | T | 11 | 1.26(0.93;1.71) |  | 1.67(1.16;2.42) | 1.2(0.89;1.64) | 0.03 |
| rs12442926 | 344 | 85 | 825 | 298 | 299 | 117 | 780 | 273 | C | 47 | 1.43(1.08;1.89) |  | 1.56(1.12;2.17) | 1.35(1.01;1.79) | 0.03 |
| rs12899811 | 497 | 155 | 582 | 235 | 505 | 187 | 663 | 196 | G | 34 | 1.4(1.1;1.78) |  | 1.27(0.98;1.64) | 1.01(0.78;1.29) | 0.01 |
| rs12903172 | 439 | 136 | 640 | 254 | 449 | 166 | 719 | 217 | C | 38 | 1.32(1.03;1.69) |  | 1.22(0.93;1.61) | 1.02(0.79;1.32) | 0.03 |
| rs12905881 | 318 | 93 | 761 | 297 | 288 | 120 | 881 | 263 | A | 15 | 1.29(0.98;1.7) |  | 1.43(1.04;1.98) | 1.02(0.77;1.34) | 0.01 |
| rs12910825 | 436 | 132 | 643 | 258 | 429 | 169 | 740 | 214 | G | 39 | 1.38(1.08;1.78) |  | 1.35(1.03;1.78) | 1.01(0.78;1.31) | 0.00 |
| rs12914517 | 446 | 139 | 633 | 251 | 461 | 167 | 707 | 216 | T | 38 | 1.33(1.04;1.7) |  | 1.22(0.93;1.6) | 1.03(0.8;1.33) | 0.03 |
| rs12914720 | 732 | 220 | 437 | 163 | 632 | 242 | 447 | 148 | A | 22 | 1.26(0.99;1.61) |  | 1.26(1.01;1.57) | 1.08(0.84;1.38) | 0.04 |
| rs1532085 | 783 | 241 | 386 | 142 | 669 | 262 | 410 | 128 | A | 40 | 1.23(0.96;1.58) |  | 1.25(1.02;1.55) | 1(0.78;1.29) | 0.03 |
| rs1657922 | 493 | 144 | 676 | 239 | 399 | 159 | 680 | 231 | C | 22 | 1.2(0.94;1.54) |  | 1.38(1.05;1.81) | 1.11(0.87;1.42) | 0.04 |
| rs16976032 | 447 | 134 | 722 | 249 | 404 | 168 | 675 | 222 | G | 22 | 1.17(0.91;1.51) |  | 1.4(1.06;1.84) | 1.08(0.83;1.39) | 0.04 |
| rs16976932 | 989 | 306 | 179 | 77 | 872 | 324 | 207 | 66 | A | 9 | 1.49(1.09;2.02) |  | 1.19(0.98;1.43) | 1.03(0.75;1.41) | 0.03 |
| rs17268471 | 446 | 137 | 633 | 253 | 423 | 150 | 746 | 233 | T | 38 | 1.35(1.05;1.73) |  | 1.2(0.91;1.58) | 1.07(0.83;1.37) | 0.04 |
| rs17527631 | 512 | 144 | 657 | 239 | 415 | 174 | 664 | 216 | T | 36 | 1.24(0.97;1.59) |  | 1.46(1.12;1.9) | 1.09(0.85;1.4) | 0.01 |
| rs17805775 | 296 | 82 | 783 | 308 | 300 | 109 | 869 | 274 | C | 49 | 1.48(1.11;1.98) |  | 1.42(1.01;2) | 1.19(0.89;1.59) | 0.02 |
| rs1820488 | 284 | 80 | 795 | 310 | 289 | 111 | 880 | 272 | G | 14 | 1.4(1.05;1.87) |  | 1.35(0.96;1.9) | 1.15(0.86;1.53) | 0.04 |
| rs1825955 | 372 | 108 | 797 | 275 | 291 | 129 | 788 | 261 | A | 16 | 1.23(0.94;1.6) |  | 1.51(1.1;2.05) | 1.15(0.88;1.5) | 0.03 |
| rs1909884 | 728 | 224 | 441 | 159 | 629 | 247 | 450 | 143 | A | 38 | 1.2(0.94;1.54) |  | 1.26(1.01;1.57) | 1.02(0.8;1.31) | 0.04 |
| rs2043085 | 419 | 128 | 660 | 262 | 384 | 141 | 785 | 242 | T | 40 | 1.31(1.02;1.69) |  | 1.25(0.94;1.67) | 1.03(0.8;1.33) | 0.03 |
| rs2301826 | 459 | 134 | 710 | 249 | 383 | 164 | 696 | 226 | T | 21 | 1.23(0.96;1.58) |  | 1.48(1.12;1.95) | 1.09(0.85;1.41) | 0.02 |
| rs2932206 | 499 | 152 | 670 | 231 | 392 | 165 | 687 | 225 | C | 37 | 1.16(0.91;1.48) |  | 1.39(1.07;1.82) | 1.05(0.82;1.35) | 0.03 |
| rs4779978 | 720 | 224 | 449 | 159 | 624 | 247 | 455 | 143 | T | 38 | 1.17(0.92;1.5) |  | 1.26(1.01;1.57) | 1(0.78;1.29) | 0.04 |
| rs542526 | 544 | 163 | 621 | 220 | 536 | 214 | 539 | 176 | G | 28 | 1.23(0.97;1.57) |  | 1.35(1.05;1.72) | 1.08(0.84;1.4) | 0.03 |
| rs559561 | 362 | 99 | 807 | 284 | 311 | 126 | 768 | 264 | G | 46 | 1.32(1.01;1.73) |  | 1.51(1.1;2.06) | 1.25(0.95;1.64) | 0.04 |
| rs6495126 | 501 | 149 | 578 | 241 | 504 | 172 | 665 | 211 | A | 33 | 1.35(1.06;1.73) |  | 1.16(0.9;1.51) | 1.06(0.82;1.35) | 0.04 |
| rs6495131 | 512 | 150 | 567 | 240 | 512 | 172 | 657 | 211 | A | 33 | 1.39(1.09;1.77) |  | 1.16(0.89;1.5) | 1.08(0.85;1.39) | 0.04 |
| rs690214 | 957 | 292 | 212 | 91 | 869 | 321 | 210 | 69 | T | 44 | 1.5(1.12;2) |  | 1.2(0.99;1.45) | 1.08(0.79;1.49) | 0.03 |
| rs7165170 | 776 | 235 | 393 | 148 | 666 | 255 | 413 | 135 | C | 20 | 1.3(1.01;1.66) |  | 1.28(1.03;1.58) | 1.04(0.81;1.34) | 0.02 |
| rs7166445 | 753 | 219 | 416 | 164 | 674 | 254 | 405 | 136 | G | 21 | 1.32(1.03;1.68) |  | 1.27(1.02;1.57) | 1.1(0.85;1.42) | 0.03 |
| rs7167216 | 981 | 296 | 188 | 87 | 897 | 332 | 182 | 58 | A | 9 | 1.44(1.07;1.94) |  | 1.19(0.98;1.43) | 1.02(0.73;1.42) | 0.03 |
| rs736554 | 692 | 217 | 387 | 173 | 720 | 239 | 449 | 144 | C | 22 | 1.42(1.11;1.81) |  | 1.1(0.88;1.37) | 1.02(0.79;1.31) | 0.03 |
| rs8024276 | 314 | 82 | 855 | 301 | 249 | 109 | 830 | 281 | A | 13 | 1.42(1.06;1.89) |  | 1.72(1.22;2.43) | 1.31(0.98;1.75) | 0.01 |
| rs8026182 | 372 | 98 | 797 | 285 | 348 | 140 | 730 | 250 | C | 43 | 1.49(1.14;1.96) |  | 1.52(1.12;2.08) | 1.4(1.06;1.85) | 0.03 |
| rs8032308 | 282 | 79 | 796 | 311 | 283 | 110 | 886 | 273 | T | 13 | 1.41(1.06;1.88) |  | 1.38(0.98;1.94) | 1.15(0.86;1.54) | 0.03 |
| rs8035159 | 577 | 166 | 592 | 217 | 513 | 204 | 566 | 186 | G | 31 | 1.22(0.96;1.56) |  | 1.34(1.05;1.71) | 1.09(0.85;1.39) | 0.03 |
| rs9972423 | 401 | 121 | 678 | 269 | 421 | 154 | 748 | 229 | A | 40 | 1.33(1.03;1.71) |  | 1.23(0.92;1.63) | 1.06(0.81;1.37) | 0.04 |
| chr 16 |  |  |  |  |  |  |  |  |  |  |  |  |  |  |  |
| rs1122794 | 383 | 115 | 786 | 268 | 353 | 152 | 726 | 238 | A | 18 | 1.15(0.88;1.48) |  | 1.44(1.07;1.92) | 1.06(0.82;1.38) | 0.04 |
| rs11643342 | 548 | 165 | 621 | 218 | 487 | 201 | 592 | 189 | G | 27 | 1.21(0.95;1.54) |  | 1.37(1.06;1.75) | 1.06(0.83;1.36) | 0.02 |
| rs11645023 | 639 | 180 | 530 | 203 | 571 | 214 | 508 | 176 | A | 27 | 1.33(1.05;1.7) |  | 1.32(1.04;1.68) | 1.16(0.9;1.49) | 0.03 |
| rs12446662 | 243 | 73 | 926 | 310 | 228 | 106 | 851 | 284 | T | 12 | 1.13(0.84;1.53) |  | 1.63(1.13;2.33) | 1.08(0.8;1.46) | 0.04 |
| rs12597194 | 582 | 151 | 577 | 227 | 524 | 191 | 549 | 196 | A | 31 | 1.52(1.19;1.94) |  | 1.37(1.07;1.77) | 1.34(1.04;1.72) | 0.03 |
| rs12920377 | 264 | 76 | 905 | 307 | 236 | 107 | 843 | 283 | A | 47 | 1.19(0.89;1.6) |  | 1.66(1.16;2.36) | 1.12(0.83;1.51) | 0.03 |
| rs12934128 | 590 | 155 | 579 | 228 | 529 | 193 | 550 | 197 | T | 30 | 1.5(1.17;1.91) |  | 1.36(1.06;1.75) | 1.33(1.03;1.7) | 0.03 |
| rs13337397 | 250 | 72 | 919 | 311 | 193 | 102 | 886 | 288 | A | 11 | 1.18(0.88;1.6) |  | 1.75(1.21;2.53) | 1.12(0.82;1.52) | 0.02 |
| rs1366754 | 284 | 80 | 795 | 310 | 273 | 100 | 896 | 283 | C | 49 | 1.41(1.06;1.88) |  | 1.35(0.95;1.91) | 1.16(0.87;1.55) | 0.04 |
| rs1423860 | 680 | 193 | 489 | 190 | 614 | 233 | 465 | 157 | G | 24 | 1.34(1.05;1.7) |  | 1.34(1.06;1.68) | 1.1(0.86;1.41) | 0.01 |
| rs149228 | 753 | 219 | 414 | 164 | 702 | 259 | 377 | 131 | C | 20 | 1.44(1.13;1.84) |  | 1.28(1.03;1.58) | 1.18(0.91;1.53) | 0.03 |
| rs1561138 | 777 | 236 | 392 | 147 | 657 | 249 | 422 | 141 | A | 20 | 1.29(1.01;1.65) |  | 1.25(1;1.54) | 1.08(0.84;1.39) | 0.04 |
| rs1609478 | 582 | 175 | 586 | 208 | 535 | 218 | 544 | 172 | C | 30 | 1.31(1.03;1.66) |  | 1.38(1.08;1.75) | 1.1(0.86;1.42) | 0.01 |
| rs16975456 | 198 | 55 | 971 | 328 | 151 | 77 | 928 | 313 | C | 8 | 1.25(0.89;1.75) |  | 1.94(1.28;2.96) | 1.2(0.86;1.68) | 0.02 |
| rs17281013 | 1068 | 328 | 101 | 55 | 974 | 353 | 105 | 37 | A | 5 | 1.77(1.23;2.55) |  | 1.16(0.97;1.39) | 1.09(0.73;1.64) | 0.04 |
| rs17686524 | 1075 | 326 | 94 | 57 | 963 | 347 | 116 | 43 | C | 5 | 1.97(1.37;2.83) |  | 1.17(0.97;1.4) | 1.15(0.78;1.7) | 0.02 |
| rs179771 | 372 | 98 | 797 | 285 | 298 | 124 | 781 | 266 | C | 46 | 1.29(0.98;1.68) |  | 1.5(1.09;2.06) | 1.22(0.93;1.59) | 0.04 |
| rs1861352 | 682 | 201 | 487 | 182 | 562 | 219 | 517 | 171 | A | 26 | 1.23(0.97;1.57) |  | 1.31(1.04;1.65) | 1.06(0.83;1.35) | 0.03 |
| rs1875236 | 239 | 62 | 930 | 321 | 172 | 85 | 907 | 305 | A | 10 | 1.33(0.97;1.82) |  | 1.86(1.26;2.76) | 1.26(0.92;1.73) | 0.02 |
| rs193773 | 261 | 75 | 908 | 308 | 205 | 100 | 874 | 290 | C | 11 | 1.3(0.96;1.76) |  | 1.77(1.23;2.55) | 1.22(0.9;1.65) | 0.02 |
| rs2003400 | 551 | 162 | 618 | 221 | 475 | 197 | 604 | 193 | A | 26 | 1.26(0.99;1.6) |  | 1.4(1.09;1.8) | 1.09(0.85;1.4) | 0.02 |
| rs235802 | 447 | 111 | 722 | 272 | 391 | 152 | 688 | 238 | A | 40 | 1.52(1.17;1.97) |  | 1.57(1.17;2.1) | 1.34(1.03;1.75) | 0.01 |
| rs28769 | 261 | 77 | 908 | 306 | 206 | 103 | 873 | 287 | C | 11 | 1.26(0.93;1.69) |  | 1.77(1.23;2.54) | 1.18(0.87;1.59) | 0.02 |
| rs2966245 | 398 | 114 | 771 | 269 | 351 | 151 | 728 | 239 | G | 42 | 1.26(0.97;1.63) |  | 1.48(1.11;1.99) | 1.15(0.88;1.5) | 0.02 |
| rs37038 | 988 | 304 | 181 | 79 | 905 | 333 | 174 | 57 | G | 9 | 1.54(1.13;2.09) |  | 1.19(0.99;1.44) | 1.03(0.74;1.45) | 0.02 |
| rs3736338 | 243 | 63 | 926 | 320 | 211 | 88 | 868 | 301 | G | 45 | 1.5(1.09;2.07) |  | 1.77(1.2;2.6) | 1.43(1.04;1.97) | 0.03 |
| rs3743614 | 249 | 70 | 920 | 313 | 192 | 102 | 887 | 288 | T | 11 | 1.23(0.9;1.66) |  | 1.82(1.25;2.63) | 1.15(0.84;1.56) | 0.02 |
| rs3794689 | 965 | 317 | 114 | 73 | 994 | 323 | 175 | 60 | A | 7 | 1.97(1.41;2.74) |  | 1.02(0.84;1.23) | 1.03(0.74;1.44) | 0.01 |
| rs3923425 | 372 | 106 | 797 | 277 | 300 | 130 | 779 | 260 | A | 16 | 1.24(0.95;1.62) |  | 1.52(1.11;2.07) | 1.16(0.89;1.51) | 0.03 |
| rs40448 | 260 | 74 | 909 | 309 | 205 | 102 | 874 | 288 | C | 11 | 1.31(0.97;1.78) |  | 1.82(1.27;2.63) | 1.22(0.9;1.66) | 0.01 |
| rs411103 | 405 | 103 | 763 | 280 | 332 | 138 | 747 | 252 | A | 43 | 1.47(1.12;1.91) |  | 1.65(1.22;2.24) | 1.3(0.99;1.7) | 0.01 |
| rs4395067 | 722 | 207 | 447 | 176 | 636 | 240 | 443 | 150 | A | 23 | 1.32(1.04;1.69) |  | 1.27(1.02;1.59) | 1.14(0.88;1.46) | 0.04 |
| rs4548853 | 205 | 48 | 964 | 335 | 158 | 61 | 921 | 329 | G | 39 | 1.58(1.11;2.25) |  | 1.95(1.24;3.05) | 1.54(1.08;2.2) | 0.04 |
| rs4843199 | 570 | 170 | 599 | 213 | 503 | 209 | 574 | 181 | C | 28 | 1.24(0.98;1.58) |  | 1.4(1.09;1.78) | 1.06(0.82;1.36) | 0.01 |
| rs6564722 | 631 | 179 | 538 | 204 | 537 | 211 | 542 | 179 | T | 28 | 1.3(1.02;1.65) |  | 1.36(1.07;1.72) | 1.11(0.87;1.42) | 0.02 |
| rs6564724 | 556 | 161 | 613 | 222 | 472 | 186 | 607 | 204 | G | 32 | 1.23(0.97;1.57) |  | 1.34(1.04;1.72) | 1.12(0.87;1.43) | 0.04 |
| rs7188674 | 617 | 175 | 552 | 208 | 546 | 206 | 533 | 184 | T | 28 | 1.32(1.04;1.68) |  | 1.33(1.04;1.69) | 1.16(0.91;1.49) | 0.03 |
| rs7189970 | 458 | 130 | 621 | 260 | 459 | 152 | 710 | 231 | G | 37 | 1.48(1.15;1.9) |  | 1.22(0.93;1.62) | 1.16(0.9;1.49) | 0.03 |
| rs7193869 | 618 | 174 | 551 | 209 | 524 | 208 | 555 | 182 | T | 29 | 1.3(1.02;1.65) |  | 1.32(1.04;1.68) | 1.15(0.9;1.47) | 0.04 |
| rs7202877 | 250 | 69 | 919 | 314 | 192 | 101 | 887 | 289 | G | 11 | 1.25(0.92;1.7) |  | 1.83(1.27;2.66) | 1.17(0.86;1.6) | 0.01 |
| rs7206275 | 591 | 155 | 578 | 228 | 529 | 193 | 550 | 197 | T | 30 | 1.5(1.18;1.91) |  | 1.37(1.06;1.76) | 1.33(1.04;1.71) | 0.03 |
| rs767018 | 882 | 255 | 287 | 128 | 816 | 297 | 263 | 93 | T | 14 | 1.62(1.25;2.1) |  | 1.25(1.02;1.53) | 1.21(0.92;1.61) | 0.02 |
| rs8043572 | 1069 | 328 | 100 | 55 | 973 | 353 | 106 | 37 | G | 5 | 1.8(1.24;2.59) |  | 1.16(0.97;1.39) | 1.09(0.72;1.63) | 0.03 |
| rs8056814 | 200 | 52 | 969 | 331 | 146 | 77 | 933 | 313 | A | 8 | 1.35(0.96;1.91) |  | 2.05(1.34;3.13) | 1.29(0.92;1.82) | 0.02 |
| rs959733 | 812 | 231 | 357 | 152 | 748 | 268 | 331 | 122 | G | 17 | 1.55(1.21;1.99) |  | 1.25(1.01;1.54) | 1.28(0.99;1.67) | 0.04 |
| rs9926666 | 275 | 80 | 894 | 303 | 246 | 108 | 832 | 282 | A | 48 | 1.18(0.88;1.57) |  | 1.59(1.12;2.25) | 1.12(0.84;1.5) | 0.04 |
| rs9927309 | 304 | 83 | 865 | 300 | 253 | 120 | 826 | 270 | T | 13 | 1.27(0.96;1.69) |  | 1.7(1.22;2.39) | 1.17(0.88;1.56) | 0.01 |
| rs9928833 | 403 | 121 | 766 | 262 | 327 | 143 | 752 | 247 | C | 18 | 1.15(0.89;1.49) |  | 1.46(1.09;1.96) | 1.07(0.83;1.39) | 0.03 |
| chr 17 |  |  |  |  |  |  |  |  |  |  |  |  |  |  |  |
| rs11079016 | 813 | 242 | 356 | 141 | 743 | 277 | 336 | 112 | A | 17 | 1.38(1.08;1.78) |  | 1.27(1.03;1.57) | 1.05(0.81;1.38) | 0.01 |
| rs11657055 | 346 | 101 | 733 | 289 | 338 | 120 | 831 | 263 | A | 46 | 1.37(1.05;1.79) |  | 1.3(0.95;1.78) | 1.1(0.84;1.45) | 0.03 |
| rs11657214 | 275 | 58 | 892 | 325 | 206 | 79 | 871 | 310 | G | 45 | 1.68(1.21;2.32) |  | 1.81(1.21;2.69) | 1.6(1.16;2.22) | 0.03 |
| rs12449740 | 297 | 93 | 781 | 297 | 333 | 129 | 836 | 254 | A | 47 | 1.28(0.97;1.69) |  | 1.28(0.93;1.77) | 1.04(0.78;1.38) | 0.04 |
| rs12600461 | 381 | 117 | 788 | 266 | 331 | 144 | 748 | 246 | C | 18 | 1.25(0.96;1.62) |  | 1.53(1.13;2.05) | 1.14(0.87;1.48) | 0.02 |
| rs12945695 | 213 | 64 | 956 | 319 | 160 | 81 | 919 | 309 | C | 9 | 1.15(0.84;1.59) |  | 1.76(1.17;2.63) | 1.12(0.81;1.55) | 0.04 |
| rs13412 | 467 | 135 | 702 | 248 | 424 | 169 | 655 | 221 | C | 37 | 1.26(0.98;1.62) |  | 1.43(1.09;1.88) | 1.13(0.88;1.47) | 0.02 |
| rs16960103 | 775 | 243 | 304 | 147 | 809 | 264 | 360 | 119 | A | 17 | 1.5(1.17;1.93) |  | 1.07(0.87;1.31) | 1.05(0.8;1.36) | 0.03 |
| rs17690038 | 641 | 179 | 527 | 204 | 622 | 234 | 457 | 156 | G | 25 | 1.4(1.1;1.78) |  | 1.34(1.06;1.69) | 1.19(0.92;1.53) | 0.02 |
| rs17759555 | 104 | 22 | 975 | 368 | 72 | 37 | 1097 | 346 | C | 28 | 1.87(1.15;3.03) |  | 2.59(1.38;4.83) | 1.59(0.97;2.58) | 0.04 |
| rs1962289 | 376 | 120 | 761 | 249 | 349 | 150 | 708 | 226 | G | 19 | 1.11(0.86;1.44) |  | 1.42(1.06;1.9) | 1.03(0.79;1.34) | 0.04 |
| rs2280785 | 350 | 108 | 819 | 275 | 280 | 127 | 799 | 263 | T | 16 | 1.15(0.88;1.5) |  | 1.5(1.1;2.05) | 1.08(0.83;1.42) | 0.04 |
| rs231492 | 191 | 54 | 888 | 336 | 188 | 79 | 981 | 304 | T | 9 | 1.38(0.98;1.93) |  | 1.65(1.09;2.5) | 1.13(0.81;1.59) | 0.02 |
| rs2338800 | 479 | 142 | 690 | 241 | 443 | 181 | 636 | 209 | C | 24 | 1.2(0.94;1.54) |  | 1.37(1.05;1.79) | 1.09(0.85;1.41) | 0.04 |
| rs2378908 | 381 | 120 | 788 | 263 | 333 | 143 | 746 | 247 | A | 18 | 1.14(0.88;1.48) |  | 1.43(1.07;1.93) | 1.07(0.82;1.39) | 0.04 |
| rs2716194 | 1059 | 322 | 110 | 61 | 950 | 344 | 129 | 46 | C | 6 | 2(1.41;2.83) |  | 1.16(0.97;1.4) | 1.26(0.87;1.83) | 0.03 |
| rs3096644 | 673 | 209 | 496 | 174 | 599 | 238 | 480 | 152 | T | 35 | 1.19(0.93;1.51) |  | 1.29(1.03;1.62) | 1.01(0.79;1.3) | 0.03 |
| rs3110496 | 156 | 40 | 923 | 350 | 143 | 61 | 1026 | 322 | G | 37 | 1.45(0.99;2.11) |  | 1.73(1.08;2.78) | 1.22(0.83;1.77) | 0.04 |
| rs3744549 | 350 | 103 | 729 | 287 | 352 | 133 | 817 | 250 | C | 17 | 1.27(0.97;1.65) |  | 1.27(0.93;1.72) | 1.02(0.78;1.34) | 0.04 |
| rs383123 | 250 | 75 | 829 | 315 | 245 | 89 | 924 | 294 | T | 48 | 1.33(0.99;1.79) |  | 1.39(0.96;2.01) | 1.11(0.82;1.49) | 0.04 |
| rs4239020 | 544 | 165 | 535 | 225 | 585 | 200 | 584 | 183 | C | 30 | 1.36(1.07;1.72) |  | 1.13(0.89;1.45) | 1.05(0.82;1.34) | 0.04 |
| rs4416064 | 269 | 69 | 810 | 321 | 263 | 93 | 906 | 290 | C | 47 | 1.61(1.19;2.18) |  | 1.44(1;2.08) | 1.32(0.97;1.79) | 0.03 |
| rs4422036 | 425 | 135 | 744 | 248 | 369 | 167 | 710 | 223 | A | 20 | 1.13(0.88;1.46) |  | 1.49(1.13;1.97) | 1(0.78;1.3) | 0.01 |
| rs4522464 | 380 | 116 | 789 | 267 | 331 | 143 | 748 | 247 | G | 18 | 1.26(0.97;1.63) |  | 1.52(1.13;2.05) | 1.15(0.88;1.49) | 0.02 |
| rs4790104 | 484 | 143 | 595 | 247 | 504 | 175 | 664 | 208 | G | 35 | 1.44(1.12;1.84) |  | 1.19(0.92;1.55) | 1.11(0.86;1.43) | 0.03 |
| rs4792814 | 400 | 116 | 679 | 274 | 428 | 149 | 741 | 234 | T | 40 | 1.44(1.11;1.86) |  | 1.25(0.93;1.66) | 1.15(0.88;1.49) | 0.03 |
| rs4795101 | 331 | 94 | 838 | 289 | 256 | 118 | 823 | 272 | G | 14 | 1.31(0.99;1.73) |  | 1.72(1.23;2.39) | 1.19(0.9;1.58) | 0.01 |
| rs4925159 | 198 | 48 | 881 | 342 | 162 | 62 | 1007 | 321 | G | 39 | 1.62(1.14;2.29) |  | 1.61(1.04;2.51) | 1.36(0.96;1.92) | 0.04 |
| rs4968455 | 437 | 134 | 732 | 249 | 379 | 169 | 700 | 221 | C | 21 | 1.2(0.93;1.54) |  | 1.53(1.16;2.02) | 1.04(0.81;1.35) | 0.01 |
| rs4968456 | 423 | 129 | 704 | 232 | 365 | 160 | 683 | 213 | C | 21 | 1.14(0.88;1.47) |  | 1.51(1.14;2) | 1.02(0.78;1.32) | 0.01 |
| rs4969260 | 376 | 97 | 793 | 286 | 335 | 128 | 744 | 262 | G | 45 | 1.36(1.04;1.78) |  | 1.51(1.1;2.06) | 1.27(0.97;1.67) | 0.04 |
| rs6504084 | 702 | 224 | 377 | 166 | 755 | 245 | 414 | 138 | A | 20 | 1.42(1.11;1.82) |  | 1.08(0.87;1.34) | 1.04(0.8;1.34) | 0.04 |
| rs686994 | 336 | 103 | 743 | 287 | 361 | 134 | 808 | 249 | C | 45 | 1.32(1.01;1.73) |  | 1.32(0.97;1.79) | 1.05(0.8;1.38) | 0.02 |
| rs7211137 | 528 | 158 | 641 | 225 | 452 | 187 | 627 | 203 | C | 25 | 1.18(0.92;1.5) |  | 1.37(1.06;1.77) | 1.05(0.82;1.35) | 0.03 |
| rs7220650 | 481 | 142 | 688 | 241 | 447 | 181 | 632 | 209 | C | 24 | 1.21(0.95;1.55) |  | 1.36(1.05;1.77) | 1.11(0.86;1.43) | 0.04 |
| rs741071 | 281 | 72 | 884 | 310 | 224 | 97 | 854 | 293 | C | 47 | 1.37(1.02;1.85) |  | 1.69(1.17;2.44) | 1.31(0.97;1.77) | 0.03 |
| rs741072 | 272 | 69 | 896 | 313 | 219 | 94 | 859 | 296 | C | 46 | 1.4(1.03;1.9) |  | 1.69(1.17;2.46) | 1.35(0.99;1.83) | 0.03 |
| rs8065435 | 671 | 205 | 497 | 178 | 587 | 230 | 492 | 160 | A | 34 | 1.22(0.96;1.55) |  | 1.28(1.02;1.61) | 1.06(0.83;1.35) | 0.04 |
| rs8069937 | 274 | 69 | 804 | 321 | 239 | 90 | 930 | 293 | G | 47 | 1.51(1.12;2.04) |  | 1.39(0.96;2.01) | 1.25(0.92;1.69) | 0.04 |
| rs8081812 | 578 | 166 | 501 | 224 | 648 | 210 | 521 | 173 | T | 27 | 1.62(1.27;2.06) |  | 1.22(0.96;1.55) | 1.16(0.9;1.5) | 0.01 |
| rs86312 | 177 | 47 | 902 | 343 | 159 | 79 | 1008 | 304 | C | 8 | 1.51(1.06;2.16) |  | 1.87(1.21;2.9) | 1.23(0.86;1.76) | 0.01 |
| rs890435 | 745 | 232 | 423 | 151 | 668 | 262 | 411 | 127 | C | 40 | 1.22(0.95;1.56) |  | 1.25(1.01;1.55) | 1.01(0.79;1.31) | 0.04 |
| rs9889324 | 572 | 162 | 507 | 228 | 623 | 210 | 546 | 173 | A | 28 | 1.65(1.3;2.1) |  | 1.29(1.01;1.64) | 1.12(0.87;1.44) | 0.00 |
| rs9890911 | 340 | 97 | 829 | 286 | 313 | 140 | 766 | 250 | T | 45 | 1.21(0.92;1.59) |  | 1.48(1.08;2.03) | 1.14(0.86;1.5) | 0.04 |
| rs9895985 | 619 | 194 | 460 | 196 | 624 | 213 | 545 | 170 | T | 26 | 1.37(1.08;1.75) |  | 1.1(0.87;1.4) | 1.04(0.81;1.33) | 0.04 |
| rs9907899 | 524 | 157 | 639 | 223 | 450 | 184 | 624 | 203 | T | 25 | 1.17(0.92;1.49) |  | 1.35(1.04;1.74) | 1.06(0.82;1.35) | 0.04 |
| rs999493 | 715 | 222 | 454 | 161 | 642 | 252 | 437 | 138 | G | 37 | 1.19(0.93;1.52) |  | 1.26(1.01;1.57) | 1.01(0.78;1.3) | 0.04 |
| chr 18 |  |  |  |  |  |  |  |  |  |  |  |  |  |  |  |
| rs1027676 | 431 | 121 | 738 | 262 | 411 | 167 | 668 | 223 | G | 21 | 1.31(1.01;1.69) |  | 1.43(1.08;1.9) | 1.2(0.93;1.57) | 0.04 |
| rs11151841 | 352 | 99 | 817 | 284 | 336 | 144 | 743 | 246 | G | 17 | 1.25(0.96;1.64) |  | 1.49(1.09;2.03) | 1.17(0.89;1.55) | 0.04 |
| rs11659955 | 199 | 62 | 880 | 328 | 186 | 84 | 983 | 299 | C | 9 | 1.24(0.9;1.71) |  | 1.51(1.01;2.25) | 1.03(0.75;1.43) | 0.04 |
| rs12709693 | 545 | 150 | 624 | 233 | 463 | 179 | 616 | 211 | C | 34 | 1.41(1.11;1.8) |  | 1.38(1.06;1.79) | 1.26(0.99;1.62) | 0.03 |
| rs12959219 | 599 | 169 | 570 | 214 | 544 | 206 | 535 | 184 | G | 29 | 1.32(1.04;1.68) |  | 1.33(1.04;1.69) | 1.17(0.91;1.5) | 0.04 |
| rs1364419 | 867 | 257 | 302 | 126 | 775 | 288 | 304 | 102 | C | 15 | 1.35(1.04;1.76) |  | 1.23(1;1.5) | 1.07(0.81;1.4) | 0.03 |
| rs1472671 | 363 | 102 | 806 | 281 | 348 | 147 | 731 | 243 | T | 17 | 1.28(0.98;1.68) |  | 1.48(1.09;2.01) | 1.2(0.91;1.58) | 0.04 |
| rs17078836 | 164 | 38 | 1005 | 345 | 132 | 65 | 947 | 325 | A | 7 | 1.4(0.95;2.06) |  | 2.13(1.32;3.43) | 1.36(0.92;2) | 0.03 |
| rs17187936 | 995 | 303 | 174 | 80 | 896 | 328 | 183 | 62 | T | 9 | 1.5(1.1;2.03) |  | 1.18(0.98;1.43) | 1.05(0.75;1.45) | 0.03 |
| rs17187964 | 995 | 303 | 174 | 80 | 896 | 328 | 183 | 62 | A | 9 | 1.5(1.1;2.03) |  | 1.18(0.98;1.43) | 1.05(0.75;1.45) | 0.03 |
| rs17569530 | 697 | 192 | 472 | 191 | 616 | 234 | 463 | 156 | A | 24 | 1.5(1.18;1.91) |  | 1.4(1.11;1.76) | 1.17(0.91;1.5) | 0.00 |
| rs17656498 | 492 | 154 | 587 | 236 | 486 | 171 | 683 | 212 | C | 35 | 1.31(1.03;1.67) |  | 1.17(0.9;1.52) | 1.02(0.79;1.3) | 0.04 |
| rs1788025 | 201 | 55 | 878 | 335 | 181 | 75 | 988 | 308 | A | 41 | 1.38(0.99;1.93) |  | 1.54(1.02;2.34) | 1.15(0.83;1.61) | 0.04 |
| rs1788583 | 603 | 190 | 476 | 200 | 576 | 202 | 593 | 181 | A | 28 | 1.35(1.06;1.72) |  | 1.13(0.89;1.43) | 1.01(0.79;1.29) | 0.03 |
| rs1788823 | 499 | 132 | 669 | 251 | 401 | 158 | 678 | 232 | A | 37 | 1.42(1.11;1.82) |  | 1.45(1.1;1.91) | 1.27(0.99;1.64) | 0.02 |
| rs1790947 | 297 | 75 | 872 | 308 | 256 | 104 | 823 | 286 | G | 49 | 1.42(1.06;1.91) |  | 1.62(1.14;2.3) | 1.36(1.01;1.82) | 0.04 |
| rs2008401 | 286 | 90 | 787 | 297 | 258 | 103 | 906 | 279 | T | 13 | 1.27(0.96;1.68) |  | 1.34(0.96;1.89) | 1.05(0.79;1.4) | 0.04 |
| rs2113744 | 1055 | 343 | 114 | 40 | 980 | 333 | 99 | 57 | G | 5 | 1.01(0.67;1.51) |  | 1.01(0.84;1.21) | 1.85(1.29;2.65) | 0.03 |
| rs2178641 | 324 | 88 | 845 | 295 | 267 | 113 | 812 | 277 | A | 49 | 1.28(0.97;1.69) |  | 1.56(1.12;2.18) | 1.21(0.92;1.61) | 0.03 |
| rs2385660 | 544 | 149 | 623 | 231 | 462 | 179 | 614 | 210 | G | 34 | 1.4(1.1;1.79) |  | 1.39(1.07;1.8) | 1.27(0.99;1.63) | 0.03 |
| rs242512 | 654 | 195 | 515 | 188 | 595 | 233 | 484 | 157 | C | 26 | 1.29(1.02;1.65) |  | 1.31(1.04;1.65) | 1.1(0.86;1.41) | 0.03 |
| rs2846642 | 205 | 49 | 964 | 334 | 171 | 78 | 908 | 312 | C | 40 | 1.44(1.02;2.04) |  | 1.88(1.23;2.87) | 1.4(0.99;1.98) | 0.03 |
| rs3744905 | 1071 | 325 | 98 | 58 | 957 | 348 | 122 | 42 | A | 5 | 1.88(1.31;2.69) |  | 1.17(0.97;1.4) | 1.1(0.75;1.62) | 0.02 |
| rs4374255 | 436 | 140 | 643 | 250 | 415 | 150 | 753 | 233 | G | 39 | 1.31(1.03;1.69) |  | 1.22(0.92;1.61) | 1.03(0.8;1.32) | 0.03 |
| rs4939880 | 331 | 95 | 748 | 295 | 335 | 114 | 834 | 269 | C | 46 | 1.42(1.08;1.87) |  | 1.29(0.93;1.78) | 1.16(0.88;1.53) | 0.04 |
| rs6505545 | 555 | 171 | 614 | 212 | 526 | 212 | 553 | 178 | G | 28 | 1.17(0.92;1.49) |  | 1.35(1.06;1.72) | 1.02(0.79;1.3) | 0.02 |
| rs6507823 | 719 | 210 | 450 | 173 | 640 | 240 | 439 | 150 | A | 22 | 1.38(1.08;1.76) |  | 1.28(1.02;1.6) | 1.17(0.91;1.51) | 0.04 |
| rs7227855 | 633 | 194 | 446 | 196 | 661 | 218 | 507 | 165 | T | 25 | 1.46(1.15;1.86) |  | 1.14(0.9;1.43) | 1.06(0.83;1.36) | 0.02 |
| rs7233494 | 895 | 268 | 274 | 115 | 814 | 306 | 265 | 84 | T | 50 | 1.44(1.11;1.89) |  | 1.23(1.01;1.5) | 1.07(0.8;1.43) | 0.02 |
| rs7235005 | 330 | 95 | 749 | 295 | 325 | 115 | 844 | 268 | G | 46 | 1.38(1.05;1.82) |  | 1.29(0.93;1.79) | 1.13(0.85;1.49) | 0.04 |
| rs7235689 | 902 | 260 | 267 | 123 | 777 | 291 | 302 | 99 | C | 14 | 1.62(1.24;2.1) |  | 1.3(1.07;1.59) | 1.05(0.79;1.38) | 0.00 |
| rs7236409 | 202 | 51 | 967 | 332 | 165 | 76 | 914 | 314 | G | 41 | 1.38(0.98;1.95) |  | 1.83(1.2;2.8) | 1.34(0.95;1.9) | 0.04 |
| rs7237871 | 633 | 194 | 446 | 196 | 663 | 218 | 506 | 165 | A | 25 | 1.46(1.15;1.86) |  | 1.13(0.9;1.42) | 1.06(0.83;1.36) | 0.02 |
| rs7243350 | 848 | 267 | 231 | 123 | 888 | 287 | 281 | 96 | T | 13 | 1.67(1.28;2.18) |  | 1.03(0.85;1.26) | 1.15(0.87;1.52) | 0.04 |
| rs8085585 | 305 | 85 | 864 | 298 | 254 | 114 | 825 | 276 | T | 14 | 1.23(0.93;1.63) |  | 1.56(1.11;2.19) | 1.17(0.88;1.55) | 0.04 |
| rs8092503 | 662 | 199 | 507 | 184 | 618 | 237 | 461 | 153 | G | 25 | 1.21(0.95;1.54) |  | 1.28(1.02;1.61) | 1.04(0.81;1.34) | 0.04 |
| rs878468 | 545 | 150 | 624 | 233 | 463 | 179 | 616 | 211 | C | 34 | 1.41(1.11;1.8) |  | 1.38(1.06;1.79) | 1.26(0.99;1.62) | 0.03 |
| rs9635953 | 1046 | 320 | 123 | 63 | 939 | 347 | 140 | 43 | G | 6 | 1.63(1.16;2.31) |  | 1.17(0.97;1.4) | 1.05(0.72;1.53) | 0.03 |
| rs9946982 | 660 | 198 | 509 | 185 | 612 | 242 | 467 | 148 | C | 35 | 1.19(0.94;1.52) |  | 1.28(1.02;1.61) | 1.03(0.8;1.32) | 0.04 |
| rs9955814 | 777 | 224 | 392 | 159 | 694 | 256 | 385 | 134 | G | 20 | 1.35(1.06;1.73) |  | 1.25(1.01;1.55) | 1.14(0.88;1.47) | 0.04 |
| rs9964423 | 569 | 158 | 600 | 225 | 525 | 201 | 554 | 189 | C | 31 | 1.38(1.08;1.76) |  | 1.39(1.08;1.78) | 1.2(0.93;1.54) | 0.02 |
| chr19 |  |  |  |  |  |  |  |  |  |  |  |  |  |  |  |
| rs1035458 | 521 | 153 | 648 | 230 | 487 | 193 | 592 | 197 | C | 33 | 1.2(0.94;1.54) |  | 1.34(1.04;1.74) | 1.09(0.85;1.4) | 0.04 |
| rs10408844 | 391 | 110 | 778 | 273 | 363 | 152 | 716 | 238 | C | 19 | 1.27(0.98;1.66) |  | 1.46(1.09;1.96) | 1.18(0.9;1.54) | 0.03 |
| rs11085852 | 455 | 129 | 712 | 254 | 413 | 162 | 665 | 226 | T | 39 | 1.32(1.02;1.69) |  | 1.42(1.07;1.87) | 1.19(0.92;1.54) | 0.03 |
| rs11672743 | 749 | 213 | 415 | 170 | 686 | 263 | 393 | 127 | C | 21 | 1.42(1.11;1.81) |  | 1.35(1.08;1.67) | 1.06(0.82;1.38) | 0.00 |
| rs12985909 | 327 | 96 | 752 | 294 | 352 | 139 | 817 | 244 | C | 45 | 1.41(1.07;1.85) |  | 1.41(1.04;1.93) | 1.09(0.82;1.44) | 0.01 |
| rs1688005 | 638 | 193 | 530 | 190 | 555 | 221 | 524 | 169 | G | 27 | 1.18(0.93;1.5) |  | 1.29(1.02;1.63) | 1.04(0.81;1.32) | 0.04 |
| rs16964420 | 528 | 146 | 641 | 237 | 427 | 170 | 650 | 219 | A | 35 | 1.38(1.08;1.77) |  | 1.45(1.11;1.89) | 1.21(0.94;1.55) | 0.01 |
| rs17718517 | 501 | 145 | 667 | 238 | 444 | 184 | 633 | 206 | C | 24 | 1.24(0.97;1.59) |  | 1.43(1.1;1.86) | 1.1(0.85;1.41) | 0.02 |
| rs2015728 | 217 | 60 | 950 | 323 | 173 | 83 | 906 | 307 | T | 9 | 1.23(0.89;1.7) |  | 1.83(1.22;2.73) | 1.18(0.85;1.64) | 0.03 |
| rs2232965 | 516 | 157 | 653 | 226 | 457 | 192 | 622 | 198 | T | 25 | 1.14(0.89;1.45) |  | 1.36(1.06;1.76) | 1.02(0.79;1.3) | 0.03 |
| rs2288419 | 440 | 127 | 729 | 256 | 390 | 174 | 689 | 216 | C | 21 | 1.18(0.92;1.52) |  | 1.51(1.15;1.99) | 1.03(0.79;1.33) | 0.01 |
| rs2370134 | 842 | 246 | 327 | 137 | 762 | 285 | 317 | 105 | T | 16 | 1.49(1.15;1.93) |  | 1.26(1.03;1.55) | 1.13(0.86;1.49) | 0.01 |
| rs2523177 | 758 | 225 | 411 | 158 | 699 | 265 | 380 | 125 | T | 20 | 1.34(1.05;1.71) |  | 1.27(1.03;1.58) | 1.08(0.83;1.4) | 0.02 |
| rs308200 | 508 | 153 | 571 | 237 | 544 | 190 | 625 | 192 | C | 31 | 1.4(1.1;1.79) |  | 1.2(0.93;1.55) | 1.05(0.82;1.35) | 0.02 |
| rs4239635 | 588 | 182 | 491 | 208 | 605 | 207 | 563 | 176 | G | 28 | 1.41(1.11;1.79) |  | 1.13(0.89;1.43) | 1.06(0.83;1.36) | 0.03 |
| rs4803880 | 302 | 75 | 866 | 307 | 247 | 98 | 832 | 292 | T | 49 | 1.44(1.07;1.95) |  | 1.66(1.16;2.38) | 1.38(1.02;1.86) | 0.03 |
| rs4805909 | 305 | 92 | 774 | 297 | 295 | 114 | 874 | 269 | A | 14 | 1.32(1;1.75) |  | 1.36(0.98;1.88) | 1.07(0.81;1.41) | 0.03 |
| rs7258489 | 588 | 170 | 581 | 213 | 511 | 201 | 568 | 189 | T | 30 | 1.24(0.97;1.57) |  | 1.32(1.03;1.69) | 1.11(0.87;1.42) | 0.04 |
| rs8102478 | 607 | 162 | 538 | 217 | 497 | 201 | 565 | 183 | T | 30 | 1.48(1.16;1.89) |  | 1.49(1.16;1.9) | 1.16(0.9;1.49) | 0.00 |
| rs8103534 | 587 | 171 | 582 | 212 | 512 | 204 | 567 | 186 | C | 30 | 1.22(0.96;1.55) |  | 1.33(1.04;1.69) | 1.09(0.85;1.39) | 0.04 |
| rs8103733 | 275 | 83 | 893 | 300 | 239 | 104 | 840 | 286 | T | 12 | 1.22(0.91;1.63) |  | 1.58(1.11;2.25) | 1.17(0.87;1.57) | 0.04 |
| rs890862 | 548 | 169 | 531 | 221 | 586 | 200 | 583 | 183 | T | 30 | 1.31(1.03;1.67) |  | 1.13(0.88;1.44) | 1.01(0.79;1.3) | 0.04 |
| chr 20 |  |  |  |  |  |  |  |  |  |  |  |  |  |  |  |
| rs1005987 | 765 | 218 | 404 | 165 | 693 | 263 | 386 | 127 | T | 20 | 1.34(1.05;1.71) |  | 1.28(1.03;1.58) | 1.09(0.84;1.41) | 0.02 |
| rs1028540 | 236 | 56 | 933 | 327 | 173 | 76 | 906 | 314 | C | 43 | 1.5(1.08;2.09) |  | 1.92(1.27;2.91) | 1.43(1.03;2) | 0.02 |
| rs11087739 | 444 | 133 | 725 | 250 | 390 | 157 | 689 | 233 | G | 39 | 1.24(0.96;1.59) |  | 1.41(1.07;1.87) | 1.14(0.88;1.47) | 0.04 |
| rs11696118 | 437 | 131 | 732 | 252 | 392 | 156 | 687 | 234 | C | 39 | 1.24(0.96;1.6) |  | 1.41(1.06;1.86) | 1.15(0.89;1.49) | 0.04 |
| rs13036656 | 298 | 77 | 871 | 306 | 231 | 102 | 848 | 288 | A | 48 | 1.35(1.01;1.81) |  | 1.68(1.18;2.39) | 1.27(0.95;1.7) | 0.02 |
| rs1406966 | 817 | 235 | 352 | 148 | 728 | 274 | 351 | 116 | A | 18 | 1.47(1.14;1.88) |  | 1.3(1.06;1.6) | 1.08(0.83;1.41) | 0.01 |
| rs156353 | 438 | 123 | 731 | 260 | 333 | 136 | 746 | 254 | T | 43 | 1.31(1.02;1.69) |  | 1.46(1.09;1.96) | 1.22(0.94;1.57) | 0.03 |
| rs1780706 | 815 | 225 | 354 | 158 | 742 | 263 | 337 | 127 | C | 18 | 1.61(1.26;2.07) |  | 1.3(1.05;1.61) | 1.23(0.95;1.6) | 0.01 |
| rs1891582 | 125 | 31 | 954 | 359 | 128 | 60 | 1041 | 323 | A | 6 | 1.72(1.12;2.63) |  | 2.01(1.2;3.37) | 1.44(0.94;2.22) | 0.03 |
| rs1935671 | 659 | 186 | 510 | 197 | 586 | 224 | 493 | 165 | C | 26 | 1.33(1.05;1.69) |  | 1.31(1.03;1.65) | 1.14(0.89;1.47) | 0.03 |
| rs2185558 | 794 | 253 | 285 | 137 | 838 | 264 | 331 | 119 | C | 16 | 1.63(1.26;2.11) |  | 1.06(0.86;1.3) | 1.12(0.86;1.45) | 0.02 |
| rs2236164 | 518 | 159 | 651 | 224 | 479 | 194 | 600 | 196 | C | 26 | 1.15(0.9;1.47) |  | 1.34(1.04;1.73) | 1.04(0.81;1.33) | 0.04 |
| rs224371 | 520 | 160 | 649 | 223 | 481 | 196 | 598 | 194 | G | 26 | 1.15(0.9;1.46) |  | 1.35(1.05;1.73) | 1.03(0.8;1.32) | 0.04 |
| rs224436 | 630 | 198 | 449 | 192 | 693 | 234 | 476 | 149 | C | 24 | 1.4(1.1;1.78) |  | 1.1(0.88;1.38) | 1.05(0.81;1.35) | 0.04 |
| rs2277862 | 793 | 257 | 286 | 133 | 873 | 291 | 296 | 92 | T | 14 | 1.52(1.17;1.97) |  | 1.06(0.86;1.29) | 1.03(0.78;1.37) | 0.03 |
| rs228835 | 192 | 41 | 977 | 342 | 138 | 58 | 941 | 332 | T | 36 | 1.76(1.21;2.57) |  | 2.13(1.32;3.42) | 1.71(1.17;2.5) | 0.03 |
| rs228836 | 198 | 42 | 971 | 341 | 139 | 58 | 940 | 332 | A | 37 | 1.78(1.22;2.58) |  | 2.11(1.32;3.38) | 1.72(1.18;2.51) | 0.03 |
| rs2297594 | 978 | 305 | 190 | 78 | 888 | 330 | 191 | 60 | T | 9 | 1.42(1.05;1.92) |  | 1.18(0.97;1.42) | 1.03(0.74;1.43) | 0.04 |
| rs2343033 | 744 | 195 | 425 | 188 | 664 | 244 | 415 | 146 | T | 22 | 1.64(1.29;2.09) |  | 1.41(1.13;1.77) | 1.21(0.94;1.56) | 0.00 |
| rs3789352 | 918 | 275 | 251 | 108 | 824 | 301 | 255 | 89 | T | 12 | 1.4(1.06;1.84) |  | 1.2(0.99;1.46) | 1.07(0.81;1.43) | 0.04 |
| rs4076930 | 230 | 69 | 849 | 321 | 217 | 90 | 951 | 293 | C | 45 | 1.37(1;1.86) |  | 1.43(0.98;2.09) | 1.13(0.83;1.55) | 0.04 |
| rs5019252 | 511 | 140 | 658 | 243 | 428 | 164 | 651 | 226 | T | 36 | 1.38(1.08;1.76) |  | 1.42(1.09;1.86) | 1.23(0.96;1.58) | 0.02 |
| rs544678 | 459 | 143 | 710 | 240 | 407 | 175 | 672 | 215 | G | 22 | 1.11(0.87;1.42) |  | 1.39(1.06;1.81) | 1.01(0.78;1.3) | 0.03 |
| rs6018397 | 430 | 112 | 739 | 271 | 363 | 153 | 716 | 237 | T | 41 | 1.34(1.03;1.73) |  | 1.57(1.18;2.11) | 1.18(0.91;1.54) | 0.01 |
| rs6021435 | 573 | 175 | 596 | 208 | 517 | 208 | 562 | 182 | C | 28 | 1.16(0.91;1.48) |  | 1.34(1.05;1.7) | 1.02(0.79;1.3) | 0.03 |
| rs6033082 | 447 | 128 | 629 | 261 | 430 | 162 | 738 | 221 | T | 38 | 1.51(1.18;1.95) |  | 1.39(1.06;1.84) | 1.09(0.84;1.41) | 0.00 |
| rs6065547 | 450 | 128 | 719 | 255 | 375 | 162 | 704 | 228 | C | 21 | 1.24(0.97;1.6) |  | 1.48(1.12;1.96) | 1.11(0.86;1.44) | 0.02 |
| rs6085113 | 390 | 116 | 779 | 267 | 374 | 159 | 705 | 231 | G | 19 | 1.16(0.89;1.5) |  | 1.42(1.06;1.89) | 1.07(0.82;1.4) | 0.04 |
| rs6089219 | 159 | 47 | 920 | 343 | 158 | 69 | 1011 | 314 | G | 7 | 1.36(0.95;1.94) |  | 1.75(1.12;2.75) | 1.13(0.79;1.62) | 0.03 |
| rs6094017 | 112 | 25 | 967 | 365 | 95 | 40 | 1074 | 342 | T | 30 | 1.9(1.19;3.03) |  | 2.28(1.26;4.12) | 1.61(1.01;2.57) | 0.04 |
| rs6101739 | 418 | 118 | 751 | 265 | 376 | 154 | 703 | 236 | G | 41 | 1.23(0.95;1.59) |  | 1.44(1.08;1.93) | 1.13(0.87;1.48) | 0.03 |
| rs6109800 | 528 | 144 | 640 | 239 | 465 | 178 | 614 | 212 | C | 34 | 1.34(1.05;1.71) |  | 1.39(1.07;1.8) | 1.2(0.93;1.54) | 0.03 |
| rs6112999 | 407 | 123 | 672 | 267 | 396 | 144 | 773 | 239 | G | 41 | 1.27(0.99;1.64) |  | 1.24(0.93;1.65) | 1(0.78;1.3) | 0.03 |
| rs6122160 | 483 | 124 | 686 | 259 | 415 | 165 | 664 | 225 | T | 37 | 1.53(1.19;1.97) |  | 1.59(1.2;2.1) | 1.3(1;1.68) | 0.00 |
| rs6126017 | 277 | 82 | 802 | 308 | 279 | 122 | 890 | 261 | C | 50 | 1.32(0.99;1.76) |  | 1.51(1.08;2.11) | 1.03(0.77;1.38) | 0.01 |
| rs6127084 | 777 | 238 | 392 | 145 | 723 | 274 | 356 | 116 | G | 18 | 1.3(1.02;1.67) |  | 1.24(1.01;1.53) | 1.07(0.82;1.4) | 0.04 |
| rs6130216 | 826 | 231 | 343 | 151 | 751 | 266 | 327 | 124 | T | 17 | 1.54(1.2;1.98) |  | 1.28(1.03;1.57) | 1.21(0.93;1.58) | 0.02 |
| rs6133208 | 1058 | 324 | 111 | 59 | 926 | 342 | 153 | 48 | A | 6 | 1.75(1.23;2.48) |  | 1.18(0.98;1.42) | 1.01(0.71;1.45) | 0.01 |
| rs7264396 | 631 | 198 | 448 | 192 | 694 | 234 | 474 | 149 | T | 24 | 1.4(1.1;1.78) |  | 1.1(0.88;1.38) | 1.05(0.82;1.36) | 0.04 |
| rs8115958 | 293 | 77 | 876 | 306 | 255 | 107 | 824 | 283 | G | 13 | 1.38(1.03;1.86) |  | 1.67(1.18;2.38) | 1.3(0.97;1.76) | 0.02 |
| rs8116375 | 329 | 99 | 840 | 284 | 294 | 130 | 785 | 260 | A | 15 | 1.09(0.83;1.43) |  | 1.48(1.08;2.03) | 1.03(0.78;1.36) | 0.04 |
| rs844885 | 229 | 71 | 850 | 319 | 220 | 90 | 949 | 293 | A | 11 | 1.3(0.96;1.76) |  | 1.42(0.98;2.07) | 1.08(0.79;1.46) | 0.04 |
| chr21 |  |  |  |  |  |  |  |  |  |  |  |  |  |  |  |
| rs1122423 | 319 | 90 | 838 | 290 | 285 | 139 | 785 | 248 | G | 15 | 1.27(0.96;1.67) |  | 1.7(1.23;2.35) | 1.13(0.85;1.5) | 0.01 |
| rs11702844 | 273 | 83 | 806 | 307 | 268 | 113 | 901 | 270 | G | 13 | 1.29(0.97;1.73) |  | 1.43(1.01;2.02) | 1.04(0.78;1.4) | 0.02 |
| rs1551588 | 334 | 96 | 834 | 287 | 268 | 124 | 811 | 265 | A | 15 | 1.22(0.93;1.61) |  | 1.58(1.15;2.19) | 1.13(0.86;1.49) | 0.02 |
| rs1557275 | 453 | 133 | 716 | 250 | 374 | 162 | 705 | 228 | G | 21 | 1.2(0.94;1.54) |  | 1.46(1.11;1.93) | 1.08(0.84;1.39) | 0.02 |
| rs208894 | 338 | 95 | 741 | 295 | 354 | 119 | 815 | 264 | T | 45 | 1.46(1.11;1.92) |  | 1.29(0.93;1.77) | 1.19(0.9;1.57) | 0.04 |
| rs2211813 | 456 | 134 | 713 | 249 | 380 | 163 | 699 | 227 | T | 21 | 1.22(0.95;1.56) |  | 1.46(1.11;1.92) | 1.09(0.85;1.4) | 0.02 |
| rs2212010 | 573 | 152 | 596 | 231 | 466 | 177 | 613 | 213 | A | 33 | 1.57(1.23;2) |  | 1.45(1.12;1.88) | 1.34(1.05;1.72) | 0.01 |
| rs2284613 | 623 | 188 | 546 | 195 | 568 | 222 | 511 | 168 | G | 31 | 1.22(0.96;1.55) |  | 1.31(1.04;1.65) | 1.06(0.82;1.35) | 0.03 |
| rs232405 | 900 | 278 | 269 | 105 | 806 | 309 | 273 | 81 | C | 13 | 1.33(1.01;1.75) |  | 1.21(0.99;1.47) | 1(0.75;1.34) | 0.03 |
| rs2821973 | 375 | 105 | 793 | 278 | 332 | 136 | 747 | 253 | C | 44 | 1.29(0.99;1.69) |  | 1.5(1.11;2.04) | 1.2(0.91;1.57) | 0.03 |
| rs2823088 | 690 | 225 | 388 | 165 | 716 | 240 | 453 | 143 | A | 21 | 1.4(1.09;1.78) |  | 1.07(0.86;1.33) | 1.03(0.8;1.32) | 0.04 |
| rs2835369 | 221 | 68 | 858 | 322 | 248 | 106 | 921 | 277 | A | 11 | 1.27(0.93;1.73) |  | 1.42(0.98;2.05) | 1.04(0.76;1.43) | 0.04 |
| rs2835370 | 218 | 68 | 861 | 322 | 250 | 107 | 919 | 276 | C | 11 | 1.25(0.91;1.71) |  | 1.42(0.98;2.05) | 1.02(0.75;1.41) | 0.04 |
| rs2835374 | 224 | 68 | 855 | 322 | 250 | 106 | 919 | 277 | T | 11 | 1.29(0.94;1.77) |  | 1.42(0.99;2.06) | 1.06(0.77;1.46) | 0.04 |
| rs2836070 | 979 | 298 | 190 | 85 | 879 | 323 | 200 | 67 | T | 10 | 1.56(1.16;2.1) |  | 1.19(0.99;1.44) | 1.09(0.8;1.5) | 0.03 |
| rs3788147 | 854 | 245 | 315 | 138 | 740 | 279 | 339 | 111 | C | 17 | 1.37(1.06;1.78) |  | 1.25(1.02;1.54) | 1.06(0.81;1.39) | 0.02 |
| rs4817363 | 478 | 140 | 691 | 243 | 418 | 173 | 661 | 217 | T | 37 | 1.24(0.97;1.59) |  | 1.39(1.07;1.82) | 1.12(0.87;1.45) | 0.03 |
| rs6517774 | 373 | 112 | 706 | 278 | 414 | 145 | 755 | 238 | G | 41 | 1.4(1.07;1.81) |  | 1.25(0.93;1.68) | 1.12(0.86;1.47) | 0.04 |
| rs724208 | 232 | 68 | 847 | 322 | 217 | 87 | 951 | 296 | A | 44 | 1.28(0.95;1.74) |  | 1.46(1;2.13) | 1.06(0.78;1.45) | 0.04 |
| rs7278204 | 879 | 263 | 290 | 120 | 800 | 301 | 279 | 89 | G | 14 | 1.39(1.07;1.81) |  | 1.24(1.02;1.51) | 1.03(0.78;1.37) | 0.02 |
| rs973754 | 878 | 262 | 291 | 121 | 801 | 301 | 278 | 89 | G | 14 | 1.39(1.07;1.81) |  | 1.24(1.01;1.51) | 1.04(0.78;1.38) | 0.02 |
| rs9976018 | 747 | 210 | 422 | 173 | 670 | 256 | 409 | 134 | T | 21 | 1.35(1.06;1.72) |  | 1.29(1.04;1.6) | 1.11(0.86;1.44) | 0.02 |
| rs9980261 | 591 | 177 | 578 | 206 | 513 | 204 | 566 | 186 | T | 30 | 1.22(0.96;1.55) |  | 1.36(1.07;1.73) | 1.05(0.82;1.35) | 0.02 |
| ch 22 |  |  |  |  |  |  |  |  |  |  |  |  |  |  |  |
| rs1009148 | 934 | 278 | 235 | 105 | 838 | 307 | 241 | 83 | C | 12 | 1.45(1.1;1.92) |  | 1.2(0.99;1.46) | 1.09(0.81;1.47) | 0.04 |
| rs17298479 | 296 | 94 | 783 | 296 | 318 | 122 | 851 | 261 | A | 15 | 1.33(1;1.75) |  | 1.31(0.94;1.81) | 1.08(0.82;1.43) | 0.04 |
| rs2858729 | 495 | 143 | 674 | 240 | 421 | 168 | 658 | 222 | T | 37 | 1.25(0.98;1.6) |  | 1.41(1.08;1.84) | 1.13(0.88;1.45) | 0.03 |
| rs2899222 | 193 | 44 | 976 | 339 | 162 | 73 | 916 | 316 | A | 39 | 1.54(1.07;2.22) |  | 2.11(1.35;3.28) | 1.47(1.02;2.12) | 0.02 |
| rs3213527 | 595 | 180 | 484 | 210 | 615 | 216 | 554 | 167 | C | 27 | 1.51(1.18;1.91) |  | 1.21(0.96;1.54) | 1.05(0.81;1.34) | 0.00 |
| rs4925429 | 826 | 254 | 341 | 129 | 774 | 290 | 305 | 100 | T | 16 | 1.25(0.97;1.61) |  | 1.21(0.99;1.49) | 1(0.76;1.32) | 0.04 |
| rs5750715 | 519 | 168 | 560 | 222 | 570 | 201 | 599 | 182 | A | 29 | 1.35(1.06;1.72) |  | 1.15(0.9;1.48) | 1.03(0.8;1.32) | 0.03 |
| rs5756540 | 741 | 226 | 428 | 157 | 692 | 267 | 387 | 123 | A | 20 | 1.25(0.98;1.59) |  | 1.26(1.02;1.56) | 1.01(0.78;1.32) | 0.02 |
| rs5757761 | 213 | 52 | 866 | 338 | 224 | 84 | 945 | 299 | C | 44 | 1.58(1.13;2.21) |  | 1.5(1;2.26) | 1.32(0.94;1.85) | 0.04 |
| rs6003160 | 584 | 170 | 585 | 213 | 528 | 220 | 551 | 170 | G | 30 | 1.22(0.96;1.55) |  | 1.37(1.08;1.74) | 1.03(0.8;1.32) | 0.01 |
| rs695288 | 311 | 89 | 858 | 294 | 295 | 130 | 784 | 260 | C | 15 | 1.25(0.95;1.66) |  | 1.53(1.1;2.12) | 1.18(0.89;1.57) | 0.04 |
| rs8136975 | 191 | 43 | 978 | 340 | 135 | 62 | 944 | 328 | T | 36 | 1.46(1.01;2.11) |  | 2.02(1.27;3.21) | 1.43(0.99;2.07) | 0.03 |

^*^carotid intima-media thickness

^†^ multidimensional scaling
